# Comparative proteomic and transcriptomic approaches to address the active role of GA_4_ in Japanese apricot flower bud dormancy release

**DOI:** 10.1093/jxb/ert284

**Published:** 2013-09-07

**Authors:** Weibing Zhuang, Zhihong Gao, Liangju Wang, Wenjun Zhong, Zhaojun Ni, Zhen Zhang

**Affiliations:** College of Horticulture, Nanjing Agricultural University, No. 1 Weigang, Nanjing 210095, PR China

**Keywords:** 2-DE, DGE, dormancy, GA4, Japanese apricot, proteomics, transcriptomics.

## Abstract

Hormones are closely associated with dormancy in deciduous fruit trees, and gibberellins (GAs) are known to be particularly important. In this study, we observed that GA_4_ treatment led to earlier bud break in Japanese apricot. To understand better the promoting effect of GA_4_ on the dormancy release of Japanese apricot flower buds, proteomic and transcriptomic approaches were used to analyse the mechanisms of dormancy release following GA_4_ treatment, based on two-dimensional gel electrophoresis (2-DE) and digital gene expression (DGE) profiling, respectively. More than 600 highly reproducible protein spots (*P*<0.05) were detected and, following GA_4_ treatment, 38 protein spots showed more than a 2-fold difference in expression, and 32 protein spots were confidently identified according to the databases. Compared with water treatment, many proteins that were associated with energy metabolism and oxidation–reduction showed significant changes after GA_4_ treatment, which might promote dormancy release. We observed that genes at the mRNA level associated with energy metabolism and oxidation–reduction also played an important role in this process. Analysis of the functions of the identified proteins and genes and the related metabolic pathways would provide a comprehensive proteomic and transcriptomic view of the coordination of dormancy release after GA_4_ treatment in Japanese apricot flower buds.

## Introduction

Dormancy of deciduous fruit trees in temperate zones is a phase of development that allows trees to survive unfavourable conditions during winter (Faust *et al.*, 1991). This period is sensed by the accumulation of chilling hours, leading to dormancy release, and differs among tree species (Lang *et al.*, 1987; Arora *et al.*, 2003). If the chilling requirement is not satisfied, plants can suffer from an uneven and delayed bud break, reduced shoot vigour, limited anthesis, and poor flower development (Campbell and Sugano, 1975). Therefore, to elucidate the molecular mechanism of dormancy release in Japanese apricot is of importance for both plant biology and crop development.

Japanese apricot (*Prunus mume* Sieb. et Zucc) originated in China and has been widely cultivated in Asia for about 3000 years (Chu, 1999). It is the earliest fruit tree in the *Rosaceae* family to bloom. Stone-fruit crops, including Japanese apricot, require a certain chilling accumulation during the winter to release dormancy. Due to its broad chilling requirement range, Japanese apricot has uneven flowering, making it difficult to obtain stable fruit production in either protected or orchard cultivation in the southern areas of China. In addition, the southern areas of China frequently have warm winters, and the chilling requirement of some Japanese apricot cultivars cannot be adequately satisfied, requiring growers to apply dormancy-breaking reagents to obtain uniform flowering. This situation is caused by irregular lateral bud endodormancy release, possibly due to global warming (Sugiura *et al.*, 2007). As a result, the genetic factors that control endodormancy have been investigated, to try to understand the molecular basis of endodormancy regulation in temperate fruit tree species, which might lead to the artificial control of endodormancy through spraying with dormancy-breaking reagents.

Several chemicals can be used to induce bud break of deciduous fruit trees in areas lacking sufficient chilling conditions. Hydrogen cyanamide (Dormex), potassium nitrate (KNO_3_), and mineral oil have a synergistic effect on bud break, as well as some of the chemical constituents of many deciduous fruit trees (Sagredo *et al.*, 2005; de Oliveira *et al.*, 2008; Sabry *et al.*, 2011). Gibberellins (GAs) are particularly important, and might function in the timing of dormancy establishment and chilling-induced release (Schrader *et al.*, 2004). Exogenous applications of GAs often induce dormancy break in a wide variety of woody angiosperms (Looney, 1997). Saure (1985) also reported that GA application can substitute for chilling in dormancy release.

Recent studies have suggested that bud burst is dependent on sufficient GA levels, and that a reduction in active GAs rather than reduced GA sensitivity plays a major role in growth cessation (Hoffman, 2011). Some evidence also indicates that GA biosynthesis genes are induced by long-term chilling exposure in dormant buds and are therefore associated with the acquisition of growth ability (Rinne *et al.*, 2011). Barros *et al.* (2012) suggested that a change in GA metabolism results from the differential regulation of at least *PdGA20OX* and *PdGA2OX* after flower bud break. *Populus* trees with lower levels of active GAs due to overexpression of the catabolic enzyme GA 2-oxidase or impaired perception by overexpression of *GA INSENSITIVE* or *RGA-like* receptors, showed early bud set and late bud burst (Zawaski *et al.*, 2011). In addition, cold night temperatures combined with the inhibition of GA accumulation were sufficient to induce ecodormancy and bud set in *PHYA* overexpressing poplar lines (Mølmann *et al.*, 2005). Rinne *et al.* (2011) reported that GA application can substitute for chilling in dormancy release, suggesting that chilling recruited GA in dormant buds and that a different response of GA_3_ and GA_4_ occurred in dormancy break, and that only GA_4_ induced bud burst (Rinne *et al.*, 2011). The consequences of the expression of GA biosynthesis genes have been studied in bud burst, but the interplay between GA_4_ treatment with gene expression, protein synthesis, and activation in causing physiological changes remains to be elucidated.

Many researchers have studied the mechanism of dormancy in deciduous fruit trees using suppression subtractive hybridization, which is limited in terms of how many genes can be explored relating to this process (Leida *et al.*, 2010; Li *et al.*, 2011). Increasing numbers of studies have begun to apply custom microarray or transcript profiling to investigate the mechanism of dormancy in perennial plants (Mazzitelli *et al.*, 2007; Mathiason *et al.*, 2009; Hedley *et al.*, 2010). With the development of ‘omic’ technologies, such as transcriptomics, metabolomics, and proteomics, these have been used in combination to reveal the complexity of the physiological processes and to develop a comprehensive understanding of other physiological processes in fruit trees, such as growth and development.

To date, reports concerning the mechanism of dormancy release following treatment with GA_4_ have been limited at both the proteomic and the transcriptomic levels. This study aimed to investigate the promotive effect of GA_4_ treatment on dormancy release at the proteomic and transcriptomic levels, based on two-dimensional gel electrophoresis (2-DE) and digital gene expression (DGE) profiling. The characterization of these proteins and genes clearly reflected the dynamic changes in protein and gene expression patterns in Japanese apricot during the dormancy period and dormancy release when treated with GA_4_, hence increasing our knowledge of the complex mechanisms that regulate dormancy.

## Materials and methods

### Plant material and treatment

The Japanese apricot (*Prunus mume* Sieb. et Zucc) cultivar used in this study was ‘Bungo’, a late-flowering cultivar, which is grown in the ‘National Field Genebank for Japanese apricot’, located in Nanjing, Jiangsu Province, China. Long 1-year-old branches were cut from ‘Bungo’ trees on 16 December 2011, 23 December 2011, and 30 December 2011, and GA_4_ was supplied to the buds at a concentration of 100 μM GA_4_ via the stem vasculature and not directly onto the bud according to the method described by Rinne *et al.* (2011). Their basal parts were placed in water containing GA_4_ or in water without GA_4_ as a control and incubated in a growth chamber. The branches were maintained at 25±1 °C under white fluorescent tubes (55 μmol m^−2^ s^−1^) with a 16:8h light:dark photoperiod at 18±1 °C and a constant relative humidity of 70%. After 2 d, the solution was changed and the basal branches were recut. The branches were maintained in the growth chamber for 10 d to test the percentage of Japanese apricot flower bud break. According to the classification of different phenological growth stages described by Baggiolini (1952) with some slight modifications, when 50% of the flower buds on the branch cuttings were in the green tip stage (Supplementary Fig. S1 at *JXB* online), we considered the flower buds to have broken endodormancy. More than 120 flower buds were measured in each treatment. Flower buds were collected from the middle portions of the branches at 0 and 10 d after treatment and were immediately frozen in liquid nitrogen and stored at –70 °C for further use. The flower buds of Japanese apricot collected on 30 December 2011 were used for protein analysis. A GA_4_ or water treatment at 0 d from this date was used as a control; the GA_4_ treatment after 10 d was termed G10 and the water treatment after 10 d was W10. We used the flower buds of Japanese apricot collected on 30 December 2011 for DGE and quantitative reverse transcription-PCR (qRT-PCR) analysis, and selected the flower buds treated with GA_4_ or water after 0 d as the control and the flower buds treated with GA_4_ after 10 d. designated as A. to study the transcriptomic changes. Three replicates were used as follows: three trees were used as the plant material and three branches were cut from each tree. Two additional branches from the same tree were used as another two replicates. We mixed the flower buds of three trees to conduct the subsequent proteomic and transcriptomic study (Supplementary Fig. S2 at *JXB* online).

### Protein extraction and quantification

Protein extraction was performed according to the trichloroacetic acid/acetone precipitation method, as described by Zhuang *et al.* (2011). Briefly, ~0.7g of flower bud powder was homogenized in 5ml cold acetone [containing 10% trichloroacetic acid and 0.07% dithiothreitol (DTT)] and then precipitated overnight at –20 °C. The homogenate was centrifuged at 15 000*g* for 0.5h at 4 °C. The pellet was washed with 5ml of cold acetone (containing 0.07% DTT) and recentrifuged at 15 000*g* for 0.5h at 4 °C. The centrifugation steps were repeated until the supernatant was colourless, and the pellet was then air dried at 4 °C and stored at –70 °C for further use. The protein powder was resuspended in sample rehydration buffer [7M urea, 4% (w/v) CHAPS, 65mM DTT, 0.2% (v/v) 3–10 and 4–7 ampholytes (Amersham), 2M thiourea, and 0.001% bromophenol blue]. The protein concentration was determined according to the method described by Bradford (1976).

### 2-DE gels and staining

Sample aliquots containing 1.3mg of proteins were applied to 17cm ReadyStrip IPG Strips (Bio-Rad), and isoelectric focusing was performed on a PROTEAN isoelectric focusing system (Bio-Rad) for a total of 60 kVh at 19 °C. After isoelectric focusing, the strips were equilibrated for 15min in equilibration solution I [6M urea, 0.375M Tris/HCl (pH 8.8), 2% (w/v) SDS, 20% (v/v) glycerol, 2% (w/v) DTT] and for another 15min in equilibration solution II [6M urea, 0.375M Tris/HCl (pH 8.8), 2% (w/v) SDS, 20% (v/v) glycerol, 2.5% (w/v) iodoacetamide]. Following equilibration, the strips were run on an Ettan Six Vertical set (GE Healthcare) in running buffer (25mM Tris/HCl, 192mM glycine, 0.1% SDS) at 16 °C with a cooling device (GE Healthcare). The gels were run at 1W per gel for 1.5h, and then at 15W per gel until the bromophenol blue reached the bottom of the plate. The gels were then stained with colloidal Coomassie Brilliant Blue G250.

### Image acquisition and data analysis

The stained gels were scanned using a Versdoc 3000 scanner (Bio-Rad), and analysed using the PDQuest 8.0 software (Bio-Rad). Each gel was analysed for image filtration, spot detection and intensity quantification, background subtraction, spot matching, and quantitative intensity measurement. Briefly, images were properly cropped and optimized with advanced crop. Then, we chose the ‘control’ as a master gel, and spots in the master gel were matched across all other treatment gels. The protein spots were detected using the following parameters: sensitivity 8.31, size scale 9, min peak value 553, vertical streaking 57, horizontal streaking 68, and large spot size 67×57. Each image was checked manually to remove false spots and to add missed spots. Sixty landmark spots, which were well resolved and present in all members of the matchset, were used to align and position all members of the matchset. The spot patterns of the different gels were automatically matched to each other, and each spot was given a unique identification number (SSP). Following matching, all gel spots were normalized by the local regression model (LOESS) method. The replicate gels used for making the matchset had a correlation coefficient value of at least 0.8. The spots that were present on at least two gels of one treatment or the control based on the image analysis were identified as expressed protein spots. Quantitative analyses were carried out after normalizing the spot quantities [as spot optical density (OD)] in all gels in order to compensate for gel-to-gel variations due to loading, gel staining, destaining, and imaging, and the individual protein spot quantities were normalized as a percentage of the total quantity of valid spots present in the gel. For each protein spot, the mean spot quantity value and its variance coefficient in each group was determined. A quality score of <30 was adopted to deﬁne low-quality spots, which were eliminated in further analysis (Bhushan *et al.*, 2007), and the saturated spots were also removed. Quantitative comparisons of the gels between different treatments were used to determine significantly differentially expressed spots, and only spots that showed at least a 2-fold change in expression and that were statistically significant in a one-way analysis of variance (*P*<0.05) that tested for reproducible changes in three analytical replicates were considered for subsequent analysis. Principal component analysis was also performed to show the significance among the differential expressed proteins. Experimental molecular weights (MW) and isoelectric points (pI) were calibrated according to the MW marker proteins.

### Protein identification and database search

Protein spots of interest were washed with 25mM NH_4_HCO_3_ followed by dehydration with 50% acetonitrile in 25mM NH_4_HCO_3_, reduction with 10mM DTT in 50mM NH_4_HCO_3_ for 1h at 56 °C, and alkylation in 55mM iodoacetamide in 50mM NH_4_HCO_3_ for 1h at room temperature. The protein spots were washed several times with 50mM NH_4_HCO_3_ followed by dehydration with acetonitrile before finally being dried in a vacuum centrifuge and digested overnight at 37 °C by the addition of 1.5ml of trypsin. The resulting peptides were extracted by washing the protein spots with 0.1% trifluoroacetic acid in 67% acetonitrile and analysed using a 4800 matrix-assisted laser desorption/ionization time-of-flight/time-of-flight (MALDI-TOF/TOF) Proteomics Analyzer (Applied Biosystems). The HCCA matrix was used for the mass spectrometry (MS) analyses.

An MS/MS ion search was performed using GPS Explorert™ software v3.5 (Applied Biosystems) in a local library built from the entire peach proteome database (http://www.rosaceae.org/node/355; PPA database, 28 702 sequences and 11 557 397 residues) using the MASCOT search engine v3.5 (Matrix Science, London, UK). If no credible candidate could be matched, the NCBI nr 20120421 database (17 910 093 sequences, 614 7033 692 residues) was then searched. The search parameters were taxonomically restricted to the Viridiplantae and to one missed cleavage, 50 ppm mass tolerance in MS, and 0.2Da in MS/MS, cysteine carbamidomethylation as a fixed modification, and methionine oxidation as a variable modification. A total ion score in the PPA database or in the NCBI nr 20120421 database that significantly (*P*<0.05) exceeded the MASCOT identity or extensive homology threshold indicated successful protein identification. If a spot was identified in both of the databases, we used the higher MOWSE score to positively identify the protein and/or peptide. When the two proteins had the same MOWSE score, information such as the number of matched peptides (≥2), sequence coverage, MW, and pI were considered to select the protein of interest from them. The proteins that had a higher MOWSE score, more matched peptides (≥2) and sequence coverage, and a better correlation between experimental and theoretical MW and pI in the sequence were accepted as being unambiguously identified.

### Total RNA extraction

Total RNA was extracted from frozen flower bud material of Japanese apricot (100mg) using a cetyltrimethyl ammonium bromide method (Pavy *et al.*, 2008) with some modifications. Genomic DNA contamination was removed with RNase-free DNase I (TaKaRa), according to the manufacturer’s instructions. The RNA concentration was calculated from the absorbance at 260nm (*A*_260_) with a BioPhotometer (Eppendorf). Purity was verified by an *A*_260_/*A*_280_ ratio of between 1.80 and 2.05, and *A*_260_/*A*_230_ nm values ranging from 2.00 to 2.60; the integrity was evaluated by electrophoresis on ethidium bromide-stained 1.0% agarose gels. The RNA of the Japanese apricot flower bud was stored at –70 °C until further use.

### DGE profile

The DGE process includes sample preparation and sequencing. The main instruments used were the Illumina Cluster Station and the Illumina HiSeq™ 2000 System, and the main reagents and supplies were the Illumina Gene Expression Sample Prep Kit and the Solexa Sequencing Chip (flow cell). Samples (20 μg) of the total RNA of Japanese apricot flower buds were sent to BGI-Shenzhen (China) for further analysis according to their pipeline experiment (Supplementary Fig. S3 at *JXB* online). Six micrograms of total RNA was extracted, and oligo(dT) magnetic bead adsorption was used to purify the mRNA; oligo(dT)s were then used as primers to synthesize the first- and second-strand cDNA. The 5′ ends of tags could be produced by two types of endonuclease: *Nla*III or *Dpn*II. The bead-bound cDNA was subsequently digested by the restriction enzyme *Nla*III, which recognized and cut the CATG sites. The fragments (except for the 3′ cDNA fragments) connected to oligo(dT) beads were washed away and the Illumina adaptor 1 was ligated to the sticky 5′ end of the digested bead-bound cDNA fragments. The junction of the Illumina adaptor 1 and the CATG site was the recognition site of *Mme*I, which is a type of endonuclease with separate recognition and digestion sites. This enzyme cut 17bp downstream of the CATG site, producing tags with adaptor 1 ends. After removing 3′ fragments with magnetic bead precipitation, the Illumina adaptor 2 was ligated to the 3′ ends of tags, acquiring tags with different adaptors at both ends to form a tag library. After 15 cycles of linear PCR amplification, 105bp fragments were purified by 6% TBE-PAGE gel electrophoresis. After denaturation, the single-chain molecules were fixed onto the Illumina Sequencing Chip (flow cell). Each molecule grew into a single-molecule cluster sequencing template through *in situ* amplification, and four types of nucleotides that were labelled with four colours were added before sequencing was performed using the method of sequencing-by-synthesis. Sequencing-by-synthesis technology uses four fluorescently labelled nucleotides to sequence the tens of millions of clusters on the flow cell surface in parallel. During each sequencing cycle, a single labelled dNTP is added to the nucleic acid chain. The nucleotide label serves as a terminator for polymerization, so that after each dNTP incorporation, the fluorescent dye is imaged to identify the base and then cleaved enzymatically to allow incorporation of the next nucleotide. As all four reversible terminator-bound dNTPs (A, C, T, and G) are present as single, separate molecules, natural competition minimizes incorporation bias. Base calls are made directly from signal intensity measurements during each cycle, which greatly reduces raw error rates compared with other technologies. The end result is highly accurate base-by-base sequencing that eliminates sequence-context-specific errors, enabling robust base calling across the genome, including repetitive sequence regions and within homopolymers. Each tunnel generated millions of raw reads with a sequence length of 49bp.

### Analysis and screening of DGE data

Sequencing-received raw image data were transformed by base calling into sequence data, which are called raw data or raw reads, and were stored in FASTQ format. Information about read sequences and quality is stored in this type of file; each read is described in four lines in FASTQ files. Clean tags were generated by removing the 3′ adaptor sequence, empty reads (reads with a 3′ adaptor sequence but no tag), low-quality tags (tags with unknown N′ sequences), tags that were too long or too short, and tags with a copy number of one. All tags were annotated using the database provided by Illumina. Briefly, a virtual library was constructed containing all of the possible CATG+17 base length sequences of the reference gene sequences obtained from the peach genome and transcriptome. All clean tags were mapped to the reference sequences and only 1bp mismatches were considered. Clean tags that mapped to reference sequences from multiple genes were filtered. The remaining clean tags were designated as unambiguous clean tags, and the number of these was calculated for each gene and then normalized to the number of transcripts per million clean tags (’t Hoen *et al.*, 2008; Morrissy *et al.*, 2009). Genes expressed differentially in two samples were analysed as described previously (Audic and Claverie, 1997). We used a false discovery rate of ≤0.001 and the absolute value of |log_2_ratio|≥1 as the threshold upon which to judge the significance of gene expression differences. More stringent criteria with smaller false discovery rates and greater fold-change values can be used to identify differentially expressed genes. The statistical analysis was performed on the DGE data using SPSS software, with the threshold for *P* values set as 0.05.

### qRT-PCR validation

The expression of candidate genes was determined using qRT-PCR. A sample of total RNA (1 μg) was reverse transcribed for ﬁrst-strand cDNA synthesis using a ReverTra Ace qPCR RT Kit (Toyobo), according to the manufacturer’s instructions. Gene-specific primers were designed using Primer Premier 5.0 software according to the sequence of the target gene in the PPA database (Supplementary Table S1 at *JXB* online). qRT-PCR was carried out on an Applied Biosystems 7300 Real Time PCR System with a 20 μl reaction volume, containing 1 μl of 10-fold-diluted cDNA, 0.3 μl (10 pM) of each primer, 10 μl of SYBR® Premix Ex Taq™ (Perfect Real Time; TaKaRa). and 8.4μl of sterile double-distilled water. The thermal cycling program was 95 °C for 3min, followed by 40 cycles of 95 °C for 25 s, 62 °C for 25 s, and 72 °C for 40 s. RNA polymerase II was used to normalize gene expression (Tong *et al.*, 2009). The relative expression levels of genes were analysed using the 7300 system software and the 2^–ΔΔ*C*t^ method, which represents the difference of the cycle threshold (*C*_t_) between the control RNA polymerase II products and target gene products. Data analyses were conducted using SPSS version 17.0 statistical software. Triplicate samples were used for qRT-PCR.

### Correlation analysis of proteomics and transcriptomics

The correlation analysis of proteomics and transcriptomics included the results of the transcription analysis and the protein 2-DE analysis to assess the potential relevance of quantitative information between genes and proteins. As the number of differentially expressed proteins was very limited, the correlation analysis was conducted between differentially expressed proteins and genes in the whole library.

## Results

### GA_4_ treatment can lead to earlier and higher bud-break levels

**Fig. 1.**
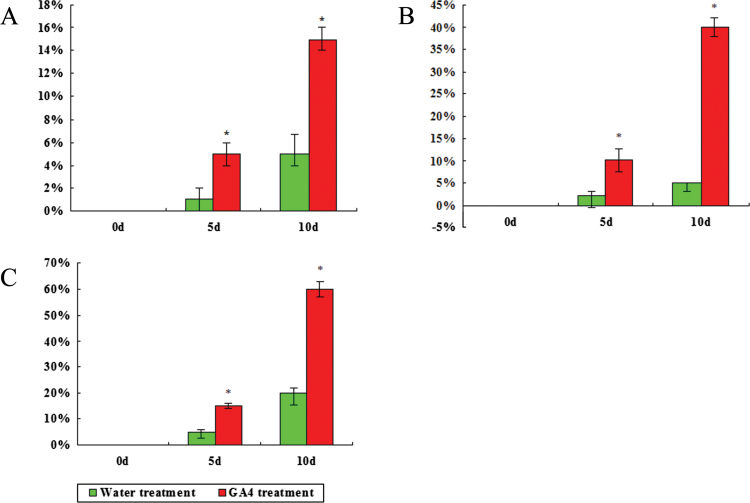
Effect of GA_4_ treatment on the percentage of bud break in Japanese apricot. After the branches were collected, they were placed in GA_4_ solution or water as a control and the percentage of bud break at 0, 5, and 10 d was measured. The percentage of bud break was a mean value of three measurements after GA_4_ treatment and water treatment at each time point. Results are shown for samples collected on 16 December 2011 (A), 23 December 2011 (B) and 30 December 2011 (C). Asterisks indicate values that differ significantly at each time point (pairwise Student’s t-test, P<0.05). (This figure is available in colour at *JXB* online.)

Figure 1 shows the effect of GA_4_ treatment on the bud dormancy release of Japanese apricot. Compared with the water treatment, GA_4_ treatment promoted the rate of bud burst in Japanese apricot. After applying GA_4_ on 30 December 2011, the percentage of Japanese apricot flower buds that had burst after 10 d reached 60%, which indicated that they had released their dormancy. However, the percentage after 10 d of water treatment only reached 20%, indicating that these samples were in the endodormancy stage. Irrespective of whether GA_4_ was applied, Japanese apricot flower buds were always in the endodormancy stage prior to 30 December 2011, but the rate of bud burst following GA_4_ treatment was much higher than in the water treatment. By 17 February 2012, the percentage of budburst after 10 d of water treatment reached 70% (data not shown), which was 49 d later than for buds treated with GA_4_.

### 2-DE analysis of Japanese apricot flower buds after GA_4_ treatment

**Fig. 2.**
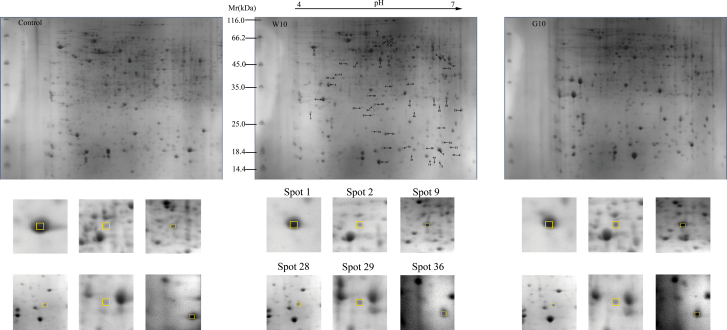
2-DE gel profiles of total proteins from the control, G10, and W10. The numbers of the 48 differentially expressed protein spots in response the GA_4_ and water treatment are marked with arrows and numbers, and the protein spot numbers corresponded to those listed in Tables 1 and 2. Magniﬁed views of some of the differentially abundant proteins are shown below. Control represents GA_4_ or water treatment after 0 d, W10 represents water treatment after 10 d, and G10 represents GA_4_ treatment after 10 d. The boxes and numbers indicate different spots in the different treatments. (This figure is available in colour at *JXB* online.)

To explore further the effect of GA_4_ treatment on the rate of bud dormancy release in Japanese apricot, a proteomic approach was applied. About 600 highly reproducible protein spots were consistently observed in all replicates after image analysis, with the pI and MW ranging from 4.0 to 7.0 and 14.4 to 70.0kDa, respectively (Fig. 2 and Supplementary Fig. S4 at *JXB* online). From these, 49 differentially expressed spots (*P*<0.05) were selected for excision and analysed using MALDI-TOF/TOF. Finally, 42 protein spots were confidently identified according to the databases (Tables 1 and 2, and Supplementary Tables S2 and S3 at *JXB* online). Magnified views of some of the numbered protein spots showing differential expression are highlighted in Fig. 2.

**Table 1.** Identification of 32 proteins associated with dormancy release treated with GA_4_ in Japanese apricot

Spot no.^a^	Protein (taxonomy)	Accession no.^b^	NP^c^	Theoretical MW/pI	Experimental MW/pI	Score^d^	SC (%)^e^	Average fold change^f^
Stress and defence								
1	Mal d1 homologue (*Prunus armeniaca*)	gi|2460186	4	17.60/5.79	17.54/6.43	432	28	–3.39
2	Heat-shock protein 60 (*Prunus persica*)	ppa003391	3	61.50/5.62	59.02/5.36	97	5	+2.92
3	Heat-shock protein 60 (*Prunus persica* s)	ppa004110	4	56.32/5.19	60.45/5.51	56	11	+2.59
5	class IV chitinase (*Corylus heterophylla*)	gi|344190188	2	30.03/5.21	27.21/4.24	116	6	+20.33
6	Pathogenesis-related thaumatin superfamily protein (*Prunus persica*)	ppa010479	5	26.94/4.83	37.55/4.50	125	33	+7.00
Energy metabolism								
8	Alcohol dehydrogenase 1 (*Prunus persica*)	ppa007154	8	41.88/5.93	50.63/6.45	293	27	+2.75
9	Aldolase superfamily protein (*Prunus persica*)	ppa007696	6	38.62/6.92	36.58/5.63	280	20	+3.04
10	d-3-Phosphoglycerate dehydrogenase (*Arabidopsis thaliana*)	gi|15235282	2	63.57/6.16	48.51/5.04	136	3	+2.52
11	*myo*-Inositol-1-phosphate synthase 2 (*Prunus persica*)	ppa004430	7	56.59/5.96	58.31/5.62	117	35	–2.55
12	Triosephosphate isomerase, putative (*Ricinus communis*)	gi|255584863	3	27.66/5.89	31.52//6.34	198	14	+2.54
13	Ribulose-1,5-bisphosphate carboxylase/oxygenase large subunit (*Aloe viguieri*)	gi|33636009	5	50.60/6.43	22.62/6.42	320	11	+5.06
15	Ribulose-1,5-bisphosphate carboxylase/oxygenase large subunit (*Calystegia sepium*)	gi|21634023	4	53.10/6.43	17.93/5.84	343	10	+0
Protein metabolism								
16	Glutamine synthetase (*Lithospermum erythrorhizon*)	gi|4650846	2	69.85/9.60	44.71/6.50	179	44	+2.43
17	Elongation factor Tu (ISS) (*Ostreococcus tauri*)	gi|308804561	2	45.89/6.00	49.58/6.53	128	6	+2.65
18	Insulinase (Peptidase family M16) protein (*Prunus persica*)	ppa004554	7	53.82/5.85	58.44/5.57	218	17	+2.68
19	Eukaryotic aspartyl protease family protein (*Prunus persica*)	ppa004726	8	53.66/5.45	43.68/4.77	155	19	+2.85
20	N-terminal nucleophile aminohydrolases (Ntn hydrolases) superfamily protein (*Prunus persica*)	ppa011112	5	24.76/6.51	29.58/6.43	188	32	+3.4
21	Kunitz family trypsin and protease inhibitor protein (*Prunus persica*)	ppa011448	4	23.07/5.20	21.92/5.32	249	19	+0
Oxidation-reduction								
22	Glutathione peroxidase 6 (*Prunus persica*)	ppa010771	7	26.23/9.20	19.18/6.50	168	30	+2.27
23	Manganese superoxide dismutase (*Prunus persica*)	gi|374671153	6	26.08/8.57	27.78/6.80	658	29	+2.60
24	Ascorbate peroxidase 1 (*Prunus persica*)	ppa010413	6	27.46/5.77	29.58/5.32	383	36	–2.18
25	Ascorbate peroxidase 1 (*Prunus persica*)	ppa010413	6	27.46/5.77	29.07/5.12	315	36	–7.13
26	Ascorbate peroxidase 1 (*Prunus persica*)	ppa010413	7	27.46/5.77	31.91/6.54	298	39	+6.04
27	Peroxidase superfamily protein (*Prunus persica*)	ppa007748	3	39.53/5.16	47.57/6.59	36	12	+7.67
28	Copper/zinc-superoxide dismutase (*Prunus persica*)	gi|381283804	2	22.36/6.19	18.23/5.49	151	22	+2.71
29	Polyphenol oxidase (*Prunus salicina* var. *cordata*)	gi|331272014	4	64.91/6.48	60.93/5.55	204	8	+2.73
30	Ascorbate peroxidase 1 (*Prunus persica*)	ppa010413	7	27.46/5.77	30.52/5.37	377	39	–9.38
Cell structure								
31	Tubulin α-2 chain (*Prunus persica*)	ppa005617	8	50.30/4.96	36.86/5.92	411	20	+3.58
32	Actin 7 (*Prunus persica*)	ppa007242	7	41.93/5.31	49.42/5.46	652	30	+3.63
33	Actin-depolymerizing factor (*Malus*×*domestica*)	gi|33772153	2	11.19/8.76	18.29/6.61	104	22	+2.53
Signalling and transcription								
35	Calreticulin 1a (*Prunus persica*)	ppa006226	6	48.45/4.40	58.35/4.25	203	15	–2.66
Unclassiﬁed								
36	Cyanase (*Prunus persica*)	ppa012598	6	18.38/6.22	16.73/6.79	116	45	+2.68

^*a*^ Numbering corresponds to the 2-DE gel in Fig. 2.

^*b*^ Accession numbers from the PPA database and the NCBI nr 20120421 database.

^*c*^ The total number of peptides identified.

^*d*^ MOWSE score probability (protein score) for the entire protein.

^*e*^ Sequence coverage.

^*f*^ Average fold change: spot abundance is expressed as the ratio of intensities of upregulated or downregulated proteins between the water treatment after 0 and 10 d. Fold changes had *P* values<0.05. In this column, ‘+’ means upregulated, ‘–’ means downregulated, and ‘+0’ means that this protein only appears at 10 d after water treatment.

**Fig. 3.**
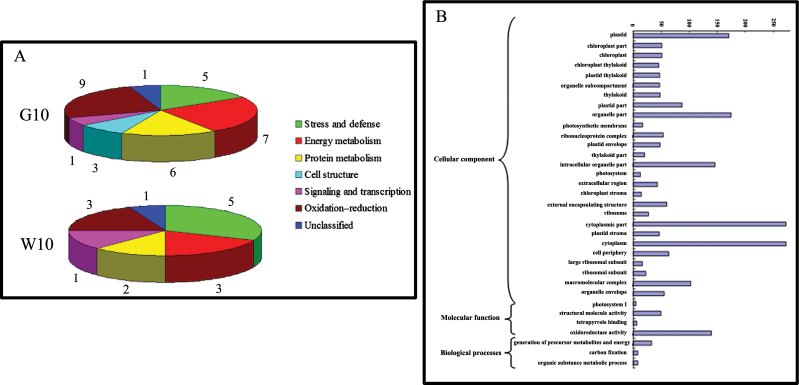
(A) Functional categorization of the differentially expressed proteins identified after 10 d of GA_4_ treatment (G10) and water treatment (W10). The digital gene expression number indicates the number of proteins in each subgroup. (B) Gene ontology functional enrichment analysis of differentially expressed genes after 10 d of GA_4_ treatment. (This figure is available in colour at *JXB* online.)

According to the metabolic and functional features described in KEGG pathways, gene ontology annotations, and the literature concerning the identified proteins or their homologies, the identified proteins could be classified into seven categories as follows: stress and defence, energy metabolism, protein metabolism, cell structure, signalling and transcription, oxidation–reduction, and unclassified (Fig. 3A). After GA_4_ treatment, 32 protein spots showed differential expression, including those of stress and defence (five, 15.6%), energy metabolism (seven, 21.9%), protein metabolism (six, 18.8%), cell structure (three, 9.4%), signalling and transcription (one, 3.1%), oxidation–reduction (nine, 28.1%), and unclassified (one, 3.1%) in G10 (Fig. 3A). When compared with the control, there were six downregulated protein spots, 24 upregulated protein spots, and two protein spots that were specifically expressed at 10 d after GA_4_ treatment (Tables 1). In contrast, stress and defence (five, 33.3%), energy metabolism (three, 20.0%), protein metabolism (two, 13.3%), signalling and transcription (one, 6.7%), oxidation–reduction (three, 20.0%), and unclassified (one, 6.7%) comprised 15 protein spots expressed differentially with water treatment (W10; Fig. 3A). After 10 d of water treatment, two protein spots were downregulated, and 13 protein spots were upregulated compared with the control (Table 2).

**Table 2.** Identification of 15 proteins associated with dormancy release treated with water in Japanese apricot

Spot no.^a^	Protein (taxonomy)	Accession no.^b^	NP^c^	Theoretical MW/ pI	Experimental MW/pI	Score^d^	SC (%)^e^	Average fold change^f^
Stress and defence								
38	MLP-like protein 423 (*Prunus persica*)	ppa012651	8	17.64/5.79	17.54/6.18	424	41	+2.75
39	Glyoxalase I homologue (*Prunus persica*)	ppa009462	6	32.64/5.27	33.45/5.23	158	20	–3.25
40	Heat-shock cognate protein 70–1 (*Prunus persica*)	ppa002646	7	71.59/5.07	41.21/4.68	303	11	+2.97
41	Mitochondrial HSO70 2 (*Prunus persica*)	ppa001973	5	79.74/8.60	65.80/5.44	243	9	+2.92
1	Mal d1 homologue (i)	gi|2460186	4	17.60/5.79	17.54/6.43	432	28	–2.53
Energy metabolism								
42	Ribulose 1,5-bisphosphate carboxylase (*Gunnera cordifolia*)	gi|18024678	5	51.67/5.95	21.41/5.56	475	17	+2.54
43	ATP synthase β subunit (*Triticum aestivum*)	gi|525291	8	59.33/5.56	49.48/5.58	700	19	+2.53
8	Alcohol dehydrogenase 1 (*Prunus armeniaca*)	ppa007154	8	41.88/5.93	50.63/6.45	293	27	+2.62
Protein metabolism								
44	FK506-binding protein 12 (*Prunus armeniaca*)	ppa013624	6	12.01/5.80	14.91/6.36	85	53	+2.91
45	5-Methyltetrahydropteroyltriglutamate- homocysteine methyltransferase, putative (*Ricinus communis*)	gi|255569484	5	84.90/6.09	29.77/5.90	497	6	+2.83
Oxidation-reduction								
46	Flavodoxin-like quinone reductase 1 (*Prunus persica*)	ppa011600	8	21.72/5.80	27.41/6.03	381	39	+2.53
23	Manganese superoxide dismutase (*Prunus persica*)	gi|374671153	6	26.08/8.57	27.78/6.80	658	29	+2.52
28	Copper/zinc- superoxide dismutase (*Prunus persica*)	gi|381283804	2	22.36/6.19	18.23/5.49	151	22	+2.15
Signalling and transcription								
47	Putative glycine- rich RNA-binding protein (*Prunus avium*)	gi|34851124	5	17.37/7.82	16.31/5.99	366	32	+2.44
Unclassiﬁed								
36	Cyanase (*Prunus persica*)	ppa012598	6	18.38/6.22	16.73/6.79	116	45	+2.50

^*a*^ Numbering corresponds to the 2-DE gel in Fig. 2.

^*b*^ Accession numbers from the PPA database and the NCBI nr 20120421 database.

^*c*^ The total number of peptides identified.

^*d*^ MOWSE score probability (protein score) for the entire protein.

^*e*^ Sequence coverage.

^*f*^ Average fold change: spot abundance is expressed as the ratio of intensities of upregulated or downregulated proteins between the water treatment after 0 and 10 d. Fold changes had *P* values<0.05. In this column, ‘+’ means upregulated and ‘–’ means downregulated.

After 10 d of GA_4_ treatment, there were 38 differentially expressed spots, 32 of which were confidently identified according to the databases. After 10 d of water treatment, 15 protein spots were differentially expressed. Five protein spots were differentially expressed in both GA_4_ treatment and water treatment, and had similar expression trends (Tables 1 and 2). The Mal d1 homologue (spot 1) showed a decrease in expression, whereas the other four protein spots (spots 8, 23, 28, and 36) showed an increase in expression in both treatments (Tables 1 and 2). There were more proteins associated with energy metabolism and oxidation–reduction that were differentially expressed after the GA_4_ treatment compared with the water treatment. Therefore, proteins associated with energy metabolism and oxidation–reduction may play an important role in dormancy release after applying GA_4_.

### DGE profiling of specific genes in response to dormancy break by GA_4_

**Fig. 4.**
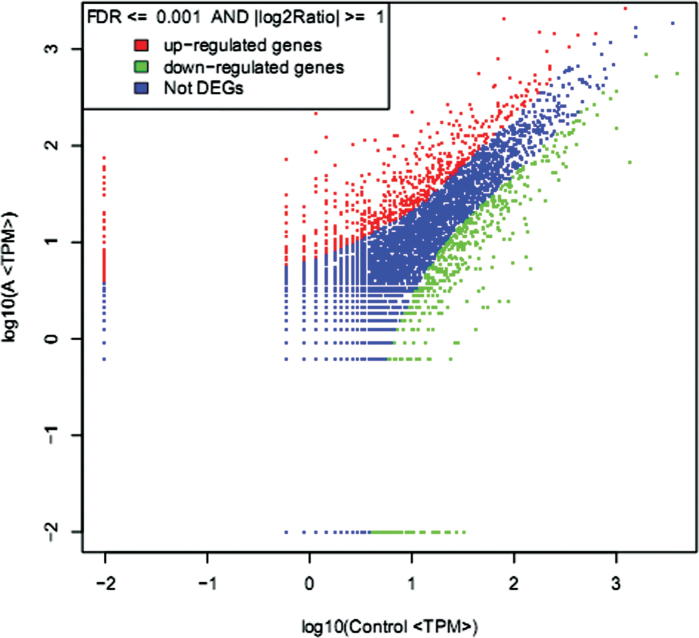
Comparison of transcripts expression between control and A libraries. The abundance of each gene was normalized as transcripts per million (TPM). The differential transcripts are shown in red and green, while blue indicates transcripts that were not differentially expressed (i.e. not differentially expressed genes). (This figure is available in colour at *JXB* online.)

To understand further the effect of GA_4_ on dormancy release in Japanese apricot, the regulation of gene expression was investigated using comparative DGE profiling analysis. After filtering dirty tags from the raw data, a total of 3 391 578 and 3 244 431 clean tags that corresponded to 163 411 and 168 839 distinct tags for the GA_4_ treatment on 30 December 2011 in the control and A libraries were obtained, respectively (Supplementary Table S4 at *JXB* online). There were 1176 differentially expressed genes, 668 and 508 of which were up- or downregulated after GA_4_ treatment (Fig. 4 and Supplementary Table S5 at *JXB* online). After treatment with GA_4_, the differentially expressed genes were categorized into three functional groups: molecular function, cellular component, and biological process (Fig. 3B). The significant enrichment categories according to molecular function were structural molecule activity (49) and oxidoreductase activity (139) (Fig. 3B). The genes were classified on the basis of cellular components into plastid (170), organelle part (174), intracellular organelle part (146), cytoplasmic part (272), cytoplasm (272), and macromolecular complex (103). On the basis of biological processes, the generation of precursor metabolites and energy (33) and carbon fixation (8) were the major categories. Consistent with the proteomic data, many genes belonging to oxidoreductase activity, generation of precursor metabolites, and energy and carbon fixation were differentially expressed (Fig. 3B). This analysis allowed the major biological functions of differentially expressed genes to be determined. Pathway-based analysis was undertaken to understand further the biological functions of these genes. Using pathway enrichment analysis it was possible to determine which metabolic and signal transduction pathways the differentially expressed genes were associated with. The pathways with the most unique sequences were ‘metabolic’ (218) and ‘ribosome’ (48) pathways (Supplementary Fig. S5 at *JXB* online). These analyses improve our understanding of the effect of GA_4_ on promoting dormancy release in Japanese apricot, and genomic manipulation of these genes might be important in being able to manipulate the state of dormancy.

To confirm the reliability of the Solexa/Illumina sequencing technology, the genes encoding the following 12 proteins were randomly selected for qRT-PCR assays as follows: β-galactosidase (ppa020752), histone superfamily protein (ppa013173), β-amylase 6 (ppa004334), tubulin β8 (ppa004884), glutathione *S*-transferase TAU 10 (ppa025728), aldehyde dehydrogenase 3I1 (ppa005609), ATPase, F1 complex, OSCP/δ subunit protein (ppa1027122), chitinase A (ppa026927), gibberellin-regulated family protein (ppa024899), actin-related protein 5 (ppa002045), dormancy-associated protein-like 1 (ppa013510), and late embryogenesis abundant protein (LEA) family protein (ppa011378). The results showed that the expression of 11 genes was consistent between the qRT-PCR and the DGE analyses, while that of the gene encoding the gibberellin-regulated family protein (ppa024899) was inconsistent (Supplementary Fig. S6A, B at *JXB* online).

### Correlation analysis of proteomics and transcriptomics

**Fig. 5.**
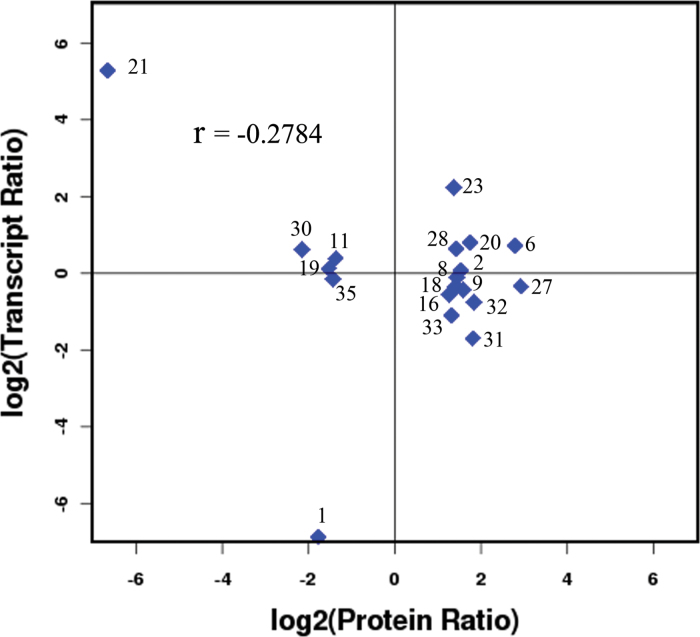
Correlation between the differentially expressed proteins and genes in the whole library. The *x*-axis shows the expression quantity of the differentially expressed proteins and the *y*-axis shows the expression quantity of genes in the whole library. (This figure is available in colour at *JXB* online.)

This study identified a correlative element between the proteins identified in the proteomics analysis and the genes quantified in the transcriptomics analysis, which showed that the number of relationships in protein analysis was 19, accounting for 65.52 and 0.16% of the 29 differentially expressed proteins, and both 29 differentially expressed proteins and 12 067 genes, respectively (Supplementary Fig. S7 and Supplementary Table S6 at *JXB* online). Among these 19 protein spots, there were 15 upregulated protein spots and four downregulated protein spots, and the expression trend of five proteins (spots 2, 6, 23, 28, and 35) was consistent with that of the corresponding genes after GA_4_ treatment, while the expression of the remaining 14 protein spots was inconsistent with that of the corresponding genes. We also conducted a correlation analysis between the differentially expressed proteins and genes in the whole library; the correlation coefficient was determined as –0.2784 (Fig. 5), which indicated a negative directional correlation between the mRNA and protein abundance ratios, although there was a relatively weak correlation. Principal component analysis of the whole library gene expression, differential expression genes, and differential expression proteins was also conducted in our study, which showed significance between the up-/downregulated genes/proteins and the expressed genes/proteins in the three data sets (Supplementary Fig. S8 at *JXB* online). In the DGE data, genes encoding manganese superoxide dismutase, peroxidase superfamily protein, copper/zinc-superoxide dismutase, and ascorbate peroxidase 1 were also differentially expressed after GA_4_ treatment. The proteins of spots 23 and 28 and the corresponding genes had a consistent increase in expression after GA_4_ treatment. However, the expression trends between proteins and the corresponding genes (spots 27 and 30) were inconsistent after GA_4_ treatment. The main focus of the study was the 42 differentially expressed proteins and related genes. Many genes in the DGE database, such as those encoding β-galactosidase 7 (ppa020752), β-1,3-glucanase 1 (ppa008126), gibberellin 2-oxidase 6 (ppa008310), cold-regulated 47 (ppa005514), histone superfamily protein (ppa013173), and dormancy-associated protein-like 1 (ppa013510), were also important in dormancy release of Japanese apricot after GA_4_ treatment. Furthermore, there were many other differentially expressed genes in the DGE database that have not yet been characterized by researchers.

## Discussion

### Proteins and genes associated with energy metabolism play an important role in dormancy release

**Fig. 6.**
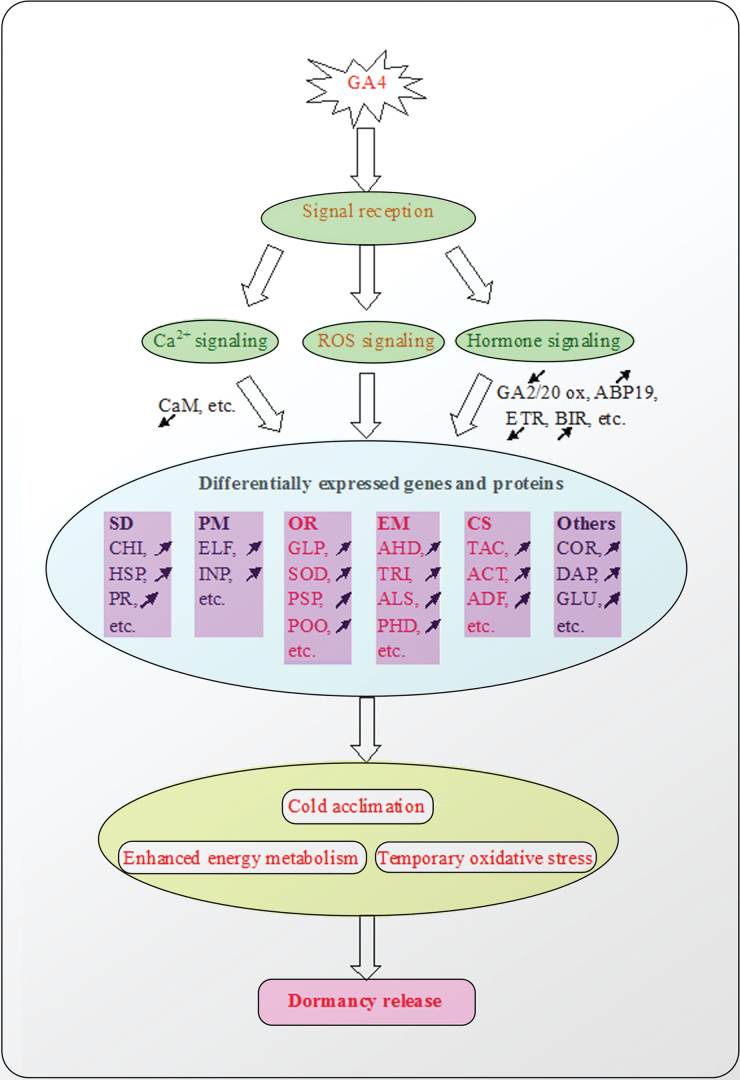
Molecular model of dormancy release in Japanese apricot treated with GA_4_. In this model, after GA_4_ treatment, signal reception, including Ca^2+^ signalling, ROS signalling, and hormone signalling modulate the expression of many kinds of genes and proteins, which include SD (stress and defence), PM (protein metabolism), OR (oxidation–reduction), EM (energy metabolism), CS (cell structure), and ‘others’, including signalling and transcription. These genes and proteins provide increased cold tolerance and enhanced energy metabolism to Japanese apricot, and/or cause it to enter a temporary oxidative stress state, which contributes to dormancy release. The black arrows indicate up-/downregulation of genes after GA_4_ treatment, and processes marked in red are more critical due to many genes or proteins being differentially expressed after GA_4_ treatment. Proteins and genes associated with SD, PM, OR, EM, and CS are mainly in the protein database, while those classified as ‘others’ are mainly in the DGE database. CHI, class IV chitinase; HSP, heat shock protein 60; PR, pathogenesis-related thaumatin superfamily protein; ELF, elongation factor Tu (ISS); INP, insulinase (peptidase family M16) protein; GLP, glutathione peroxidase 6; SOD, manganese superoxide dismutase and copper/zinc-superoxide dismutase; PSP, peroxidase superfamily protein; POO, polyphenol oxidase; AHD, alcohol dehydrogenase 1; TRI, triosephosphate isomerase, putative; ALS, aldolase superfamily protein; PHD, D-3-phosphoglycerate dehydrogenase; TAC, tubulin α-2 chain; ACT, actin 7; ADF, actin-depolymerizing factor; DAP, dormancy-associated protein-like 1; GLU, β-1,3-glucanase 1; ABP19, auxin-binding protein ABP19b; ETR, ethylene receptor; BIR, brassnosteroid insensitive 1- associated receptor kinase 1 precursor, putative. (This figure is available in colour at *JXB* online.)

The transition from dormancy to active bud growth is accompanied by numerous molecular and biochemical changes, including changes in carbohydrate metabolism (Maurel *et al.*, 2004). Many studies have suggested that bud meristems require sufficient energy from the underlying tissue to sustain bud growth at the time of dormancy release. Recently, several proteomic and transcriptional results have shown that energy metabolism is a prerequisite for leaf and flower development, including dormancy release (Bi *et al.*, 2011; Prassinos *et al.*, 2011; Zhuang *et al.*, 2012). In this study, nine proteins associated with dormancy release of Japanese apricot buds, following both water and GA_4_ treatment, were involved in energy metabolism, accounting for 18.4% of the differentially expressed proteins (49). The majority of these were proteins involved in glycolysis (triosephosphate isomerase, d-3-phosphoglycerate dehydrogenase, aldolase superfamily protein) and the tricarboxylic acid cycle (ribulose 1,5-bisphosphate carboxylase). In plants, the glycolysis/tricarboxylic acid cycle is the main biochemical pathway that provides plant mitochondria with pyruvate, supporting plant respiration and the biosynthesis of numerous essential metabolic compounds. Or *et al.* (2000) observed that alcohol dehydrogenase was involved in anaerobic respiration and was induced in response to respiratory stress in grape buds after hydrogen cyanamide treatment; they also found that respiratory interference, possibly a change in the AMP:ATP ratio, was involved in dormancy release. The results of this study have also demonstrated that energy metabolism is one of the most important factors associated with bud dormancy release. Compared with water treatment, many proteins associated with energy metabolism were differentially expressed. In the DGE database, many genes were also involved in energy metabolism. Therefore, GA_4_ stimulated the activity of genes and proteins involved in energy metabolism, which might lead to earlier dormancy break in the Japanese apricot bud (Fig. 6).

### Protein metabolism

Bi *et al.* (2011) suggested that glutamine synthetase probably plays an important role in cell proliferation and differentiation in the bud of *Pinus sylvestris* L. var. *mongolica litv*. In this study, spot 16 was identified as glutamine synthetase, the expression level of which increased 2.43-fold after GA_4_ treatment for 10 d compared with GA_4_ treatment at 0 d. Therefore, glutamine synthetase might increase cell proliferation and differentiation during GA_4_ treatment and subsequently play a specific role in breaking the dormancy of the Japanese apricot bud.

Elongation factor 1 (EF-1), a component of the protein synthesis machinery, is a prerequisite for maintaining rapid cell division and protein synthesis in tissues such as meristematic tissues or somatic embryos (Yang *et al.*, 2005). Pawłowski (2007) suggested that initiation and elongation factors might play a major role in beech seed dormancy release and be responsible for protein synthesis and cell division in the root meristem. However, the same author also considered that elongation factor EF-Tu was part of the plastid translational apparatus and probably took part in the build-up of the photosynthetic system during germination. In this study, elongation factor Tu (ISS) (spot 17) increased 2.65-fold when comparing the results of GA_4_ treatment after 10 and 0 d, which indicated that it might play an important role in breaking the dormancy of Japanese apricot buds after GA_4_ treatment.

### Oxidation–reduction processes involving many proteins and genes might be part of the mechanism that leads to bud break

Many researchers have suggested that oxidative stress is an important part of the process of dormancy release, and that the antioxidant defence system, including glutathione peroxidase, superoxide dismutase, ascorbate peroxidase, and peroxidase superfamily protein, play a pivotal role in dormancy release (Or *et al.*, 2000; Mazzitelli *et al.*, 2007; Halaly *et al.*, 2008; Prassinos *et al.*, 2011). In this study, many oxidation–reduction proteins, mainly comprising glutathione peroxidase 6 (spot 22), superoxide dismutase (spots 23 and 28), peroxidase superfamily protein (spot 27), and ascorbate peroxidase (spots 24, 25, 26, and 30) after GA_4_ treatment were differentially expressed. Spots 24, 25, and 30 were downregulated, but spot 26 was upregulated after 10 d of GA_4_ treatment. Two reasons might account for this: one is that different spots were identified as the same protein, corresponding either to post-translational modification of the same protein, alternative splicing, and the occurrence of multigene families, or to various isoforms; another was the subcellular localization of the different spot proteins. Prassinos *et al.* (2011) observed that peroxidase, which generates H_2_O_2_ by NADH oxidation, was upregulated in vegetative buds of peach, and Zhuang *et al.* (2012) observed that the decrease in APX I as a signalling molecule during dormancy release might regulate consecutive dormancy release and bud break in Japanese apricot. Many studies have indicated that bud dormancy release coincides with an upregulation of the antioxidant system (Mazzitelli *et al.*, 2007; Prassinos *et al.*, 2011). Consistent with this, most proteins associated with oxidation–reduction after GA_4_ treatment were upregulated during dormancy release, except for APX I. Halaly *et al.* (2008) suggested that a decrease in free-radical levels was required for the termination of dormancy and the induction of bud break. Perez and Lira (2005) observed a transient increase in H_2_O_2_ levels preceding the release of endodormancy in buds of grapevine treated with hydrogen cyanamide, a catalase inhibitor. Prassinos *et al.* (2011) suggested that the transient peak of H_2_O_2_ preceding dormancy release can act as a signalling molecule to trigger the transition from dormancy to bud break. Increasing evidence has shown that oxidative stress might play a role in endodormancy release in perennials (Or *et al.*, 2000; Arora *et al.*, 2003; Or, 2009). In this study, nine proteins associated with oxidation–reduction after GA_4_ treatment and three proteins after water treatment were differentially expressed. Spots 23 and 28 showed an increased expression trend following both GA_4_ and water treatment. In the DGE database, 139 genes were related to oxidoreductase activity (Fig. 3B). Therefore, we hypothesized that GA_4_ application might lead to the development of oxidative stress and to subsequent dormancy break earlier than in the water treatment (Fig. 6).

### Proteins and genes associated with cell structure

Importantly, three proteins associated with cell structure (spots 31, 32, and 33), were identified in this study. α-Tubulin (TUA), which constitutes the plant cytoskeleton, is an important component of microtubules (Goddard *et al.*, 1994). TUAs have been shown to be modulated by drought, abscisic acid and GA_3_ in an isoform-speciﬁc manner (Marino *et al.*, 2009; Hashim *et al.*, 2010). Proteomic analysis of *Arabidopsis* seeds showed that α-2,4-tubulin, a cytoskeleton component, appears to depend on the action of GA during germination. Paul *et al.* (2012) observed that all the *CsTUA* of the tea they studied exhibited upregulation during winter dormancy. Zhuang *et al.* (2012) showed that the tubulin α-2 chain had a high expression level during winter dormancy, and subsequently declined during dormancy release. In the present study, the tubulin α-2 chain (spot 31) was upregulated 3.58-fold after GA_4_ treatment. In contrast, there was no significant change after water treatment. Therefore, GA_4_ application might promote cell division and cell elongation, which contribute to dormancy break in Japanese apricot buds.

### Proteins and genes associated with signalling and transcription

Pang *et al.* (2007) suggested that calcium signalling is involved in the mechanism of bud dormancy release in grape. Calreticulin, a major Ca^2+^-sequestering protein, might play an important role in signal transduction cascades by affecting Ca^2+^ homeostasis during developmental regulation (Shen *et al.*, 2003). In beech seeds, calreticulin, is an important component of the GA signalling pathway and might play an important role in the hormone signal transduction cascades that lead to dormancy breaking and germination (Shen *et al.*, 2003; Pawłowski, 2007). In this study, both calreticulin 1a (spot 35) and its corresponding gene were downregulated after GA4 treatment. Spot 35 had a differential expression after GA_4_ treatment and showed no significant change after water treatment, and might thus play an important role in the dormancy release of Japanese apricot.

In our study, the expression trends between proteins and the corresponding genes (spots 27 and 30) were inconsistent after GA_4_ treatment. Many reasons can lead to this phenomenon: first, the lack of changes in protein abundance in these genes might be due to post-translational (down)regulation of the protein activity to avoid a *de novo* cycle of synthesis after the stress is relieved; secondly, the time course of the decline differs between mRNAs and proteins and does not allow monitoring of changes at the protein level at the observed time point; thirdly, transcript regulation may be ‘unwanted’ but unavoidable for a subset of mRNAs, due to the broad action of *trans*-acting factors, while protein abundance remains stable, being controlled by post-translational mechanisms.

### Conclusions

Exogenous applications of GA_4_ induced dormancy break, and many genes associated with GA metabolism, such as *GA20OX* and *GA2OX*, were found to have a significant alteration in expression. Globally, our results suggested an important role for proteins and genes involved with energy metabolism and oxidation–reduction after GA_4_ treatment in the coordination of dormancy break in Japanese apricot flower buds at the proteomic and transcriptomic levels. The results of this study provide a global picture of protein accumulation and changes in gene expression in the dormancy of Japanese apricot flower buds following GA_4_ treatment.

## Supplementary data

Supplementary data are available at *JXB* online.

Supplementary Fig. S1. Morphology changes before and after the dormancy break in Japanese apricot.

Supplementary Fig. S2. Experimental design associated with dormancy release in Japanese apricot.

Supplementary Fig. S3. Experimental process of DGE profiles.

Supplementary Fig. S4. Images of the original gels and the matchsets appearing in Fig. 2.

Supplementary Fig. S5. Histogram showing pathway enrichment analysis for differentially expressed genes in Japanese apricot after GA_4_ treatment (10 d).

Supplementary Fig. S6. Real-time qPCR validations of tag-mapped genes.

Supplementary Fig. S7. Correlation between the differentially expressed proteins and genes in the whole library.

Supplementary Fig. S8. Principal component analysis of the whole library genes (A), differentially expressed genes (B) and differentially expressed proteins (C).

Supplementary Table S1. Specific primers used in relative qRT-PCR.

Supplementary Table S2. The matched peptide sequences and corresponding *m*/*z* ratio of the 42 identified proteins in our study.

Supplementary Table S3. Three replicates data sets of protein spot expression value in the control, G10 and W10 in our study.

Supplementary Table S4. Summary statistics of DGE tags in Japanese apricot after GA_4_ treatment in the control and A libraries.

Supplementary Table S5. The up- and downregulated genes in Japanese apricot after GA_4_ treatment (10 d).

Supplementary Table S6. **C**orrelation between the differentially expressed proteins and genes in the whole library.

Supplementary Data

## Abbreviations:

2-DE: two-dimensional gel electrophoresis
DGE: digital gene expression
DTT: dithiothreitol
GA: gibberellin
MALDI-TOF/TOF: matrix-assisted laser desorption/ionization time-of-flight/time-of-flight
MW: molecular weight
MS: mass spectrometry
pI: isoelectric point
qRT-PCR: quantitative reverse transcription-PCR.

## References

[CIT0001] Arora R Rowland LJ Tanino K 2003 Induction and release of bud dormancy in woody perennials: a science comes of age. HortScience 38, 911–921

[CIT0002] Audic S Claverie JM 1997 The significance of digital gene expression profiles. Genome Research 7, 986–995 9331369 10.1101/gr.7.10.986

[CIT0003] Baggiolini M 1952 Stade repères du pecher. Revue Romande d’Agriculture Viticulture et Arboriculture 4, 29–35

[CIT0004] Barros PM Goncalves N Saibo NJM Oliveria MM 2012 Cold acclimation and floral development in almond bud break: insights into the regulatory pathways. Journal of Experimental Botany 63, 4585–4596 22685307 10.1093/jxb/ers144

[CIT0005] Bhushan D Pandey A Choudhary MK Datta A Chakraborty S Chakraborty N 2007 Comparative proteomics analysis of differentially expressed proteins in chickpea extracellular matrix during dehydration stress. Molecular and Cellular Proteomics 6, 1868–1884 17686759 10.1074/mcp.M700015-MCP200

[CIT0006] Bi YD Wei ZG Shen Z Lu TC Cheng YX Wang BC Yang CP 2011 Comparative temporal analyses of the *Pinus sylvestris* L. var. *mongolica litv*. apical bud proteome from dormancy to growth. Molecular Biology Reports 38, 721–729 20373030 10.1007/s11033-010-0159-2

[CIT0007] Bradford MM 1976 A rapid and sensitive method for the quantification of microgram quantities of protein utilizing the principle of protein-dye binding. Analytical Biochemistry 72, 248–254 942051 10.1016/0003-2697(76)90527-3

[CIT0008] Campbell RK Sugano AI 1975 Phenology of bud break in Douglas-fir related to provenance, photoperiod, chilling and flushing temperature. Botanical Gazette 136, 290–298

[CIT0009] Chu MY 1999 China fruit records-mei . Beijing: China Forestry Press

[CIT0010] de Oliveira OR Peressuti RA Skalitz R Antunes MC Biasi LA Zanette F 2008 Dormancy broken of ‘Hosui’ pear trees with mineral oil in two training systems. Revista Brasileira de Fruticultura 30, 409–413

[CIT0011] Faust M Liu DH Merle MM Stutte GW 1991 Bound versus free water in dormant apple buds-a theory for endodormancy. HortScience 26, 887–890

[CIT0012] Goddard RH Wick SM Silﬂow CD Snustad DP 1994 Microtubule components of the plant cytoskeleton. Plant Physiology 104, 1–6 12232055 10.1104/pp.104.1.1 PMC159156

[CIT0013] Halaly T Pang XQ Batikoff T Crane O Keren A Venkateswari J Ogrodovitch A Sadka A Lavee S Or E 2008 Similar mechanisms might be triggered by alternative external stimuli that induce dormancy release in grape buds. Planta 228, 79–88 18324412 10.1007/s00425-008-0720-6

[CIT0014] Hashim S Hachinohe M Matsumoto H 2010 Cloning and expression analysis of α-tubulin genes in water foxtail (*Alopecurus aequalis*). Weed Science 58, 89–95 10.1002/ps.2284 21972152

[CIT0015] Hedley PE Russell JR Jorgensen L Gordon S Morris JA Hackett CA Cardle L Brennan R 2010 Candidate genes associated with bud dormancy release in blackcurrant (*Ribes nigrum* L.). BMC Plant Biology 10, 202–215 20840772 10.1186/1471-2229-10-202 PMC2956551

[CIT0016] Hoffman DE 2011 Changes in the transcriptome and metabolome during the initiation of growth cessation in hybrid aspens . PhD thesis, Swedish University of Agricultural Sciences.

[CIT0017] Lang GA Early JD Martin GC Darnell RL 1987 Endo-para and eco-dormancy: physiological terminology and classification for dormancy research. HortScience 22, 371–377

[CIT0018] Leida C Terol J Martí G Agustí M Llácer G Badenes ML Ríos G 2010 Identification of genes associated with bud dormancy release in *Prunus persica* by suppression subtractive hybridization. Tree Physiology 30, 655–666 20231169 10.1093/treephys/tpq008

[CIT0019] Li L Wang H Tan Y Wang Y Chen XD Li DM Gao DS 2011 Construction of the suppression subtractive hybridization library and analysis of related genes of floral buds in *Prunus persica* during dormancy-releasing. Acta Horticulturae Sinica 38, 2273–2280

[CIT0020] Looney NE 1997 Hormones and horticulture. HortScience 32, 1014–1018

[CIT0021] Marino R Ponnaiah M Krajewski P Frova C Gianfranceschi L Pè ME Sari-Gorla M 2009 Addressing drought tolerance in maize by transcriptional profiling and mapping. Molecular Genetics and Genomics 281, 163–179 19018570 10.1007/s00438-008-0401-y

[CIT0022] Mathiason K He D Grimplet J Venkateswari J Galbraith DW Or E Fennell A 2009 Transcript profiling in *Vitis riparia* during chilling requirement fulfillment reveals coordination of gene expression patterns with optimized bud break. Functional & Integrative Genomics 9, 81–96 18633655 10.1007/s10142-008-0090-y

[CIT0023] Maurel K Sakr S Gerbe F Guilliot A Bonhomme M Rageau R Pétel G 2004 Sorbitol uptake is regulated by glucose through the hexokinase pathway in vegetative peach-tree buds. Journal of Experimental Botany 55, 879–888 14990620 10.1093/jxb/erh087

[CIT0024] Mazzitelli L Hancock RD Haupt S 2007 Co-ordinated gene expression during phases of dormancy release in raspberry (*Rubus idaeus* L.) buds. Journal of Experimental Botany 58, 1035–1045 17244630 10.1093/jxb/erl266

[CIT0025] Mølmann JA Asante DKA Jensen JB Krane MN Ernstsen A Junttila O Olsen JE 2005 Low night temperature and inhibition of gibberellin biosynthesis override phytochrome action and induce bud set and cold acclimation, but not dormancy in *PHYA* overexpressors and wild-type of hydrid aspen. Plant, Cell & Environment 28, 1579–1588

[CIT0026] Morrissy AS Morin RD Delaney A Zeng T McDonald H Jones S Zhao Y Hirst M Marra MA 2009 Next-generation tag sequencing for cancer gene expression profiling. Genome Research 19, 1825–1835 19541910 10.1101/gr.094482.109 PMC2765282

[CIT0027] Or E Vilozny I Eyal Y Ogrodovitch A 2000 The transduction of the signal for grape bud dormancy breaking induced by hydrogen cyanamide may involve the *SNF-like* protein kinase GDBRPK. Plant Molecular Biology 43, 483–494 11052200 10.1023/a:1006450516982

[CIT0028] Or E 2009 Grape bud dormancy release-the molecular aspect. In: Roubelakis-Angelakis KA, ed. Grapevine molecular physiology & biotechnology . Berlin: Springer, 1–29

[CIT0029] Pang X Halaly T Crane O Keilin T Keren A Ogrodovitch A Galbraith D Or E 2007 Involvement of calcium signalling in dormancy release of grape buds. Journal of Experimental Botany 58, 3249–3202 17977848 10.1093/jxb/erm172

[CIT0030] Paul A Lal L Ahuja PS Kumar S 2012 *Alpha-tubulin* (*CsTUA*) up-regulated during winter dormancy is a low temperature inducible gene in tea [*Camellia sinensis* (L.) O. Kuntze]. Molecular Biology Reports 39, 3485–3490 21725638 10.1007/s11033-011-1121-7

[CIT0031] Pavy N Boyle B Nelson C 2008 Identification of conserved core xylem gene sets: conifer cDNA microarray development, transcript profiling and computational analyses. New Phytologist 180, 766–786 18811621 10.1111/j.1469-8137.2008.02615.x

[CIT0032] Pawłowski TA 2007 Proteomics of European beech (*Fagus sylvatica* L.) seed dormancy breaking: influence of abscisic and gibberellic acids. Proteomics 7, 2246–2257 17533642 10.1002/pmic.200600912

[CIT0033] Perez FJ Lira W 2005 Possible role of catalase in post-dormancy bud break in grapevines. Journal of Plant Physiology 162, 301–308 15832682 10.1016/j.jplph.2004.07.011

[CIT0034] Prassinos C Rigas S Kizis D Vlahou A Hatzopoulos P 2011 Subtle proteome differences identified between post-dormant vegetative and flower peach buds. Journal of Proteomics 74, 607–619 21315198 10.1016/j.jprot.2011.01.018

[CIT0035] Rinne PLH Welling A Vahala J Ripel L Ruonala R Kangasjärvi J van der Schoot C 2011 Chilling of dormant buds hyperinduces *FLOWERING LOCUS T* and recruits GA-inducible 1, 3-β-glucanases to reopen signal conduits and release dormancy in *Populus* . Plant Cell 23, 130–146 21282527 10.1105/tpc.110.081307 PMC3051240

[CIT0036] Sabry GH El-Helw HA Abd El-Rahman AS 2011 A study on using jasmine oil as a breaking bud dormancy for flame seedless grapevines. Report and Opinion 3, 48–56

[CIT0037] Sagredo KX Theron KI Cook NC 2005 Effect of mineral oil and hydrogen cyanamide concentration on dormancy breaking in ‘Golden Delicious’ apple trees. South African Journal of Plant and Soil 22, 251–256

[CIT0038] Saure MC 1985 Dormancy release in deciduous fruit trees. Horicultura Review 7, 239–299

[CIT0039] Schrader J Moyle R Bhalerao R Hertzberg M Lundeberg J Nilsson P Bhalerao RP 2004 Cambial meristem dormancy in trees involves extensive remodelling of the transcriptome. The Plant Journal 40, 173–187 15447645 10.1111/j.1365-313X.2004.02199.x

[CIT0040] Shen S Sharma A Komatsu S 2003 Characterisation of proteins responsive to gibberellin in the leaf-sheath of rice (*Oryza sativa* L.) seedling using proteome analysis. Biological and Pharmaceutical Bulletin 26, 129–136 12576669 10.1248/bpb.26.129

[CIT0041] Sugiura T Kuroda H Sugiura H 2007 Influence of the current state of global warming on fruit tree growth in Japan. Horticultural Research 6, 257–263

[CIT0042] ’t Hoen PAC Ariyurek Y Thygesen HH Vreugdenhil E Vossen RHAM de Menezes RX Boer JM van Ommen GJB den Dunnen 2008 Deep sequencing-based expression analysis shows major advances in robustness, resolution and inter-lab portability over five microarray platforms. Nucleic Acids Research 36, e141 18927111 10.1093/nar/gkn705 PMC2588528

[CIT0043] Tong Z Gao Z Wang F Zhou J Zhang Z 2009 Selection of reliable reference genes for gene expression studies in peach using real-time PCR. BMC Molecular Biology 10, 71. 10.1186/1471-2199-10-71 PMC3224724 19619301

[CIT0044] Yang GX Inoue A Takasaki H Kaku H Akao S Komatsu S 2005 A proteomic approach to analyse auxin- and zinc-responsive protein in rice. Journal of Proteome Research 4, 456–463 15822922 10.1021/pr049801h

[CIT0045] Zawaski C Kadmiel M Pickens J Ma C Strauss S Busov V 2011 Repression of gibberellin biosynthesis or signalling produces striking alterations in poplar growth, morphology, and flowering. Planta 234, 1285–1298 21792553 10.1007/s00425-011-1485-x

[CIT0046] Zhuang WB Gao ZH Zhang Z Shi T Shao J 2011 Optimisation of two-dimensional electrophoresis conditions for floral buds proteome analysis of Japanese apricot. Journal of Nanjing Agricultural University 34, 47–52

[CIT0047] Zhuang WB Shi T Gao ZH Zhang Z Zhang JY 2012 Differential expression of proteins associated with seasonal bud dormancy at four critical stages in Japanese apricot. Plant Biology 15, 233–242 22672637 10.1111/j.1438-8677.2012.00589.x

