# Supplementary material for: Comparative proteomic and transcriptomic approaches to address the active role of GA4 in Japanese apricot flower bud dormancy release
Source: J Exp Bot. 2013 Sep 7;64(16):4953–66. doi: 10.1093/jxb/ert284 (PMC3830480; doi:10.1093/jxb/ert284)
Supplement: Supplementary Data [file supp_64_16_4953__index.html]

Comparative proteomic and transcriptomic approaches to address the active role of GA4 in Japanese apricot flower bud dormancy release — Comparative proteomic and transcriptomic approaches to address the active role of GA4 in Japanese apricot flower bud dormancy release — Supplementary Data 

# Comparative proteomic and transcriptomic approaches to address the active role of GA4 in Japanese apricot flower bud dormancy release

## Supplementary Data

Data files

**Files in this Data Supplement:**

- Supplementary Data - Supplementary Data
