# Supplementary material for: Comparative proteomic and transcriptomic approaches to address the active role of GA4 in Japanese apricot flower bud dormancy release
Source: J Exp Bot. 2013 Sep 7;64(16):4953–66. doi: 10.1093/jxb/ert284 (PMC3830480; doi:10.1093/jxb/ert284)

Comparative proteomic and transcriptomic approaches to address the active role of GA_4 in Japanese apricot flower buds dormancy release

Weibing Zhuang, Zhihong Gao, Liangju Wang, Wenjun Zhong, Zhaojun Ni, Zhen Zhang

Supplementary material

Supplementary Table S1 Specific primers used in relative quantitative real-time RT-PCR.

Gene name	Accession	Forward primer Sequence (5'to 3')	Reverse primer Sequence (5' to 3')
beta-galactosidase	ppa020752	ACGAAGACTTTTGCTATCCG	AAATCATACTGGCGACGAAC
histone superfamily protein	ppa013173	GCTCCCATTTCAGCGTTTG	ATGATGGTGACCCTCTTGG
beta-amylase 6	ppa004334	GCTCAGGTATCCCTCTTAT	TCAGGAGCGTCATTGTATT
tubulin beta 8	ppa004884	GTCAATCTGGTGCTGGAAAC	ATACACTCGTCTGCGTTCT
glutathione S-transferase TAU 10	ppa025728	GCAAGCCAATCGCAGAATC	CACCACAATACCCAGAAAGC
aldehyde dehydrogenase 3I1	ppa005609	AACGAGGCACTGAAAGAAT	TCTTATCCCACTTCTGCTCT
ATPase, F1 complex, OSCP/delta subunit protein	ppa1027122	AACTGGGTATGCCGCTGCTC	CCAAATGCCTATTGAATCTCCCT
chitinase A	ppa026927	TGGACGGCATAGATTTCAACAT	TGTAGTCAGACAACCTCCT
gibberellin-regulated family protein	ppa024899	CTTGGCTCCTCTTTGATGG	CTTGAACTCCTGCTACTGC
actin-related protein 5	ppa002045	TATCTGTCCTGGATTTACC	AACAGTGCTCCATCTTCAGGT
dormancy-associated protein-like 1	ppa013510	GATGACATTGTTGCTGGAC	TTAGCGATGCTTGCTCCTGGTC
late embryogenesis abundant protein (LEA) family protein	ppa011378	CAGTATCAGTAGCCTCGTT	CCCAATGTTCTGTTCCCTC

Supplementary Table S2 The matched peptide sequences and corresponding m/z ratio of the 42 identified proteins in our study.

[a]Spot No	[b]NP	[c]Matched peptide sequences	corresponding m/z ratio
1	4	K.ITFGEGSQYGYVK.H	1448.7163
		K.KITFGEGSQYGYVK.H	1576.8153
		K.HSEILEGDGGPGTIKK.I	1637.8595
		M.GVFTYESEFTSEIPPPR.L	1955.9763
2	3	K.AIDERSEQIR.S	1216.6252
		R.NVVIEQSFGAPK.V	1288.6797
		R.GYISPYFITNQK.N	1430.7239
3	4	K.SVAAGMNAMDLRR.G	1391.6783
		R.GYISPYFITNPK.N	1399.7144
		K.TLYNELEVVEGMKLDR.G	1908.9644
		K.QVANATNDAAGDGTTCATVLTR.A	2207.0308
5	2	K.RPDLVQAR.I	954.5444
		R.AINGAVECDGKRPDLVQAR.I	2069.0476
6	5	A.AKITFTNK.C	922.5299
		K.GSDGSVIACK.S	993.4805
		K.ASTCPADVNAVCPAELQVK.G	2087.0239
		K.IFKDQCPQAYSYAYDDK.S	2111.9314
		K.SACLALNQPQYCCTPPNDKPETCPPTEYSK.I	3560.5208
8	8	K.GQHPLFPR.I	951.5095
		K.GTFYGNYKPR.S	1174.5764
		K.THPVNFLNER.T	1226.6185
		K.AFDLMLSGQSIR.C	1337.6759
		R.IIGVDLNSNRFEEAK.K	1714.8999
		R.IIGVDLNSNRFEEAKK.F	1842.991
		K.KGSSVAIFGLGAVGLAAAEGAR.V	2002.094
		R.AAVAWEAGKPLSIEEVEVAPPQKEEVR.L	2932.5415
9	6	K.TASGKPFVDVLK.E	1261.7113
		K.ASPEVIAEYTVR.A	1334.6919
		K.KASPEVIAEYTVR.A	1476.7981
		K.GILAADESTGTIGKR.F	1488.7963
		R.FASINVENVESNRR.A	1634.8182
		K.IGPNEPSQLSINENANGLAR.Y	2094.0491
10	2	K.VKDGVPHLTK.V	1093.6373
		R.GGVIDEDALVR.A	1143.6039
11	7	R.AMLENILR.A	959.5358
		K.YVPYVADSKR.A	1197.6284
		K.VVVLWTANTER.Y	1287.7068
		R.VGSFNGEEIYAPFK.S	1557.7574
		MFIESFKVESPNVK.Y	1654.8512

		K.VQQANYFGSLTQASSIR.V	1869.9457
		K.DKVQQANYFGSLTQASSIR.V	2113.0713
12	3	K.FFVGGNWK.C	954.4681
		K.VIACVGETLEQR.E	1374.6797
		K.DSLKPDFHVAAQNCWVK.K	2014.9623
13	5	R.ALRLEDLR.I	985.5793
		K.TFQGPPHGIQVER.D	1465.7632
		K.DFQGPPHGIQVER.D	1479.7694
		K.YGRPLLGCTIKPK.L	1502.8491
		R.LTYYTPDYETKDTDILAAFR.V	2396.1619
15	4	R.QKNHGMHFR.V	1154.5646
		R.MSGGDHIHAGTV	1465.7179
		R.MSGGDHIHAGTVVGKLEGER.E	2050.0146
		R.EITLGFVDLLRDDFVEQDR.S	2280.2031
16	2	R.LTGKHETADINTFK.W	1574.8241
		K.HKEHIAAYGEGNER.R	1610.7733
17	2	K.EIAFDAIDKAPEEK.A	1575.798
		R.HYAHVDCPGHADYVK.N	1768.7843
18	7	R.QVLTYGER.K	965.5047
		R.KPVEHFLK.T	997.5815
		K.AAVLMNLESR.M	1103.5939
		R.EVEAIGGHIGASASR.E	1453.7429
		K.TYVPQMVELLVDAVR.N	1732.9276
		R.NPAFLEWEVNEELKK.V	1845.9386
		R.LNGSVLEDFVAENYTAR.R	1897.9318
19	8	R.VSYDLANNR.V	1051.5182
		R.LQTEAYNSLR.D	1194.6116
		R.VETFYYVGLK.G	1218.6416
		K.ATSFSYCLVNR.D	1317.6285
		R.VQVPTVSFLFADGK.S	1507.8143
		K.DSRVETFYYVGLK.G	1576.8075
		E.DLSTPVVSGVSQGSGEYFTR.I	2086.0222
		R.DLPSASGFALFDTCYDLSSR.S	2294.0381
20	5	K.TVFASATER.D	981.4905
		K.HLIYQHQHNK.Q	1317.6709
		K.GCVFTYDAVGSYER.V	1623.6991
		K.LADKAVLASSGFQADVK.A	1719.9153
		R.VGYSSQGSGSTLIIPFLDNQLK.S	2324.1978
21	4	K.LGEIDQVTQR.R	1146.5801
		R.FLCGSVGALVENGKR.L	1606.8414
		R.LLALDGSVLPVVFER.A	1627.9449
		R.LLALDGSVLPVVFERA	1698.9852

22	7	K.SIHDFTVK.D	946.5182
		K.GKDVDLSIYK.G	1137.6353
		K.SIHDFTVKDAK.G	1260.6759
		R.YAPTTTPLSIEK.D	1306.7079
		K.FLVDKEGNVVDR.Y	1390.7523
		R.YAPTTTPLSIEKDVKK.L	1776.9961
		R.FKAEYPIFDKVDVNGDNAAPIYK.F	2601.3875
23	6	K.ALEQLHDAIAK.G	1208.6426
		K.YASEVYEKESP	1301.5703
		K.HHQTYVTNYNK.A	1404.6464
		K.FNGGGHVNHSIFWK.N	1599.7581
		K.KLVVETTANQDPLVTK.G	1755.9645
		K.ALEQLHDAIAKGDAAAVVK.L	1920.0295
24	6	R.EDKPEPPPEGR.L	1250.5913
		K.GNDHLRDVFGK.T	1257.6204
		R.IAWHSAGTYDSR.T	1335.618
		K.HAAEQSHGANAGLDIAVR.L	1816.8868
		K.YAADEDAFFADYAAAHQR.L	2031.8658
		K.TMGLSDQDIVALSGGHTLGR.C	2043.9987
25	6	R.EDKPEPPPEGR.L	1250.6213
		K.GNDHLRDVFGK.T	1257.6552
		R.IAWHSAGTYDSR.T	1335.6538
		K.HAAEQSHGANAGLDIAVR.L	1816.9325
		K.YAADEDAFFADYAAAHQR.L	2031.9215
		K.TMGLSDQDIVALSGGHTLGR.C	2044.0458
26	7	K.ACAPLMLR.I	947.4691
		K.GNDHLRDVFGK.T	1257.6155
		R.IAWHSAGTYDSR.T	1335.6106
		M.GKSYPTVSEEYK.K	1515.7434
		K.HAAEQSHGANAGLDIAVR.L	1816.8796
		K.YAADEDAFFADYAAAHQR.L	2031.8593
		K.TMGLSDQDIVALSGGHTLGR.C	2043.9905
27	3	R.VVSCSDITALAAR.D	1362.6897
		K.VFKEDIGQAAGLLR.L	1516.8337
		R.DSVFLSGGPDYDVPLGR.K	1793.8508
28	2	K.NLTHGAPEDEIR.H	1351.6661
		R.HAGDLGNIVANADGVAEATIVDNQIPLTGPNAV IGR.A	3552.9392
29	4	K.VRDSLDTTK.L	1034.5638
		K.KAQDEVLVISGIK.Y	1399.828
		K.ATVSSADLTTNFPATLTDTITVEVAR.T	2694.457
		R.KATVSSADLTTNFPATLTDTITVEVAR.T	2822.5479
30	7	K.ACAPLMLR.I	945.5106

		R.EDKPEPPPEGR.L	1250.6094
		K.GNDHLRDVFGK.T	1257.6443
		R.IAWHSAGTYDSR.T	1335.6427
		K.HAAEQSHGANAGLDIAVR.L	1816.9215
		K.YAADEDAFFADYAAAHQR.L	2031.9071
		K.TMGLSDQDIVALSGGHTLGR.C	2044.0356
31	8	K.EIVDLCLDR.I	1132.5597
		R.LVSQVISSLTASLR.F	1473.8507
		R.SLDIERPTYTNLNR.L	1691.8628
		R.AIFVDLEPTVIDEVR.T	1715.9087
		R.AIFVDLEPTVIDEVR.T	1729.9193
		K.TVGRGDDAFNTFFSETGAGK.H	2076.9524
		R.QLFHPEQLISGKEDAANNFAR.G	2368.1829
		R.QLFHPEQLISGKEDAANNFAR.G	2385.1914
32	7	R.AVFPSIVGRPR.H	1198.7482
		K.GEYDESGPSIVHR.K	1445.6742
		K.NYELPDGQVITIGAER.F	1774.9039
		R.VAPEEHPVLLTEAPLNPK.A	1954.0725
		K.DLYGNIVLSGGSTMFPGIADR.M	2183.0947
		K.SSSSVEKNYELPDGQVITIGAER.F	2479.2507
		R.TTGIVLDSGDGVSHTVPIYEGYALPHAILR.L	3151.699
33	2	R.SIVFKIEEK.Q	1092.6321
		R.IFFIAWSPDTSR.V	1439.7449
35	6	A.KVFFEER.F	954.5153
		R.FEDGWDKR.W	1052.4905
		R.WVTSEWKK.E	1063.5643
		K.YVGIELWQVK.S	1266.6836
		K.HEQKLDCGGGYIK.L	1504.7363
		K.SGTLFDNILITDEPEYAK.Q	2026.0276
36	6	K.YLPHSEQK.S	1001.4897
		R.RQAQLKPETAPK.L	1366.7649
		K.AIITHQLQSLKR.E	1393.8146
		K.LQAALPGLPQELIK.E	1490.8678
		K.EIGLTNVYVAQLLR.R	1576.8484
		R.SYDPNLIQDPTIYR.L	1694.8157
38	8	K.STSHYHTK.G	972.4933
		K.AFVLDADNLVPK.I	1301.7198
		K.ITFGEGSQYGYVK.H	1448.7163
		K.HSEILEGDGGPGTIK.K	1509.7646
		K.KITFGEGSQYGYVK.H	1576.8123
		K.HSEILEGDGGPGTIKK.I	1637.8595
		M.GVFTYESEFTSEIPPPR.L	1955.9546

		M.GVFTYESEFTSEIPPPR.L	1969.9675
39	6	R.TIERPEYK.Y	1035.5544
		R.QPGPIPGLNTK.I	1121.6323
		K.GGNVTREPGPVK.G	1210.6584
		K.ITSFLDPDGWK.T	1278.6406
		K.ITRQPGPIPGLNTK.I	1491.8674
		K.SAEVVNLVTQELGGK.I	1543.8413
40	7	K.ITITNDKGR.L	1017.5773
		K.NALENYAYNMR.N	1358.6285
		K.NALENYAYNMR.N	1374.6219
		K.STVHDVVLVGGSTR.I	1426.782
		K.MDKSTVHDVVLVGGSTR.I	1800.9454
		K.VQQLLQDFFNGKELCK.S	1967.0206
		K.EQVFSTYSDNQPGVLIQVYEGER.T	2658.3003
41	5	K.HLNITLTR.S	967.5748
		R.EAELHAQKDQER.K	1453.7051
		R.SKFESLVNHLIER.T	1571.8561
		R.QAVTNPTNTVFGTKR.L	1633.8667
		K.GVNPDEAVAMGAAIQGGILR.G	1939.0106
42	5	R.ALRLEDLR.I	985.5885
		K.DTDILAAFR.M	1021.5345
		K.TFQGPPHGIQVER.D	1465.7737
		K.LTYYTPDYETKDTDILAAFR.M	2396.1758
		R.MTPQPGVPPPEAGAAVAAESSTGTWTTVWTDG LTSLDR.Y	3854.9519
43	8	K.IGLFGGAGVGK.T	975.5593
		K.THDFLPIHR.E	1135.6067
		K.VVDLLAPYQR.G	1173.6659
		K.AHGGFSVFAGVGER.T	1390.6832
		R.VGLTGLTVAEHFR.D	1399.7695
		K.VLNTGSPITVPVGR.A	1409.8174
		R.FTQANSEVSALLGR.I	1492.7762
		R.IPSAVGYQPTLATDLGGLQER.I	2186.1545
44	6	K.DPGQNPFSFQIGK.G	1448.7648
		K.GWDEGVLQMQVGEVAR.L	1773.8798
		K.GWDEGVLQMQVGEVAR.L	1789.875
		K.GAVIKGWDEGVLQMQVGEVAR.L	2242.1946
		K.GAVIKGWDEGVLQMQVGEVAR.L	2258.1819
		R.EVVSAGTGPKPTVGQKVTVHCTGYGK.N	2657.3752
45	5	K.AGINVIQIDEAALR.E	1482.845
		K.YGAGIGPGVYDIHSPR.I	1658.8521
		K.SEQAFYLDWAVHSFR.I	1855.8909
		R.KSEQAFYLDWAVHSFR.I	1983.9888

		R.EGVKYGAGIGPGVYDIHSPR.I	2072.0857
46	8	K.AFFDSTGGLWR.T	1256.6001
		K.GGSPYGAGTYAGDGTR.Q	1486.644
		R.QPSELELQQAFHQGK.Y	1722.8302
		R.QPSELELQQAFHQGK.Y	1739.8549
		K.VYIVYYSMYGHVEK.L	1750.8378
		K.VYIVYYSMYGHVEK.L	1766.8293
		K.SEVPIISPNDLSEADGLLFGFPTR.F	2574.2896
		K.SEVPIISPNDLSEADGLLFGFPTR.F	2588.301
47	5	M.ASAEIEFR.C	964.4606
		K.IINDRETGR.S	1073.5573
		R.GFGFVTFSNEK.A	1232.5844
		R.AFSPFGEIIESK.I	1324.6638
		R.CFVGGLAWATDNDALER.A	1894.8655

[a] Numbering corresponds to the 2–DE gel in Fig. 2.

[b] The total number of peptides identified.

[c] The sequences of matched peptides identified.

Supplementary Table S3 Three replicates data sets of protein spot expression value in control, G10 and W10 in our study.

[a]Spot No	control (1)	control (2)	control (3)	control (average)	G10 (1)	G10 (2)	G10 (3)	G10 (average)	W10 (1)	W10 (2)	W10 (3)	W10 (average)
1	8.31	8.31	8.31	8.31	2.44	2.46	2.45	2.45	3.30	3.28	3.29	3.29
2	0.65	0.65	0.65	0.65	1.89	1.90	1.91	1.90	1.11	1.11	1.11	1.11
3	1.35	1.35	1.35	1.35	3.47	3.49	3.54	3.50	1.43	1.46	1.40	1.43
4	0.31	0.32	0.27	0.30	2.80	2.80	2.80	2.80	0.36	0.36	0.36	0.36
5	0.32	0.30	0.28	0.30	5.80	6.30	6.20	6.10	0.27	0.26	0.31	0.28
6	1.80	1.80	1.80	1.80	12.60	12.60	12.60	12.60	2.23	2.23	2.23	2.23
7	7.95	7.93	7.97	7.95	1.90	1.90	1.90	1.90	5.67	5.67	5.67	5.67
8	5.48	5.43	5.44	5.45	14.99	14.99	14.99	14.99	14.30	14.31	14.23	14.28
9	2.64	2.66	2.65	2.65	8.05	8.05	8.05	8.05	5.00	5.00	5.00	5.00
10	2.85	2.84	2.86	2.85	7.18	7.18	7.18	7.18	3.43	3.43	3.43	3.43
11	1.96	1.96	1.96	1.96	0.73	0.78	0.80	0.77	1.83	1.83	1.83	1.83
12	4.13	4.13	4.13	4.13	10.49	10.49	10.49	10.49	5.01	5.12	5.08	5.07
13	1.90	1.63	1.72	1.75	8.88	9.00	8.77	8.85	2.03	1.90	1.86	1.93
14	0.00	0.00	0.00	0.00	2.85	2.85	2.85	2.85	0.00	0.00	0.00	0.00
15	0.00	0.00	0.00	0.00	6.65	6.65	6.65	6.65	0.00	0.00	0.00	0.00
16	2.20	2.20	2.20	2.20	5.36	5.34	5.35	5.35	2.03	2.03	2.03	2.03
17	0.85	0.85	0.85	0.85	2.30	2.23	2.22	2.25	0.96	0.96	0.96	0.96
18	0.76	0.76	0.76	0.76	2.04	2.04	2.04	2.04	0.83	0.83	0.83	0.83
19	1.23	1.22	1.24	1.23	3.51	3.51	3.51	3.51	1.50	1.51	1.52	1.51
20	0.75	0.75	0.75	0.75	2.55	2.55	2.55	2.55	0.82	0.82	0.82	0.82

21	0.00	0.00	0.00	0.00	5.85	5.85	5.85	5.85	0.00	0.00	0.00	0.00
22	2.55	2.55	2.55	2.55	5.80	5.80	5.80	5.80	2.60	2.61	2.62	2.61
23	5.80	5.80	5.80	5.80	15.07	15.09	15.08	15.08	14.61	14.62	14.63	14.62
24	4.25	4.25	4.25	4.25	1.95	1.95	1.95	1.95	4.32	4.34	4.33	4.33
25	8.39	8.13	8.08	8.20	1.13	1.17	1.15	1.15	8.39	8.31	8.32	8.34
26	1.25	1.25	1.25	1.25	7.55	7.55	7.55	7.55	2.03	2.03	2.03	2.03
27	0.30	0.30	0.30	0.30	2.31	2.29	2.30	2.30	0.35	0.35	0.35	0.35
28	1.55	1.55	1.55	1.55	4.20	4.25	4.15	4.20	3.33	3.33	3.33	3.33
29	0.73	0.83	0.69	0.75	2.27	1.96	1.92	2.05	0.80	0.74	0.74	0.76
30	9.85	9.85	9.85	9.85	1.05	1.04	1.06	1.05	2.26	2.22	2.27	2.25
31	0.10	0.12	0.14	0.12	0.42	0.43	0.44	0.43	0.11	0.11	0.11	0.11
32	8.45	8.45	8.45	8.45	30.67	30.68	30.66	30.67	8.44	8.44	8.44	8.44
33	0.96	0.94	0.95	0.95	2.40	2.40	2.40	2.40	0.86	0.87	0.88	0.87
34	0.75	0.75	0.75	0.75	3.44	3.45	3.46	3.45	0.73	0.73	0.73	0.73
35	27.80	28.10	27.50	27.80	10.45	10.45	10.45	10.45	25.30	25.30	25.30	25.30
36	1.31	1.23	1.21	1.25	3.35	3.35	3.35	3.35	3.12	3.12	3.12	3.12
37	0.00	0.00	0.00	0.00	16.20	16.20	16.20	16.20	0.00	0.00	0.00	0.00
38	1.75	1.75	1.75	1.75	1.84	1.85	1.83	1.84	4.81	4.81	4.81	4.81
39	4.65	4.65	4.65	4.65	4.73	4.73	4.73	4.73	1.44	1.43	1.42	1.43
40	0.31	0.31	0.31	0.31	0.46	0.40	0.40	0.42	0.94	0.91	0.91	0.92
41	1.64	1.62	1.63	1.63	1.57	1.60	1.60	1.59	4.74	4.74	4.80	4.76
42	8.34	8.34	8.34	8.34	8.51	8.60	8.60	8.57	21.14	21.20	21.20	21.18
43	0.91	0.97	0.97	0.95	0.83	0.83	0.83	0.83	2.40	2.39	2.41	2.40

44	1.15	1.15	1.15	1.15	1.23	1.23	1.23	1.23	3.34	3.35	3.36	3.35
45	0.80	0.80	0.80	0.80	0.74	0.74	0.74	0.74	2.26	2.26	2.26	2.26
46	0.71	0.73	0.75	0.73	0.75	0.84	0.84	0.81	1.85	1.85	1.85	1.85
47	1.21	1.21	1.21	1.21	1.33	1.33	1.33	1.33	2.95	2.95	2.95	2.95
48	2.45	2.45	2.45	2.45	2.37	2.37	2.37	2.37	0.96	0.96	0.99	0.97

[a] Numbering corresponds to the 2–DE gel in Fig. 2.

Supplementary Table S4 Summary statistics of DGE tags in Japanese apricot after GA_4 treatment in control and A libraries.

**Summary**		**Control**	**A**
Raw Data	Total	3620084	3451950
	Distinct Tag	390999	375393
Clean Tag	Total number	3391578	3244431
	Distinct Tag number	163411	168839
All Tag Mapping to Gene	Total number	693822	732349
	Total % of clean tag	20.46%	22.57%
	Distinct Tag number	28165	30199
	Distinct Tag % of clean tag	17.24%	17.89%
Unambiguous Tag Mapping to Gene	Total number	688544	725107
	Total % of clean tag	20.30%	22.35%
	Distinct Tag number	27896	29900
	Distinct Tag % of clean tag	17.07%	17.71%
All Tag-mapped Genes	number	12757	13340
	% of ref genes	44.45%	46.48%
Unambiguous Tag-mapped Genes	number	12578	13137
	% of ref genes	43.82%	45.77%
Mapping to Genome	Total number	1812504	1603808
	Total % of clean tag	53.44%	49.43%
	Distinct Tag number	69110	73506
	Distinct Tag % of clean tag	42.29%	43.54%
Unknown Tag	Total number	885252	908274
	Total % of clean tag	26.10%	27.99%
	Distinct Tag number	66136	65134
	Distinct Tag % of clean tag	40.47%	38.58%

Supplementary Table S5 The up-regulated and down-regulated genes in Japanese apricot after GA_4 treatment (10 days).

Gene	log_2 Ratio(A/Control)	P-Value	FDR
ppa020752m|PACid:17666384	12.882452	7.11E-77	1.00E-74
ppa005526m|PACid:17648635	12.87670878	1.45E-76	1.98E-74
ppa010862m|PACid:17653615	12.55314928	2.46E-61	2.47E-59
ppa006857m|PACid:17658300	12.48507456	1.54E-58	1.47E-56
ppa007395m|PACid:17655705	12.19383333	5.67E-48	4.23E-46
ppa016543m|PACid:17650738	12.01192607	2.22E-42	1.47E-40
ppa005563m|PACid:17664224	11.82853321	2.09E-37	1.24E-35
ppa017189m|PACid:17653044	11.60408986	4.01E-32	2.01E-30
ppa020405m|PACid:17657243	11.01192607	1.47E-21	5.29E-20
ppa004774m|PACid:17641694	10.92332749	2.58E-20	8.64E-19
ppa010650m|PACid:17665966	10.75321675	3.87E-18	1.18E-16
ppa003587m|PACid:17656052	10.70043972	1.62E-17	4.72E-16
ppa025313m|PACid:17657410	10.70043972	1.62E-17	4.71E-16
ppa010476m|PACid:17651721	10.50084188	2.42E-15	6.30E-14
ppa019741m|PACid:17655782	10.50084188	2.42E-15	6.28E-14
ppa009870m|PACid:17645209	10.46964182	4.95E-15	1.26E-13
ppa021935m|PACid:17666610	10.46964182	4.95E-15	1.26E-13
ppa017962m|PACid:17665879	10.43775207	1.01E-14	2.51E-13
ppa010314m|PACid:17641658	10.43775207	1.01E-14	2.51E-13
ppa012971m|PACid:17658092	10.40514146	2.07E-14	5.08E-13
ppa010893m|PACid:17642568	10.15481811	3.10E-12	6.49E-11
ppa001782m|PACid:17646120	9.990103964	5.43E-11	1.04E-09
ppa005532m|PACid:17658974	9.700439718	3.98E-09	6.47E-08
ppa021285m|PACid:17656504	9.59058705	1.66E-08	2.52E-07
ppa007744m|PACid:17644219	9.531381461	3.40E-08	4.99E-07
ppa004903m|PACid:17666247	9.469641817	6.96E-08	9.80E-07
ppa000618m|PACid:17643020	9.469641817	6.96E-08	9.79E-07
ppa027202m|PACid:17655696	9.469641817	6.96E-08	9.78E-07
ppa023842m|PACid:17665296	9.405141463	1.42E-07	1.93E-06
ppa008418m|PACid:17642361	9.337621902	2.91E-07	3.80E-06
ppa002104m|PACid:17652890	9.337621902	2.91E-07	3.79E-06
ppa012381m|PACid:17651185	9.337621902	2.91E-07	3.79E-06
ppa018651m|PACid:17668648	9.266786541	5.96E-07	7.45E-06
ppa008559m|PACid:17650323	9.266786541	5.96E-07	7.44E-06
ppa010698m|PACid:17641243	9.194756854	1.22E-06	1.45E-05
ppa008126m|PACid:17648010	9.194756854	1.22E-06	1.45E-05
ppa024800m|PACid:17653974	9.194756854	1.22E-06	1.44E-05
ppa003158m|PACid:17660551	9.194756854	1.22E-06	1.44E-05
ppa014802m|PACid:17663559	9.194756854	1.22E-06	1.44E-05
ppb011302m|PACid:17662919	9.116343961	2.49E-06	2.80E-05

ppa023999m|PACid:17650179	9.116343961	2.49E-06	2.80E-05
ppa012371m|PACid:17656309	9.116343961	2.49E-06	2.79E-05
ppa020043m|PACid:17657409	9.116343961	2.49E-06	2.79E-05
ppa004709m|PACid:17649321	9.116343961	2.49E-06	2.79E-05
ppa022961m|PACid:17645522	9.116343961	2.49E-06	2.79E-05
ppa013681m|PACid:17661666	9.116343961	2.49E-06	2.78E-05
ppa012101m|PACid:17659111	9.116343961	2.49E-06	2.78E-05
ppa003848m|PACid:17655130	9.116343961	2.49E-06	2.78E-05
ppa019010m|PACid:17665726	9.033423002	5.10E-06	5.42E-05
ppa011478m|PACid:17653594	9.033423002	5.10E-06	5.41E-05
ppa006872m|PACid:17651641	9.033423002	5.10E-06	5.41E-05
ppa025978m|PACid:17641290	8.945443836	1.04E-05	0.000106488
ppa022427m|PACid:17644356	8.945443836	1.04E-05	0.000106398
ppa014756m|PACid:17669137	8.945443836	1.04E-05	0.000106308
ppa014614m|PACid:17656152	8.945443836	1.04E-05	0.000106219
ppa004215m|PACid:17648712	8.945443836	1.04E-05	0.000106129
ppa024779m|PACid:17663048	8.945443836	1.04E-05	0.00010604
ppa021128m|PACid:17656482	8.945443836	1.04E-05	0.000105951
ppa003347m|PACid:17652060	8.945443836	1.04E-05	0.000105862
ppa017444m|PACid:17645393	8.945443836	1.04E-05	0.000105773
ppa013715m|PACid:17640823	8.851749041	2.13E-05	0.000204333
ppa019145m|PACid:17652879	8.851749041	2.13E-05	0.000204171
ppa018846m|PACid:17656837	8.851749041	2.13E-05	0.000204009
ppa001744m|PACid:17668761	8.851749041	2.13E-05	0.000203848
ppa023786m|PACid:17668232	8.754887502	4.36E-05	0.00039536
ppa005184m|PACid:17661748	8.754887502	4.36E-05	0.000395064
ppa009277m|PACid:17657637	8.754887502	4.36E-05	0.000394768
ppa005073m|PACid:17668031	8.754887502	4.36E-05	0.000394472
ppa010654m|PACid:17664478	8.754887502	4.36E-05	0.000394177
ppa014799m|PACid:17658988	8.754887502	4.36E-05	0.000393883
ppa005777m|PACid:17644497	8.754887502	4.36E-05	0.000393588
ppa013286m|PACid:17642761	8.754887502	4.36E-05	0.000393295
ppa000853m|PACid:17659309	8.754887502	4.36E-05	0.000393001
ppa009535m|PACid:17663221	8.754887502	4.36E-05	0.000392709
ppa003422m|PACid:17667449	8.754887502	4.36E-05	0.000392416
ppa006913m|PACid:17667951	8.647458426	8.92E-05	0.000749083
ppa026190m|PACid:17655231	8.647458426	8.92E-05	0.000748563
ppa008970m|PACid:17653599	8.647458426	8.92E-05	0.000748044
ppa004021m|PACid:17644590	8.647458426	8.92E-05	0.000747525
ppa007343m|PACid:17644328	8.647458426	8.92E-05	0.000747007
ppa020339m|PACid:17667191	8.647458426	8.92E-05	0.000746489
ppa004413m|PACid:17661503	7.508264803	1.67E-208	9.17E-206
ppa022764m|PACid:17661764	6.958134874	8.11E-71	1.02E-68
ppb006257m|PACid:17668501	6.411583044	2.24E-117	6.16E-115

ppa008482m|PACid:17662536	6.192501795	1.76E-80	2.76E-78
ppa010081m|PACid:17661637	5.707008097	1.15E-28	5.30E-27
ppa011448m|PACid:17646081	5.292282633	3.87E-21	1.34E-19
ppa010953m|PACid:17662037	5.284005612	3.20E-90	5.95E-88
ppa015631m|PACid:17664295	5.272807682	1.52E-40	9.70E-39
ppa005598m|PACid:17642571	5.171735159	1.04E-55	9.42E-54
ppa007036m|PACid:17651282	5.085303656	3.78E-18	1.16E-16
ppa007458m|PACid:17644502	5.063532191	7.50E-18	2.26E-16
ppa004132m|PACid:17652721	5.063532191	7.50E-18	2.26E-16
ppa008310m|PACid:17657706	5.059188087	1.30E-42	8.66E-41
ppa010163m|PACid:17654179	5.036716898	4.83E-99	1.08E-96
ppa003578m|PACid:17653567	5.029123421	1.11E-33	5.88E-32
ppa011385m|PACid:17661231	4.926063985	1.87E-38	1.13E-36
ppa026306m|PACid:17655756	4.831602468	6.16E-29	2.87E-27
ppa020922m|PACid:17661603	4.763915661	2.72E-14	6.61E-13
ppa004722m|PACid:17660068	4.734093742	2.68E-20	8.95E-19
ppa014596m|PACid:17662779	4.734093742	2.68E-20	8.92E-19
ppa001718m|PACid:17657879	4.712133076	0	0
ppa003286m|PACid:17661399	4.677663845	1.24E-92	2.38E-90
ppa020423m|PACid:17663376	4.540530044	1.91E-28	8.76E-27
ppa003487m|PACid:17649448	4.508585531	3.78E-181	1.90E-178
ppa026396m|PACid:17654737	4.456093333	2.34E-11	4.62E-10
ppa021733m|PACid:17642045	4.440680444	3.37E-16	9.14E-15
ppb021546m|PACid:17650603	4.421137699	4.58E-11	8.82E-10
ppa003507m|PACid:17663874	4.310882311	3.41E-10	6.13E-09
ppa026775m|PACid:17660787	4.254965882	1.03E-99	2.34E-97
ppa005158m|PACid:17665900	4.24309328	3.37E-69	4.11E-67
ppa007271m|PACid:17642246	4.2410081	7.01E-14	1.66E-12
ppa017430m|PACid:17643481	4.189421177	1.72E-21	6.13E-20
ppa005715m|PACid:17641909	4.178503091	2.00E-25	8.21E-24
ppa020764m|PACid:17653414	4.140071524	2.02E-32	1.02E-30
ppa006610m|PACid:17661856	4.124296707	6.42E-97	1.31E-94
ppa007765m|PACid:17660217	4.086307761	8.99E-20	2.94E-18
ppa006606m|PACid:17665894	3.960492862	1.01E-67	1.17E-65
ppa020321m|PACid:17655609	3.937535913	7.26E-21	2.47E-19
ppa019974m|PACid:17646594	3.936856017	4.52E-37	2.67E-35
ppa012595m|PACid:17667617	3.929576646	2.26E-30	1.10E-28
ppa009806m|PACid:17647648	3.929552404	4.40E-69	5.32E-67
ppa015286m|PACid:17666944	3.921487972	1.31E-07	1.78E-06
ppa005640m|PACid:17652286	3.817796669	4.83E-07	6.11E-06
ppb013860m|PACid:17665083	3.790431619	1.58E-15	4.16E-14
ppa005141m|PACid:17666232	3.763015383	9.23E-07	1.12E-05
ppa017188m|PACid:17661861	3.763015383	9.23E-07	1.12E-05
ppa004380m|PACid:17641292	3.731115165	1.80E-36	1.02E-34

ppa007566m|PACid:17644782	3.729049927	1.33E-25	5.57E-24
ppa018615m|PACid:17644562	3.72591981	1.71E-17	4.96E-16
ppa009630m|PACid:17646182	3.707944001	1.76E-06	2.04E-05
ppa004219m|PACid:17642166	3.707008097	1.57E-22	5.80E-21
ppb011173m|PACid:17660206	3.69538649	4.76E-09	7.62E-08
ppa002490m|PACid:17667233	3.690353839	9.09E-25	3.64E-23
ppa001896m|PACid:17667538	3.663015836	0	0
ppa012395m|PACid:17649289	3.634807633	6.14E-46	4.42E-44
ppa000296m|PACid:17654244	3.616047531	1.71E-08	2.59E-07
ppa006442m|PACid:17664765	3.586998768	6.38E-06	6.69E-05
ppa003797m|PACid:17667031	3.586998768	6.38E-06	6.68E-05
ppa002608m|PACid:17643239	3.576446982	1.55E-29	7.35E-28
ppa005142m|PACid:17662404	3.573991383	3.24E-08	4.78E-07
ppa027154m|PACid:17667144	3.568715269	1.59E-66	1.79E-64
ppa003134m|PACid:17659748	3.547295445	2.26E-176	1.09E-173
ppa001113m|PACid:17664470	3.527003211	3.69E-95	7.43E-93
ppa006923m|PACid:17650909	3.524723079	6.25E-26	2.64E-24
ppa002136m|PACid:17668058	3.522498414	1.21E-05	0.000121455
ppa001356m|PACid:17664449	3.522498414	1.21E-05	0.000121354
ppa012742m|PACid:17648498	3.511419695	0	0
ppa001287m|PACid:17668109	3.507816151	5.24E-111	1.38E-108
ppa018445m|PACid:17649288	3.506625008	7.52E-52	6.27E-50
ppa012380m|PACid:17645332	3.489133595	6.14E-10	1.07E-08
ppa012123m|PACid:17664106	3.474519923	6.31E-12	1.29E-10
ppa006304m|PACid:17654545	3.454978853	2.28E-05	0.000216033
ppa006153m|PACid:17656361	3.454978853	2.28E-05	0.000215864
ppa010683m|PACid:17648689	3.454978853	2.28E-05	0.000215695
ppa013577m|PACid:17641362	3.454978853	2.28E-05	0.000215527
ppa001085m|PACid:17647032	3.4381575	7.48E-18	2.26E-16
ppa014211m|PACid:17664550	3.413814358	1.22E-41	7.86E-40
ppa014104m|PACid:17669275	3.385314035	4.87E-17	1.39E-15
ppa023646m|PACid:17651232	3.385314035	4.05E-09	6.56E-08
ppa009128m|PACid:17642179	3.385080002	5.66E-21	1.94E-19
ppa009405m|PACid:17649673	3.361023152	2.13E-22	7.83E-21
ppa027059m|PACid:17661470	3.348578131	1.69E-16	4.68E-15
ppa016304m|PACid:17640504	3.344699403	7.60E-07	9.37E-06
ppa014605m|PACid:17659609	3.325425539	2.95E-14	7.16E-13
ppa008758m|PACid:17651869	3.312113805	8.05E-05	0.000685552
ppa025728m|PACid:17642577	3.311292926	3.14E-34	1.68E-32
ppa023006m|PACid:17645227	3.293785131	1.42E-06	1.66E-05
ppa010099m|PACid:17652474	3.293785131	1.42E-06	1.66E-05
ppa016354m|PACid:17667664	3.269969472	4.97E-10	8.76E-09
ppa006836m|PACid:17649297	3.259674714	1.88E-13	4.35E-12
ppa001841m|PACid:17647655	3.249801144	2.98E-20	9.88E-19

ppa017252m|PACid:17656265	3.2410081	2.64E-06	2.93E-05
ppa018950m|PACid:17664119	3.238079727	9.22E-10	1.59E-08
ppa017146m|PACid:17643976	3.223265459	3.49E-28	1.58E-26
ppa010328m|PACid:17668507	3.220453938	2.79E-139	9.62E-137
ppa013722m|PACid:17647538	3.218108535	2.52E-16	6.93E-15
ppa009987m|PACid:17655949	3.189479225	8.01E-108	2.02E-105
ppa002544m|PACid:17663600	3.186226814	4.89E-06	5.22E-05
ppa022356m|PACid:17665362	3.186226814	4.89E-06	5.22E-05
ppa009698m|PACid:17658186	3.186226814	4.89E-06	5.21E-05
ppa003377m|PACid:17667530	3.177113062	3.50E-65	3.75E-63
ppa003805m|PACid:17649186	3.171961269	4.31E-14	1.03E-12
ppa006994m|PACid:17646766	3.150779952	5.78E-17	1.64E-15
ppa016888m|PACid:17653515	3.098490275	0	0
ppa026947m|PACid:17668942	3.065845403	3.24E-14	7.86E-13
ppa006746m|PACid:17666547	3.062800787	5.62E-07	7.07E-06
ppa002298m|PACid:17669014	3.062800787	6.89E-10	1.20E-08
ppa009745m|PACid:17649428	3.040641984	3.09E-54	2.63E-52
ppa005472m|PACid:17646666	3.031548836	3.57E-08	5.20E-07
ppa002187m|PACid:17650626	3.014395643	4.28E-42	2.81E-40
ppa008048m|PACid:17665082	3.010210199	3.05E-05	0.000283244
ppa004291m|PACid:17641189	3.002036267	2.29E-09	3.83E-08
ppa002353m|PACid:17665436	3.001697089	1.56E-25	6.49E-24
ppa010986m|PACid:17643075	2.985814094	1.04E-69	1.28E-67
ppa021895m|PACid:17641301	2.970666506	1.88E-06	2.15E-05
ppa017028m|PACid:17646612	2.966907362	4.15E-31	2.05E-29
ppa012612m|PACid:17658654	2.957732486	1.09E-48	8.37E-47
ppa018749m|PACid:17663428	2.953933357	1.05E-16	2.93E-15
ppa014216m|PACid:17660129	2.949921579	1.29E-78	1.97E-76
ppa009844m|PACid:17651763	2.945709844	5.58E-05	0.000491113
ppa009022m|PACid:17664534	2.945709844	5.58E-05	0.000490755
ppa004941m|PACid:17664661	2.935459748	3.71E-25	1.50E-23
ppa004306m|PACid:17659262	2.908743293	8.74E-55	7.66E-53
ppa009003m|PACid:17661918	2.904171094	1.37E-08	2.09E-07
ppa005031m|PACid:17643115	2.878190283	0.000101478	0.000838392
ppa010195m|PACid:17665741	2.859626629	5.38E-145	2.03E-142
ppa024083m|PACid:17642369	2.853194999	3.60E-15	9.25E-14
ppa012487m|PACid:17646529	2.852606855	1.78E-90	3.37E-88
ppa008322m|PACid:17647011	2.850033174	8.14E-13	1.78E-11
ppa011593m|PACid:17652071	2.840338325	4.31E-16	1.16E-14
ppa012765m|PACid:17659944	2.837423244	4.04E-118	1.19E-115
ppa021994m|PACid:17665751	2.833750657	6.99E-07	8.65E-06
ppa005402m|PACid:17657424	2.823940881	7.72E-16	2.06E-14
ppa020717m|PACid:17649019	2.81724259	1.32E-35	7.21E-34
ppa009905m|PACid:17663475	2.807658871	1.57E-26	6.76E-25

ppa021967m|PACid:17661398	2.800351534	8.00E-08	1.12E-06
ppa000692m|PACid:17647767	2.791549219	6.03E-10	1.05E-08
ppa006386m|PACid:17649503	2.790431619	1.26E-06	1.48E-05
ppa009739m|PACid:17668202	2.788966058	2.81E-29	1.33E-27
ppa010731m|PACid:17661078	2.788089063	5.35E-42	3.50E-40
ppa018224m|PACid:17662746	2.763015383	2.01E-05	0.000193583
ppa006665m|PACid:17660601	2.763015383	2.01E-05	0.000193429
ppa022841m|PACid:17665865	2.763015383	2.01E-05	0.000193275
ppa007958m|PACid:17658602	2.753652001	4.11E-24	1.60E-22
ppa009106m|PACid:17665424	2.745771492	2.25E-06	2.55E-05
ppa007697m|PACid:17654070	2.7112883	1.46E-93	2.90E-91
ppa004336m|PACid:17662282	2.707944001	3.60E-05	0.000329879
ppa009934m|PACid:17663557	2.707195326	4.93E-21	1.70E-19
ppa010287m|PACid:17662659	2.706192368	3.03E-217	1.83E-214
ppa027017m|PACid:17649955	2.699684578	4.02E-06	4.37E-05
ppa001756m|PACid:17657485	2.691292325	4.06E-205	2.14E-202
ppa018400m|PACid:17657800	2.670967067	2.97E-122	9.44E-120
ppa012846m|PACid:17643345	2.662350409	3.55E-83	5.87E-81
ppa003684m|PACid:17649014	2.658130779	1.01E-23	3.87E-22
ppa010586m|PACid:17658972	2.634463511	1.85E-47	1.36E-45
ppa011310m|PACid:17652259	2.61819883	1.89E-08	2.85E-07
ppa002585m|PACid:17661611	2.613293283	6.77E-50	5.38E-48
ppa002340m|PACid:17655370	2.612835678	1.13E-36	6.53E-35
ppa021645m|PACid:17656715	2.610750565	2.45E-24	9.60E-23
ppa019732m|PACid:17644727	2.607873599	1.43E-06	1.67E-05
ppa002534m|PACid:17664225	2.606325238	5.33E-11	1.02E-09
ppa006149m|PACid:17661849	2.596710161	3.87E-09	6.32E-08
ppa016286m|PACid:17651122	2.586998768	1.10E-11	2.23E-10
ppa012362m|PACid:17643910	2.586998768	0.000113872	0.000933136
ppa004298m|PACid:17665290	2.577577574	1.04E-27	4.62E-26
ppa005749m|PACid:17650906	2.569357067	1.82E-27	7.99E-26
ppa004078m|PACid:17641348	2.558092666	6.56E-33	3.33E-31
ppa001327m|PACid:17656420	2.554508704	4.00E-12	8.28E-11
ppa011156m|PACid:17662422	2.553544404	2.23E-05	0.000211542
ppa010034m|PACid:17653895	2.538414681	0	0
ppa019653m|PACid:17644724	2.537083096	5.14E-16	1.38E-14
ppa009284m|PACid:17669366	2.53449337	6.97E-12	1.42E-10
ppa006905m|PACid:17651659	2.522498414	4.41E-06	4.79E-05
ppa019257m|PACid:17655452	2.522498414	2.25E-17	6.51E-16
ppa008715m|PACid:17655161	2.507024833	8.83E-07	1.07E-05
ppa005137m|PACid:17663194	2.498045283	9.01E-78	1.33E-75
ppa006352m|PACid:17653826	2.494278568	2.11E-11	4.17E-10
ppa006679m|PACid:17666700	2.490515704	3.22E-38	1.92E-36
ppa017516m|PACid:17653116	2.487331934	1.16E-26	5.01E-25

ppa008147m|PACid:17649944	2.476123555	3.93E-48	2.97E-46
ppa011725m|PACid:17663445	2.474937734	4.22E-234	3.00E-231
ppa012433m|PACid:17644508	2.455721935	3.08E-10	5.57E-09
ppa007157m|PACid:17645576	2.448847839	3.55E-21	1.23E-19
ppa008636m|PACid:17640963	2.445986088	6.83E-05	0.000591719
ppa002847m|PACid:17662760	2.445986088	6.83E-05	0.000591295
ppa007671m|PACid:17663378	2.445986088	6.83E-05	0.000590872
ppa005683m|PACid:17644017	2.445986088	6.83E-05	0.000590449
ppa009872m|PACid:17652907	2.445986088	6.83E-05	0.000590027
ppa006903m|PACid:17658689	2.445783448	2.89E-15	7.48E-14
ppa001336m|PACid:17652394	2.441987566	1.27E-08	1.95E-07
ppa000260m|PACid:17660438	2.439048473	2.60E-09	4.28E-08
ppa011793m|PACid:17666742	2.435887927	2.72E-50	2.20E-48
ppa004285m|PACid:17668433	2.432355517	5.63E-23	2.11E-21
ppa001824m|PACid:17642564	2.429843434	2.67E-06	2.96E-05
ppa003854m|PACid:17641906	2.414792788	1.66E-36	9.42E-35
ppa002598m|PACid:17667389	2.413366746	4.48E-09	7.22E-08
ppa008273m|PACid:17668407	2.405728697	6.47E-21	2.21E-19
ppa008826m|PACid:17665521	2.399591209	7.03E-60	6.90E-58
ppa001828m|PACid:17665676	2.390914705	0.00011867	0.000967206
ppa002738m|PACid:17648723	2.386746124	3.23E-10	5.83E-09
ppa023881m|PACid:17644592	2.385704005	2.33E-05	0.000219751
ppa008472m|PACid:17653732	2.375301446	1.55E-19	4.99E-18
ppb010066m|PACid:17651729	2.369144481	1.14E-36	6.54E-35
ppa012242m|PACid:17669132	2.36205896	1.00E-88	1.81E-86
ppa005008m|PACid:17667126	2.346922474	7.95E-06	8.22E-05
ppa025709m|PACid:17665691	2.344589903	5.44E-15	1.38E-13
ppa013451m|PACid:17668078	2.336525471	6.06E-13	1.34E-11
ppa003682m|PACid:17641618	2.309104055	1.16E-15	3.08E-14
ppa003047m|PACid:17658308	2.304925186	9.21E-22	3.35E-20
ppa006294m|PACid:17656965	2.303603437	1.36E-05	0.000135676
ppa022569m|PACid:17643626	2.285611199	6.89E-05	0.000594696
ppa007273m|PACid:17659637	2.271328927	4.67E-09	7.49E-08
ppa003830m|PACid:17655317	2.262554388	2.43E-11	4.79E-10
ppa006759m|PACid:17659157	2.259464008	2.28E-08	3.43E-07
ppa007054m|PACid:17666367	2.257024531	5.22E-61	5.21E-59
ppa006313m|PACid:17650503	2.243037938	2.98E-12	6.25E-11
ppa027060m|PACid:17651214	2.232834168	0.000117598	0.000960407
ppa005437m|PACid:17656870	2.224023507	9.56E-10	1.64E-08
ppa027069m|PACid:17656884	2.220482831	1.88E-07	2.50E-06
ppa006263m|PACid:17666726	2.218214785	1.36E-18	4.25E-17
ppa006989m|PACid:17645364	2.213567092	3.88E-49	3.00E-47
ppa000657m|PACid:17648606	2.212231919	1.64E-54	1.40E-52
ppa010319m|PACid:17665276	2.210114092	4.63E-09	7.44E-08

ppa004909m|PACid:17645285	2.206586718	6.49E-08	9.21E-07
ppa012860m|PACid:17662261	2.205653671	0	0
ppa000725m|PACid:17654584	2.1928361	1.34E-05	0.000133466
ppa011263m|PACid:17655916	2.175642107	9.18E-31	4.49E-29
ppa022307m|PACid:17665608	2.17457511	4.06E-16	1.10E-14
ppa003236m|PACid:17645819	2.1672055	1.55E-06	1.81E-05
ppa006370m|PACid:17660238	2.166809028	6.69E-05	0.000580929
ppa003011m|PACid:17647193	2.166809028	6.69E-05	0.000580511
ppa010155m|PACid:17658379	2.158272681	4.51E-09	7.26E-08
ppa005272m|PACid:17644771	2.150779952	2.25E-05	0.000213892
ppa007427m|PACid:17651235	2.150779952	2.25E-05	0.000213725
ppa011034m|PACid:17649621	2.147743308	4.82E-12	9.90E-11
ppa008187m|PACid:17648690	2.143676811	7.65E-06	7.93E-05
ppa010022m|PACid:17660883	2.140835865	1.74E-33	9.08E-32
ppa003992m|PACid:17668992	2.135424926	7.53E-09	1.17E-07
ppa007791m|PACid:17662094	2.130453775	9.31E-20	3.04E-18
ppa006894m|PACid:17659692	2.120376425	3.08E-62	3.15E-60
ppa008744m|PACid:17648845	2.119239136	1.05E-07	1.45E-06
ppa010066m|PACid:17641455	2.118413225	4.73E-17	1.35E-15
ppa009016m|PACid:17657337	2.117630494	0.000112414	0.000921813
ppa022249m|PACid:17664055	2.112209504	1.25E-08	1.93E-07
ppb022901m|PACid:17669180	2.10363993	3.84E-48	2.92E-46
ppa000285m|PACid:17641496	2.102882681	5.22E-10	9.17E-09
ppa001571m|PACid:17649926	2.090667972	1.15E-13	2.67E-12
ppa002201m|PACid:17651679	2.088781375	1.31E-68	1.55E-66
ppa005285m|PACid:17644080	2.078961326	3.38E-68	3.93E-66
ppa1027190m|PACid:17647925	2.071845125	1.96E-23	7.44E-22
ppa008338m|PACid:17660922	2.06529146	2.46E-06	2.77E-05
ppa014506m|PACid:17643053	2.062800787	8.43E-07	1.03E-05
ppa004373m|PACid:17654058	2.055512428	2.22E-19	7.10E-18
ppa009871m|PACid:17649917	2.0442739	8.35E-18	2.50E-16
ppa009849m|PACid:17640310	2.038942242	5.53E-40	3.39E-38
ppa002471m|PACid:17645482	2.03718492	4.78E-07	6.06E-06
ppa010379m|PACid:17640308	2.033236329	1.39E-06	1.64E-05
ppa011230m|PACid:17651381	2.031926641	4.08E-06	4.43E-05
ppa019687m|PACid:17641026	2.028358555	3.43E-19	1.09E-17
ppa013299m|PACid:17662997	2.0256306	3.53E-05	0.000324546
ppa006286m|PACid:17658744	2.0256306	3.53E-05	0.000324299
ppa008324m|PACid:17643702	2.021437171	1.11E-08	1.72E-07
ppa019639m|PACid:17655055	2.016713874	0.000104629	0.000862068
ppa007723m|PACid:17654186	2.010615327	1.27E-27	5.58E-26
ppa009067m|PACid:17657150	2.005373172	6.44E-12	1.32E-10
ppa013064m|PACid:17668965	1.99640388	3.29E-98	6.97E-96
ppa001765m|PACid:17655910	1.992096606	1.49E-62	1.55E-60

ppa001527m|PACid:17658170	1.987288948	2.57E-13	5.83E-12
ppa011044m|PACid:17648393	1.985973808	2.03E-30	9.91E-29
ppa006512m|PACid:17659307	1.975782604	1.27E-23	4.88E-22
ppa005294m|PACid:17667209	1.975324967	3.22E-157	1.34E-154
ppa013708m|PACid:17663915	1.970146522	3.76E-06	4.10E-05
ppa011352m|PACid:17643136	1.967179492	1.40E-10	2.62E-09
ppa007860m|PACid:17652672	1.965761247	5.88E-15	1.48E-13
ppa005902m|PACid:17660817	1.96531419	2.50E-07	3.29E-06
ppa014503m|PACid:17656023	1.964515019	1.64E-156	6.61E-154
ppa013368m|PACid:17668706	1.957425581	1.15E-09	1.97E-08
ppa006672m|PACid:17645548	1.940255414	9.54E-05	0.000793761
ppa011722m|PACid:17665133	1.932814488	4.45E-20	1.47E-18
ppa007490m|PACid:17658335	1.928387531	1.18E-06	1.40E-05
ppa010085m|PACid:17668703	1.928107082	1.80E-05	0.0001751
ppa018617m|PACid:17666468	1.923193705	1.09E-43	7.34E-42
ppa013103m|PACid:17667358	1.921487972	2.28E-07	3.01E-06
ppa008016m|PACid:17650754	1.917737038	1.23E-25	5.18E-24
ppa009136m|PACid:17658618	1.912345949	3.22E-16	8.76E-15
ppa025857m|PACid:17649431	1.911772817	5.28E-05	0.000466809
ppa003431m|PACid:17653591	1.911772817	5.28E-05	0.000466468
ppa004326m|PACid:17665938	1.904139985	2.49E-08	3.74E-07
ppa009496m|PACid:17650143	1.903940649	9.54E-88	1.67E-85
ppa023908m|PACid:17654425	1.899653175	1.91E-06	2.19E-05
ppa012289m|PACid:17665635	1.898068486	9.60E-10	1.65E-08
ppa004942m|PACid:17655293	1.897013567	2.43E-21	8.55E-20
ppa006010m|PACid:17643881	1.896255427	3.69E-07	4.74E-06
ppa003015m|PACid:17645696	1.89360719	7.16E-08	1.01E-06
ppa010093m|PACid:17649202	1.893545094	3.06E-51	2.51E-49
ppa009854m|PACid:17653845	1.883607288	7.64E-19	2.42E-17
ppa005120m|PACid:17652935	1.870908005	8.56E-05	0.000721408
ppa007962m|PACid:17645524	1.866004856	2.07E-28	9.46E-27
ppa005990m|PACid:17657699	1.862815621	2.48E-09	4.10E-08
ppa010782m|PACid:17665289	1.861394762	3.40E-15	8.76E-14
ppa000787m|PACid:17650148	1.859758808	2.54E-20	8.52E-19
ppa023045m|PACid:17659371	1.858656151	1.72E-06	1.98E-05
ppa011093m|PACid:17651028	1.853675358	4.72E-05	0.000422492
ppa005609m|PACid:17640450	1.853345826	2.40E-13	5.48E-12
ppa007071m|PACid:17658137	1.847824346	1.07E-22	3.96E-21
ppa014606m|PACid:17664257	1.841898353	2.02E-08	3.04E-07
ppa000340m|PACid:17643231	1.835464228	5.32E-07	6.71E-06
ppa005392m|PACid:17656199	1.823948896	2.96E-07	3.85E-06
ppa004917m|PACid:17665205	1.82101276	2.16E-36	1.22E-34
ppa005030m|PACid:17648649	1.817796669	1.53E-06	1.78E-05
ppa005016m|PACid:17647958	1.810843908	7.95E-06	8.23E-05

ppa011212m|PACid:17664062	1.810231683	8.49E-07	1.03E-05
ppa009417m|PACid:17667850	1.809306662	3.91E-23	1.48E-21
ppa005324m|PACid:17651642	1.807866244	1.75E-40	1.10E-38
ppa006660m|PACid:17653187	1.80168355	5.13E-08	7.36E-07
ppa011847m|PACid:17652536	1.801370747	1.40E-16	3.88E-15
ppa011780m|PACid:17663806	1.801127452	6.21E-10	1.08E-08
ppa006158m|PACid:17662577	1.798319083	8.96E-18	2.68E-16
ppa019519m|PACid:17661868	1.797708272	5.46E-14	1.29E-12
ppa003259m|PACid:17668991	1.791837015	2.31E-25	9.45E-24
ppa004860m|PACid:17658520	1.791383173	4.27E-57	3.94E-55
ppa010884m|PACid:17644160	1.776578222	4.10E-97	8.54E-95
ppa006253m|PACid:17664541	1.776493958	0.000121466	0.000988658
ppa010480m|PACid:17648423	1.776361777	7.50E-07	9.26E-06
ppa000586m|PACid:17657979	1.763615631	6.66E-05	0.00057907
ppa019611m|PACid:17665964	1.761756631	7.63E-15	1.91E-13
ppa006438m|PACid:17656982	1.759627402	1.29E-07	1.75E-06
ppa013690m|PACid:17655287	1.753776137	1.88E-162	8.13E-160
ppa007467m|PACid:17651604	1.750860036	1.89E-13	4.36E-12
ppa008777m|PACid:17656518	1.74900642	2.06E-51	1.71E-49
ppa008532m|PACid:17659420	1.74723393	1.68E-12	3.57E-11
ppa010245m|PACid:17652873	1.741360336	4.28E-10	7.57E-09
ppa003100m|PACid:17646787	1.727679041	7.72E-20	2.53E-18
ppa011717m|PACid:17658055	1.72378	8.19E-14	1.92E-12
ppa005160m|PACid:17654206	1.723101418	1.15E-38	6.94E-37
ppa006982m|PACid:17664116	1.722211154	2.14E-19	6.86E-18
ppa012991m|PACid:17659024	1.721580052	0	0
ppa006424m|PACid:17652757	1.718726476	6.04E-09	9.53E-08
ppa001275m|PACid:17659742	1.715143492	3.18E-05	0.000294462
ppa011708m|PACid:17651175	1.715143492	3.18E-05	0.000294237
ppa007548m|PACid:17655717	1.715143492	3.18E-05	0.000294012
ppa002878m|PACid:17659526	1.714241058	4.99E-59	4.83E-57
ppa002385m|PACid:17641472	1.71293932	1.75E-05	0.00017091
ppa003782m|PACid:17654827	1.707320132	5.47E-08	7.83E-07
ppa003910m|PACid:17640619	1.706211056	6.95E-24	2.68E-22
ppa012166m|PACid:17662798	1.70349809	5.18E-74	6.80E-72
ppa009500m|PACid:17661103	1.702897338	1.70E-08	2.57E-07
ppa005789m|PACid:17642531	1.699646811	1.63E-06	1.88E-05
ppa003457m|PACid:17664615	1.686756502	9.13E-05	0.000762467
ppa007720m|PACid:17666080	1.686756502	9.13E-05	0.00076194
ppa010015m|PACid:17648027	1.683117193	2.19E-45	1.56E-43
ppa005470m|PACid:17656374	1.681778673	5.00E-05	0.000446108
ppa005286m|PACid:17654365	1.680213601	3.15E-65	3.40E-63
ppa012548m|PACid:17658002	1.677689785	1.51E-05	0.000149185
ppa006895m|PACid:17662466	1.672379642	7.80E-07	9.59E-06

ppa003610m|PACid:17654292	1.671945802	1.41E-06	1.65E-05
ppa000468m|PACid:17650750	1.669851398	4.32E-07	5.51E-06
ppa012449m|PACid:17668812	1.668753325	1.33E-63	1.40E-61
ppa001976m|PACid:17650566	1.668053149	4.13E-14	9.95E-13
ppa011723m|PACid:17643268	1.658211483	2.01E-14	4.94E-13
ppa008791m|PACid:17643209	1.655965872	1.84E-121	5.72E-119
ppa012574m|PACid:17652999	1.652207063	5.36E-74	6.97E-72
ppa004229m|PACid:17654065	1.649312912	1.10E-08	1.71E-07
ppa003664m|PACid:17654382	1.648998981	1.71E-18	5.31E-17
ppa017571m|PACid:17668125	1.648738411	7.83E-05	0.000669616
ppa002923m|PACid:17649241	1.647958365	2.19E-06	2.49E-05
ppa000820m|PACid:17663080	1.636616141	6.68E-30	3.22E-28
ppa007035m|PACid:17656271	1.63085287	1.47E-284	1.11E-281
ppa001412m|PACid:17642131	1.630194265	1.31E-88	2.34E-86
ppa018286m|PACid:17645875	1.621624217	1.11E-05	0.000112891
ppa009834m|PACid:17641050	1.621119118	3.55E-14	8.59E-13
ppa007235m|PACid:17657810	1.618682291	5.36E-17	1.53E-15
ppa008438m|PACid:17642352	1.613782777	0.000121983	0.000992198
ppa008886m|PACid:17667313	1.613782777	0.000121983	0.000991531
ppa012923m|PACid:17649658	1.610983284	1.34E-49	1.05E-47
ppa003788m|PACid:17641669	1.609740481	4.63E-16	1.24E-14
ppa008642m|PACid:17646866	1.606399671	2.18E-09	3.63E-08
ppa016730m|PACid:17659022	1.604100573	1.61E-06	1.86E-05
ppa007227m|PACid:17662542	1.601701717	1.25E-08	1.92E-07
ppa017186m|PACid:17658778	1.589403844	5.98E-09	9.44E-08
ppa023043m|PACid:17651473	1.586998768	5.68E-05	0.000498572
ppa017605m|PACid:17652765	1.586998768	5.68E-05	0.000498211
ppa010530m|PACid:17665723	1.585264541	1.09E-13	2.55E-12
ppa000080m|PACid:17667623	1.57873391	4.47E-06	4.82E-05
ppa001833m|PACid:17641170	1.578291067	1.97E-12	4.17E-11
ppa000099m|PACid:17650267	1.577959113	1.11E-07	1.53E-06
ppa005700m|PACid:17655040	1.577432199	1.85E-18	5.71E-17
ppa008525m|PACid:17651173	1.574321615	7.94E-169	3.56E-166
ppa010274m|PACid:17666750	1.562227188	7.78E-09	1.21E-07
ppa002202m|PACid:17667115	1.557887259	1.32E-56	1.20E-54
ppa012613m|PACid:17661392	1.555109023	8.75E-05	0.000736128
ppa013340m|PACid:17642934	1.551499742	9.16E-55	7.97E-53
ppa007880m|PACid:17651364	1.550956329	1.34E-15	3.52E-14
ppa004821m|PACid:17650013	1.548645605	1.24E-05	0.00012419
ppa004490m|PACid:17661677	1.547630875	5.84E-13	1.30E-11
ppa010221m|PACid:17643128	1.544320516	1.78E-06	2.05E-05
ppa010944m|PACid:17641470	1.538442206	5.62E-09	8.94E-08
ppa025683m|PACid:17652395	1.537985492	1.38E-116	3.72E-114
ppa011893m|PACid:17645079	1.533553833	1.54E-78	2.33E-76

ppa006034m|PACid:17661265	1.532611185	8.37E-07	1.02E-05
ppa010078m|PACid:17641248	1.531266904	1.22E-07	1.67E-06
ppa000771m|PACid:17645318	1.530291855	3.38E-17	9.74E-16
ppa1027211m|PACid:17661916	1.523857524	8.51E-09	1.32E-07
ppa004833m|PACid:17658938	1.519554409	5.46E-22	1.99E-20
ppa016609m|PACid:17646065	1.517275693	3.42E-05	0.00031525
ppa011193m|PACid:17646869	1.511058153	3.61E-90	6.61E-88
ppa004637m|PACid:17646982	1.50864528	3.11E-17	8.98E-16
ppa004568m|PACid:17663074	1.504149986	9.68E-30	4.62E-28
ppa001810m|PACid:17648315	1.50060386	1.26E-17	3.69E-16
ppa003899m|PACid:17659080	1.500140058	4.34E-48	3.26E-46
ppa000255m|PACid:17654036	1.496446461	2.80E-07	3.66E-06
ppa000656m|PACid:17660986	1.490170118	7.43E-14	1.75E-12
ppa002350m|PACid:17656726	1.487987687	4.91E-13	1.10E-11
ppa004703m|PACid:17666113	1.487445995	2.49E-38	1.49E-36
ppa006299m|PACid:17665488	1.483632953	6.97E-31	3.43E-29
ppa006328m|PACid:17662823	1.482769155	2.51E-21	8.80E-20
ppa007653m|PACid:17662855	1.47942115	2.04E-09	3.41E-08
ppa003796m|PACid:17665462	1.47942115	2.41E-05	0.000227
ppa012591m|PACid:17641346	1.478796551	2.08E-143	7.60E-141
ppa007177m|PACid:17667690	1.478423439	4.22E-07	5.39E-06
ppa004544m|PACid:17643196	1.474594563	3.40E-78	5.07E-76
ppa002453m|PACid:17647608	1.472151182	8.66E-12	1.76E-10
ppa002985m|PACid:17654476	1.469283888	1.54E-11	3.08E-10
ppa002142m|PACid:17668526	1.46518036	1.12E-44	7.65E-43
ppa006651m|PACid:17655734	1.465122731	1.15E-08	1.78E-07
ppa004571m|PACid:17653993	1.462930005	2.94E-16	8.04E-15
ppa010404m|PACid:17660705	1.462797527	1.66E-07	2.23E-06
ppa017924m|PACid:17661839	1.455686872	4.72E-14	1.13E-12
ppa013301m|PACid:17663848	1.445180133	4.81E-10	8.48E-09
ppa013356m|PACid:17664380	1.442350404	2.87E-11	5.63E-10
ppa021958m|PACid:17653020	1.438520224	5.09E-11	9.74E-10
ppa001535m|PACid:17645027	1.429486412	7.81E-30	3.75E-28
ppa009692m|PACid:17654407	1.428118144	7.55E-11	1.43E-09
ppa009353m|PACid:17644381	1.414425799	4.64E-15	1.18E-13
ppa1027122m|PACid:17662689	1.411616537	6.16E-26	2.61E-24
ppa010827m|PACid:17667612	1.411142081	4.52E-05	0.000405741
ppa007118m|PACid:17651724	1.409612748	1.22E-17	3.58E-16
ppa002674m|PACid:17657248	1.407681125	2.23E-12	4.69E-11
ppa008787m|PACid:17651814	1.400396669	8.13E-05	0.000692006
ppa007127m|PACid:17665180	1.397806676	1.10E-09	1.87E-08
ppa007519m|PACid:17646051	1.394504554	8.17E-07	1.00E-05
ppa001973m|PACid:17652447	1.393914208	3.82E-07	4.91E-06
ppa009903m|PACid:17655203	1.392023505	1.65E-55	1.48E-53

ppa012973m|PACid:17659839	1.38590815	8.60E-77	1.18E-74
ppa009850m|PACid:17662201	1.385838806	1.80E-11	3.57E-10
ppa006177m|PACid:17667329	1.379264094	1.29E-08	1.97E-07
ppa000775m|PACid:17642849	1.377199382	5.92E-12	1.22E-10
ppa005584m|PACid:17662113	1.370666012	2.08E-13	4.78E-12
ppa002583m|PACid:17662966	1.368936645	4.90E-28	2.19E-26
ppa008320m|PACid:17653469	1.367063008	2.15E-06	2.44E-05
ppa014283m|PACid:17669250	1.360864739	1.44E-10	2.69E-09
ppa002034m|PACid:17647482	1.359684111	1.78E-06	2.05E-05
ppa007900m|PACid:17655851	1.350826141	6.13E-28	2.73E-26
ppa001897m|PACid:17650737	1.348075286	8.24E-05	0.00069908
ppa011637m|PACid:17656391	1.344834517	1.21E-20	4.10E-19
ppa010515m|PACid:17660684	1.344589903	1.04E-07	1.44E-06
ppa007547m|PACid:17664654	1.34342503	1.46E-05	0.000143942
ppa020645m|PACid:17657493	1.340244718	3.14E-05	0.000291014
ppa001849m|PACid:17661386	1.336525471	6.79E-05	0.000588861
ppa006089m|PACid:17664790	1.332634304	2.12E-10	3.91E-09
ppa007068m|PACid:17660515	1.331322085	2.59E-05	0.000243489
ppa006888m|PACid:17658940	1.328951941	1.11E-21	4.02E-20
ppa011762m|PACid:17654448	1.326446537	1.55E-87	2.68E-85
ppa004473m|PACid:17668713	1.326090675	4.20E-17	1.21E-15
ppa002769m|PACid:17668075	1.324899661	1.32E-66	1.51E-64
ppa002424m|PACid:17646139	1.318318992	1.38E-09	2.34E-08
ppa010824m|PACid:17667635	1.317404748	5.72E-07	7.17E-06
ppa016117m|PACid:17665168	1.317313313	3.16E-06	3.47E-05
ppa006101m|PACid:17658859	1.314000418	1.73E-26	7.46E-25
ppa000393m|PACid:17659059	1.311292926	9.97E-05	0.000827503
ppa008288m|PACid:17661968	1.311292926	9.97E-05	0.000826935
ppa013712m|PACid:17662863	1.310046472	1.83E-07	2.44E-06
ppa007181m|PACid:17667360	1.308469055	1.01E-06	1.21E-05
ppa013099m|PACid:17662664	1.305863378	1.45E-05	0.000143677
ppa007610m|PACid:17653774	1.305863378	1.45E-05	0.00014356
ppa004751m|PACid:17664865	1.30533713	5.83E-21	2.00E-19
ppa003695m|PACid:17663413	1.300586463	1.54E-11	3.08E-10
ppa011474m|PACid:17661144	1.294374965	1.57E-08	2.38E-07
ppa003252m|PACid:17666502	1.291982694	3.79E-06	4.13E-05
ppa006064m|PACid:17650663	1.289877271	8.86E-26	3.73E-24
ppa012979m|PACid:17645788	1.28636691	1.21E-06	1.44E-05
ppa002401m|PACid:17652702	1.285945505	2.11E-05	0.000203004
ppa009110m|PACid:17643985	1.282044699	5.82E-08	8.29E-07
ppa010752m|PACid:17667911	1.277533976	1.23E-07	1.68E-06
ppa006421m|PACid:17647678	1.274752851	5.50E-06	5.82E-05
ppa006520m|PACid:17653922	1.269591608	4.52E-06	4.86E-05
ppa006726m|PACid:17649419	1.269446742	9.80E-05	0.000814077

ppa005787m|PACid:17656114	1.249516482	5.12E-36	2.84E-34
ppa001815m|PACid:17652194	1.243889357	4.43E-05	0.000398306
ppa010551m|PACid:17644046	1.243348611	1.73E-07	2.31E-06
ppa004526m|PACid:17664321	1.240593948	1.43E-07	1.93E-06
ppa018884m|PACid:17667611	1.237723317	3.62E-06	3.95E-05
ppa001577m|PACid:17647242	1.233050903	2.98E-05	0.000276696
ppa004528m|PACid:17642861	1.230043288	2.44E-06	2.75E-05
ppa016100m|PACid:17664699	1.228166096	7.78E-05	0.000665849
ppa001957m|PACid:17665341	1.225555214	1.27E-35	6.99E-34
ppa011368m|PACid:17665965	1.219807218	2.46E-12	5.18E-11
ppa005627m|PACid:17658765	1.218553963	5.22E-05	0.000461817
ppa007450m|PACid:17644102	1.21115612	3.81E-39	2.31E-37
ppa007970m|PACid:17655333	1.210505355	7.73E-13	1.70E-11
ppa020825m|PACid:17652478	1.208896233	9.02E-06	9.28E-05
ppa018908m|PACid:17667971	1.206394631	0.00011168	0.00091641
ppa008730m|PACid:17657884	1.204630679	2.13E-10	3.91E-09
ppa001094m|PACid:17663374	1.20179346	6.06E-06	6.38E-05
ppa010868m|PACid:17642911	1.198153543	4.97E-06	5.29E-05
ppa007013m|PACid:17665763	1.19733932	1.08E-65	1.18E-63
ppa000893m|PACid:17648620	1.195829334	1.06E-06	1.28E-05
ppa008159m|PACid:17658015	1.193924706	2.12E-49	1.65E-47
ppa007930m|PACid:17647761	1.189397957	4.99E-05	0.000444916
ppa018805m|PACid:17660504	1.185747039	2.22E-13	5.10E-12
ppa011844m|PACid:17652196	1.185745519	8.62E-06	8.89E-05
ppa012535m|PACid:17653141	1.175378368	5.72E-45	3.95E-43
ppa008537m|PACid:17656421	1.175317495	1.14E-35	6.31E-34
ppa006450m|PACid:17668721	1.173131704	3.96E-15	1.01E-13
ppa000157m|PACid:17655669	1.170761104	1.49E-05	0.000147335
ppa011865m|PACid:17664690	1.167763012	1.86E-33	9.67E-32
ppa007605m|PACid:17654073	1.167638569	1.98E-21	7.00E-20
ppa003639m|PACid:17643967	1.16616748	2.80E-40	1.75E-38
ppa012596m|PACid:17665075	1.164975106	3.08E-07	3.99E-06
ppa008769m|PACid:17660321	1.164488155	1.19E-227	7.55E-225
ppa004936m|PACid:17653005	1.163722272	3.88E-46	2.81E-44
ppa003097m|PACid:17644399	1.161854874	1.17E-06	1.40E-05
ppa010812m|PACid:17646625	1.152363593	3.02E-11	5.92E-10
ppa012919m|PACid:17668169	1.152240594	4.51E-12	9.30E-11
ppa003820m|PACid:17662926	1.151799165	2.46E-06	2.77E-05
ppa009090m|PACid:17646603	1.149183155	0.000100401	0.000832338
ppa002376m|PACid:17644619	1.149183155	0.000100401	0.000831768
ppa009289m|PACid:17654883	1.148089731	2.86E-08	4.25E-07
ppa005358m|PACid:17644469	1.145544153	7.76E-13	1.70E-11
ppa007655m|PACid:17650870	1.145446025	9.44E-06	9.68E-05
ppa010633m|PACid:17668143	1.14231642	5.46E-05	0.000481655

ppa013271m|PACid:17654126	1.141368557	5.70E-17	1.62E-15
ppa011969m|PACid:17668402	1.138973782	7.87E-77	1.09E-74
ppa003294m|PACid:17653373	1.136586071	3.98E-11	7.72E-10
ppa004404m|PACid:17645715	1.13290278	1.20E-19	3.88E-18
ppa001286m|PACid:17652397	1.12673023	0.00011544	0.000943422
ppa010399m|PACid:17651736	1.124098927	5.93E-06	6.25E-05
ppa009610m|PACid:17664431	1.123064809	4.85E-06	5.18E-05
ppa008618m|PACid:17667519	1.122676521	2.48E-14	6.05E-13
ppa007076m|PACid:17641383	1.116229807	1.59E-10	2.96E-09
ppa010928m|PACid:17656430	1.109218227	8.28E-06	8.55E-05
ppa003574m|PACid:17649082	1.100860042	2.51E-10	4.59E-09
ppa000383m|PACid:17648534	1.100536233	1.07E-17	3.17E-16
ppa004955m|PACid:17653696	1.097870513	2.28E-28	1.04E-26
ppa002531m|PACid:17666143	1.097595698	6.92E-21	2.36E-19
ppa003657m|PACid:17655447	1.096418578	1.84E-07	2.45E-06
ppa008417m|PACid:17654884	1.088884819	2.84E-09	4.66E-08
ppa010500m|PACid:17657393	1.087526792	1.57E-09	2.65E-08
ppa006262m|PACid:17656841	1.084417822	4.99E-14	1.19E-12
ppa004363m|PACid:17642533	1.084072034	1.92E-64	2.03E-62
ppa000665m|PACid:17650265	1.082287911	8.14E-05	0.000692026
ppa010473m|PACid:17651461	1.076830685	0	0
ppa004524m|PACid:17645797	1.065184506	6.29E-51	5.14E-49
ppa016011m|PACid:17644681	1.064130337	1.21E-05	0.000121399
ppa000637m|PACid:17650039	1.064130337	8.07E-08	1.13E-06
ppa002334m|PACid:17662100	1.061185501	4.25E-23	1.60E-21
ppa009971m|PACid:17654547	1.055521947	1.25E-08	1.92E-07
ppa013113m|PACid:17661996	1.053328052	4.48E-07	5.70E-06
ppb023395m|PACid:17663996	1.049113501	2.50E-05	0.000235515
ppa003730m|PACid:17647793	1.046422396	2.54E-08	3.80E-07
ppa013232m|PACid:17664682	1.04535675	1.02E-07	1.42E-06
ppa011227m|PACid:17656249	1.045280879	5.09E-11	9.73E-10
ppa008719m|PACid:17652237	1.044738315	1.36E-10	2.55E-09
ppa004451m|PACid:17656143	1.043954675	3.00E-10	5.44E-09
ppa007520m|PACid:17645059	1.042821627	7.51E-07	9.26E-06
ppa001692m|PACid:17655278	1.037937593	3.47E-08	5.08E-07
ppa001186m|PACid:17657648	1.036870779	4.06E-12	8.40E-11
ppa007844m|PACid:17649204	1.033757632	3.44E-05	0.000316486
ppa003265m|PACid:17646993	1.031305348	0	0
ppa007591m|PACid:17651555	1.030648305	1.22E-13	2.83E-12
ppa014856m|PACid:17658962	1.02888059	1.61E-40	1.02E-38
ppa021469m|PACid:17645617	1.02816353	3.95E-11	7.67E-10
ppa005350m|PACid:17648711	1.025526056	7.29E-09	1.14E-07
ppa005277m|PACid:17650866	1.021860139	1.19E-21	4.29E-20
ppa002527m|PACid:17647771	1.021072044	1.15E-06	1.37E-05

ppa006873m|PACid:17641291	1.017929458	1.43E-07	1.93E-06
ppa010918m|PACid:17660241	1.016397444	7.07E-05	0.000609831
ppa004924m|PACid:17651293	1.010637431	9.44E-10	1.62E-08
ppa008102m|PACid:17650351	1.007900105	2.53E-09	4.18E-08
ppa006860m|PACid:17658416	1.007508467	2.97E-08	4.40E-07
ppa017449m|PACid:17652377	1.004772957	6.57E-08	9.32E-07
ppa002140m|PACid:17657831	1.00305548	6.44E-05	0.000562285
ppa004926m|PACid:17648238	1.002715918	8.54E-15	2.13E-13
ppa005434m|PACid:17642225	1.002376484	9.57E-07	1.16E-05
ppa004555m|PACid:17659130	1.000680998	4.17E-33	2.13E-31
ppa012515m|PACid:17641010	1.000526051	2.71E-08	4.03E-07
ppa009635m|PACid:17650061	1.000421779	1.16E-05	0.000117187
ppa017472m|PACid:17648324	-11.67639789	4.49E-33	2.29E-31
ppa010154m|PACid:17662227	-11.43671154	4.06E-28	1.82E-26
ppa011095m|PACid:17659760	-11.16741815	1.87E-23	7.11E-22
ppa024234m|PACid:17653614	-11.09143539	2.74E-22	1.00E-20
ppa025143m|PACid:17643435	-10.83605036	8.63E-19	2.72E-17
ppa005901m|PACid:17648495	-10.76487159	6.47E-18	1.96E-16
ppa008626m|PACid:17650701	-10.6635581	9.48E-17	2.66E-15
ppa027164m|PACid:17643882	-10.37395266	7.79E-14	1.83E-12
ppa023758m|PACid:17664707	-10.2737956	5.84E-13	1.30E-11
ppa002363m|PACid:17661991	-10.01122726	6.41E-11	1.22E-09
ppa017806m|PACid:17649435	-9.968666793	1.25E-10	2.35E-09
ppa020537m|PACid:17643147	-9.882643049	4.80E-10	8.48E-09
ppa004406m|PACid:17657235	-9.789533645	1.84E-09	3.09E-08
ppa012577m|PACid:17663752	-9.636624621	1.38E-08	2.10E-07
ppa019204m|PACid:17648651	-9.583082768	2.69E-08	4.02E-07
ppa021383m|PACid:17655177	-9.583082768	2.69E-08	4.02E-07
ppa027063m|PACid:17656719	-9.525520809	5.27E-08	7.55E-07
ppa009350m|PACid:17665845	-9.46760555	1.03E-07	1.43E-06
ppa022459m|PACid:17648375	-9.46760555	1.03E-07	1.43E-06
ppa023213m|PACid:17664985	-9.342074668	3.95E-07	5.07E-06
ppa003161m|PACid:17657850	-9.273795599	7.73E-07	9.51E-06
ppa013235m|PACid:17656256	-9.204571144	1.51E-06	1.76E-05
ppa020980m|PACid:17643110	-9.204571144	1.51E-06	1.76E-05
ppa018706m|PACid:17655617	-9.129283017	2.96E-06	3.26E-05
ppa011770m|PACid:17664672	-9.129283017	2.96E-06	3.26E-05
ppa019215m|PACid:17657563	-9.129283017	2.96E-06	3.25E-05
ppa011822m|PACid:17642112	-9.052568051	5.79E-06	6.11E-05
ppb012748m|PACid:17656298	-8.968666793	1.13E-05	0.000114618
ppa022398m|PACid:17664899	-8.968666793	1.13E-05	0.000114522
ppa006577m|PACid:17662230	-8.882643049	2.22E-05	0.000211007
ppa010908m|PACid:17646716	-8.882643049	2.22E-05	0.000210841
ppa026487m|PACid:17650291	-8.882643049	2.22E-05	0.000210675

ppa015371m|PACid:17668071	-8.787902559	4.33E-05	0.000394222
ppa016881m|PACid:17641647	-8.787902559	4.33E-05	0.000393925
ppa012926m|PACid:17659376	-8.787902559	4.33E-05	0.000393629
ppa019793m|PACid:17654943	-8.689997971	8.48E-05	0.000718871
ppa006133m|PACid:17653743	-8.689997971	8.48E-05	0.000718368
ppa006231m|PACid:17642062	-8.689997971	8.48E-05	0.000717865
ppa001642m|PACid:17666712	-8.689997971	8.48E-05	0.000717362
ppa014982m|PACid:17659372	-8.689997971	8.48E-05	0.000716861
ppa022925m|PACid:17649685	-8.689997971	8.48E-05	0.00071636
ppa022323m|PACid:17648014	-8.689997971	8.48E-05	0.000715859
ppa001076m|PACid:17658297	-8.689997971	8.48E-05	0.00071536
ppa012767m|PACid:17665694	-5.267390811	2.13E-21	7.52E-20
ppa009513m|PACid:17652409	-5.143068155	3.01E-74	4.05E-72
ppa019171m|PACid:17640976	-5.089186697	4.54E-36	2.53E-34
ppa010410m|PACid:17662220	-4.958237476	1.09E-24	4.34E-23
ppa005351m|PACid:17667368	-4.943534078	2.07E-24	8.11E-23
ppa018214m|PACid:17646113	-4.875994902	2.67E-60	2.65E-58
ppa000438m|PACid:17649984	-4.833990049	1.83E-22	6.75E-21
ppa022767m|PACid:17643388	-4.796795079	1.34E-118	4.06E-116
ppa001116m|PACid:17662999	-4.689350936	1.17E-45	8.38E-44
ppa000513m|PACid:17644392	-4.600392541	4.94E-13	1.11E-11
ppa018679m|PACid:17664669	-4.542657467	1.76E-12	3.73E-11
ppa008693m|PACid:17655029	-4.527911357	3.71E-40	2.29E-38
ppa005514m|PACid:17648384	-4.338302877	0	0
ppa006985m|PACid:17643741	-4.249151693	5.07E-10	8.92E-09
ppa026927m|PACid:17661024	-4.20994824	8.96E-99	1.97E-96
ppa011331m|PACid:17654316	-4.175086707	1.77E-09	2.97E-08
ppa024899m|PACid:17656297	-4.007445996	2.03E-72	2.61E-70
ppa006439m|PACid:17652112	-3.972099684	3.92E-08	5.68E-07
ppa016155m|PACid:17642177	-3.89463894	8.43E-37	4.90E-35
ppa010418m|PACid:17660154	-3.891191909	1.86E-33	9.69E-32
ppa015791m|PACid:17652739	-3.881854045	1.34E-07	1.82E-06
ppa009312m|PACid:17646620	-3.881579507	9.75E-11	1.83E-09
ppa010114m|PACid:17648503	-3.816089732	1.92E-25	7.93E-24
ppa003791m|PACid:17666679	-3.721043599	2.85E-12	5.97E-11
ppa025027m|PACid:17650388	-3.68242831	1.54E-06	1.79E-05
ppa026846m|PACid:17646873	-3.628886457	2.82E-06	3.11E-05
ppa001278m|PACid:17667715	-3.554339272	1.04E-10	1.96E-09
ppa020189m|PACid:17669074	-3.527646985	4.11E-08	5.95E-07
ppa012188m|PACid:17654558	-3.51340924	9.38E-06	9.63E-05
ppa011103m|PACid:17644289	-3.479845795	4.10E-41	2.62E-39
ppa001083m|PACid:17661334	-3.450945153	1.70E-05	0.00016691
ppa017425m|PACid:17649190	-3.450945153	1.70E-05	0.000166775
ppa026370m|PACid:17643207	-3.450945153	1.70E-05	0.00016664

ppa004191m|PACid:17661889	-3.450945153	1.70E-05	0.000166505
ppa011670m|PACid:17653139	-3.426689389	2.74E-87	4.67E-85
ppa023839m|PACid:17658113	-3.416255477	8.37E-14	1.96E-12
ppa016764m|PACid:17665366	-3.375039431	2.39E-15	6.22E-14
ppa017311m|PACid:17656555	-3.328153326	8.73E-13	1.90E-11
ppa020227m|PACid:17655902	-3.319599289	5.58E-05	0.000490527
ppa026375m|PACid:17660441	-3.250374834	0.000100494	0.00083197
ppa003606m|PACid:17662014	-3.250374834	0.000100494	0.0008314
ppa016489m|PACid:17645649	-3.22490364	3.77E-08	5.47E-07
ppa025916m|PACid:17643357	-3.216218654	2.54E-06	2.83E-05
ppa012644m|PACid:17642088	-3.216218654	2.54E-06	2.83E-05
ppa018401m|PACid:17659385	-3.180512396	2.04E-39	1.25E-37
ppa019623m|PACid:17656091	-3.161333853	5.59E-29	2.61E-27
ppa011161m|PACid:17653520	-3.115954852	3.51E-77	5.05E-75
ppa004584m|PACid:17667862	-3.108694436	2.12E-07	2.81E-06
ppa015105m|PACid:17666420	-3.09647947	1.46E-54	1.26E-52
ppa018811m|PACid:17658697	-3.026152288	6.64E-07	8.26E-06
ppa006443m|PACid:17651269	-3.023207391	8.68E-10	1.50E-08
ppa008698m|PACid:17658085	-3.02101929	1.20E-12	2.57E-11
ppa025759m|PACid:17664483	-3.010492374	4.54E-57	4.16E-55
ppa008035m|PACid:17646541	-3.009984089	4.26E-11	8.22E-10
ppa008996m|PACid:17661578	-2.998278201	8.43E-25	3.40E-23
ppa013543m|PACid:17656915	-2.972811989	5.55E-08	7.93E-07
ppa005454m|PACid:17646757	-2.94873133	2.05E-18	6.33E-17
ppa012940m|PACid:17648106	-2.921078845	2.74E-110	7.05E-108
ppa004343m|PACid:17655679	-2.918708384	4.89E-14	1.17E-12
ppa004760m|PACid:17660630	-2.911167555	1.97E-11	3.91E-10
ppa005525m|PACid:17661365	-2.908670602	4.00E-10	7.14E-09
ppa022359m|PACid:17646410	-2.884326044	6.06E-71	7.72E-69
ppa005226m|PACid:17650776	-2.883254798	3.95E-44	2.68E-42
ppa022793m|PACid:17662739	-2.881579507	7.84E-05	0.000669324
ppa013714m|PACid:17658930	-2.848704223	0	0
ppa023694m|PACid:17659862	-2.842571195	1.96E-15	5.11E-14
ppa003852m|PACid:17640746	-2.842571195	2.49E-08	3.74E-07
ppa004220m|PACid:17645835	-2.834457066	3.89E-14	9.38E-13
ppa002995m|PACid:17662532	-2.77695757	7.49E-08	1.05E-06
ppa007015m|PACid:17653200	-2.770633307	6.33E-09	9.96E-08
ppa014424m|PACid:17668189	-2.764570056	3.99E-12	8.27E-11
ppa021292m|PACid:17643557	-2.752836987	8.48E-25	3.40E-23
ppa014874m|PACid:17662082	-2.747483466	1.90E-05	0.000183515
ppa008524m|PACid:17665430	-2.743421231	2.69E-19	8.57E-18
ppa012578m|PACid:17647690	-2.735517379	0	0
ppa012410m|PACid:17648372	-2.708217069	1.19E-19	3.88E-18
ppa010554m|PACid:17652465	-2.674976167	4.03E-10	7.17E-09

ppb008343m|PACid:17645990	-2.674976167	4.03E-10	7.16E-09
ppa004546m|PACid:17640549	-2.659509454	4.61E-06	4.95E-05
ppa018853m|PACid:17646870	-2.659509454	4.61E-06	4.94E-05
ppa012753m|PACid:17643104	-2.641916244	1.82E-133	6.11E-131
ppa022820m|PACid:17642063	-2.583006304	9.69E-05	0.000805177
ppa012021m|PACid:17648268	-2.560053926	1.91E-06	2.19E-05
ppa023197m|PACid:17666791	-2.551409941	5.29E-15	1.34E-13
ppa003209m|PACid:17646815	-2.549815938	5.77E-09	9.13E-08
ppa024024m|PACid:17651754	-2.519959719	4.57E-12	9.42E-11
ppa015987m|PACid:17667288	-2.51982748	3.25E-06	3.56E-05
ppa024020m|PACid:17662450	-2.51982748	3.25E-06	3.56E-05
ppa009719m|PACid:17642916	-2.506705037	3.78E-15	9.68E-14
ppa011784m|PACid:17655762	-2.476737244	5.94E-10	1.04E-08
ppa003501m|PACid:17653527	-2.472994069	3.89E-05	0.000355984
ppa003769m|PACid:17667166	-2.472994069	4.79E-13	1.08E-11
ppa001524m|PACid:17651971	-2.470180755	1.08E-34	5.82E-33
ppa013716m|PACid:17668576	-2.468672412	4.46E-23	1.67E-21
ppa007762m|PACid:17664645	-2.456774232	1.91E-07	2.53E-06
ppa003271m|PACid:17648917	-2.448460501	1.32E-06	1.56E-05
ppa008656m|PACid:17653704	-2.448317469	6.79E-09	1.06E-07
ppa020862m|PACid:17641430	-2.441867766	1.12E-15	2.97E-14
ppa010358m|PACid:17655665	-2.435386145	2.81E-16	7.69E-15
ppa003028m|PACid:17650848	-2.423211431	6.56E-05	0.000572099
ppa002707m|PACid:17642130	-2.412108168	5.58E-13	1.25E-11
ppa013347m|PACid:17647825	-2.411778643	7.79E-08	1.09E-06
ppa009792m|PACid:17647534	-2.408769878	7.20E-142	2.56E-139
ppa014227m|PACid:17642215	-2.405049345	3.74E-12	7.78E-11
ppa013580m|PACid:17665261	-2.404107074	9.65E-66	1.06E-63
ppa001456m|PACid:17652505	-2.401108186	7.35E-18	2.23E-16
ppa004728m|PACid:17662802	-2.396074987	9.29E-13	2.02E-11
ppa017780m|PACid:17648009	-2.394914534	1.55E-05	0.000153138
ppa015981m|PACid:17651437	-2.394914534	1.55E-05	0.000153013
ppa005153m|PACid:17663226	-2.394914534	1.55E-05	0.000152889
ppa001593m|PACid:17659510	-2.39358908	2.51E-11	4.94E-10
ppa006159m|PACid:17658937	-2.38389817	1.69E-10	3.14E-09
ppa005873m|PACid:17653466	-2.374395515	3.72E-06	4.06E-05
ppa009014m|PACid:17658559	-2.374395515	3.72E-06	4.06E-05
ppa003325m|PACid:17665947	-2.36983808	0.00011028	0.000906775
ppa012424m|PACid:17664591	-2.363768829	2.81E-10	5.12E-09
ppa011926m|PACid:17653795	-2.3593155	2.56E-31	1.27E-29
ppa005820m|PACid:17650912	-2.356551491	1.12E-77	1.63E-75
ppa000847m|PACid:17652982	-2.353610171	4.48E-27	1.96E-25
ppa007718m|PACid:17658499	-2.351261589	2.60E-05	0.00024367
ppa008211m|PACid:17667660	-2.350752073	1.58E-13	3.66E-12

ppa010226m|PACid:17648802	-2.304668895	4.33E-05	0.000394322
ppa001929m|PACid:17667125	-2.294815146	2.38E-30	1.15E-28
ppa012676m|PACid:17659347	-2.290516022	0	0
ppa015531m|PACid:17667384	-2.285802388	1.80E-14	4.43E-13
ppa020272m|PACid:17648626	-2.272339179	4.78E-13	1.08E-11
ppa014549m|PACid:17666569	-2.269498535	6.65E-107	1.64E-104
ppa003386m|PACid:17644768	-2.258734268	2.38E-07	3.13E-06
ppa000410m|PACid:17655991	-2.257589561	1.16E-29	5.50E-28
ppa007361m|PACid:17656180	-2.257336872	1.41E-08	2.15E-07
ppa013344m|PACid:17655147	-2.250713078	4.68E-24	1.82E-22
ppa005087m|PACid:17646195	-2.240663904	1.92E-21	6.83E-20
ppa005661m|PACid:17648054	-2.234420281	5.68E-09	9.03E-08
ppa011939m|PACid:17665722	-2.234420281	5.68E-09	9.02E-08
ppa023229m|PACid:17655442	-2.226256001	2.10E-12	4.44E-11
ppa006330m|PACid:17665504	-2.223678987	2.43E-123	7.94E-121
ppa009549m|PACid:17645131	-2.219713321	5.62E-10	9.85E-09
ppa023730m|PACid:17655479	-2.219050914	6.71E-06	7.00E-05
ppa003428m|PACid:17655256	-2.213779291	2.81E-05	0.000262125
ppa011379m|PACid:17660395	-2.208399149	2.11E-18	6.48E-17
ppa003062m|PACid:17659603	-2.208399149	0.000118637	0.000967585
ppa008461m|PACid:17662557	-2.2049606	1.56E-07	2.09E-06
ppa002982m|PACid:17666251	-2.200139614	9.17E-10	1.58E-08
ppa000591m|PACid:17643219	-2.194596864	2.65E-06	2.93E-05
ppa012777m|PACid:17654106	-2.189769813	1.44E-46	1.05E-44
ppa002188m|PACid:17644676	-2.183722549	6.20E-08	8.80E-07
ppa010637m|PACid:17648068	-2.180915785	1.10E-05	0.000111592
ppa012300m|PACid:17666491	-2.177000519	2.54E-07	3.34E-06
ppa008109m|PACid:17647324	-2.1652555	1.89E-32	9.54E-31
ppa011941m|PACid:17645010	-2.16142204	4.76E-93	9.27E-91
ppa016442m|PACid:17666463	-2.158253145	1.78E-28	8.22E-27
ppa007560m|PACid:17644482	-2.148967689	5.94E-13	1.32E-11
ppa025725m|PACid:17661109	-2.14599155	3.89E-18	1.18E-16
ppa007995m|PACid:17641249	-2.127755547	7.53E-05	0.00064704
ppa003709m|PACid:17658219	-2.126018526	1.57E-09	2.66E-08
ppa011991m|PACid:17668665	-2.125856363	7.04E-06	7.32E-05
ppb012922m|PACid:17647443	-2.10524316	2.65E-07	3.48E-06
ppa014457m|PACid:17645486	-2.102841709	2.92E-05	0.000272585
ppa011194m|PACid:17666926	-2.102841709	2.92E-05	0.000272375
ppa003520m|PACid:17664217	-2.097818551	4.53E-21	1.57E-19
ppa022289m|PACid:17666575	-2.091712314	1.09E-06	1.30E-05
ppa021072m|PACid:17660640	-2.089605837	1.14E-05	0.000115268
ppa010520m|PACid:17667772	-2.087519606	2.85E-48	2.18E-46
ppa003844m|PACid:17665673	-2.08591276	4.09E-09	6.60E-08
ppa008292m|PACid:17657649	-2.078720602	4.28E-07	5.46E-06

ppa013120m|PACid:17650896	-2.074176224	4.46E-06	4.83E-05
ppa016926m|PACid:17658682	-2.067645583	1.69E-07	2.26E-06
ppa005289m|PACid:17651288	-2.062797995	2.84E-33	1.47E-31
ppa002037m|PACid:17653542	-2.061784692	6.97E-17	1.96E-15
ppa012981m|PACid:17663566	-2.05367598	1.84E-05	0.000178805
ppa005183m|PACid:17650541	-2.044841675	4.15E-10	7.35E-09
ppa007670m|PACid:17658298	-2.038039361	4.18E-09	6.75E-08
ppa022938m|PACid:17655792	-2.023942685	1.68E-08	2.55E-07
ppa013792m|PACid:17645657	-2.01344212	1.23E-145	4.78E-143
ppa011666m|PACid:17640794	-2.009202849	1.06E-09	1.81E-08
ppa022572m|PACid:17662640	-2.002193102	2.69E-08	4.01E-07
ppa003447m|PACid:17645647	-2.000531869	6.69E-11	1.27E-09
ppa003405m|PACid:17646705	-2	4.51E-06	4.85E-05
ppa012290m|PACid:17652096	-1.994139349	1.77E-06	2.04E-05
ppa002576m|PACid:17640432	-1.989166194	6.94E-07	8.61E-06
ppa013612m|PACid:17648145	-1.989166194	6.94E-07	8.60E-06
ppa024858m|PACid:17661535	-1.97769322	4.74E-05	0.000424269
ppa013098m|PACid:17644157	-1.975958169	1.08E-12	2.34E-11
ppa006781m|PACid:17660697	-1.972811989	1.84E-05	0.000178752
ppa011825m|PACid:17640577	-1.972661291	2.67E-09	4.38E-08
ppa007003m|PACid:17656824	-1.965472317	2.81E-06	3.11E-05
ppb004684m|PACid:17656904	-1.965472317	2.81E-06	3.11E-05
ppa004143m|PACid:17650952	-1.960464165	6.21E-37	3.62E-35
ppa016008m|PACid:17657804	-1.959789573	4.41E-14	1.06E-12
ppa006974m|PACid:17648419	-1.959190785	6.78E-08	9.57E-07
ppa023716m|PACid:17640322	-1.9557261	4.85E-31	2.39E-29
ppa012344m|PACid:17665337	-1.946936649	4.15E-27	1.82E-25
ppa001969m|PACid:17653225	-1.943191764	2.13E-21	7.53E-20
ppa000186m|PACid:17658925	-1.937466775	7.56E-05	0.000647964
ppa005225m|PACid:17648154	-1.935204877	4.47E-06	4.83E-05
ppa010667m|PACid:17652306	-1.935204877	4.47E-06	4.83E-05
ppa004603m|PACid:17642304	-1.922984572	6.70E-12	1.37E-10
ppa002817m|PACid:17666720	-1.914213936	6.69E-08	9.47E-07
ppa017455m|PACid:17668032	-1.91409095	1.69E-07	2.26E-06
ppa011592m|PACid:17649337	-1.908670602	1.09E-06	1.30E-05
ppa001807m|PACid:17666087	-1.904310838	4.14E-10	7.35E-09
ppa1027217m|PACid:17660585	-1.903197567	1.81E-05	0.000175727
ppa009596m|PACid:17663788	-1.901911582	8.27E-34	4.40E-32
ppa013527m|PACid:17666226	-1.900631605	4.65E-05	0.000416327
ppa007297m|PACid:17648516	-1.898477215	1.94E-19	6.25E-18
ppa011225m|PACid:17641971	-1.886029236	1.63E-09	2.75E-08
ppa004508m|PACid:17657917	-1.870302487	1.13E-17	3.33E-16
ppa003309m|PACid:17657179	-1.869344742	1.69E-100	3.93E-98
ppa013260m|PACid:17654661	-1.867991475	7.70E-21	2.61E-19

ppa006630m|PACid:17648614	-1.862496476	7.33E-05	0.000631453
ppa006865m|PACid:17642694	-1.861763086	3.83E-28	1.73E-26
ppa005513m|PACid:17641943	-1.860773423	4.70E-45	3.28E-43
ppa011364m|PACid:17662916	-1.858982287	1.01E-11	2.03E-10
ppa022417m|PACid:17661696	-1.849395351	6.84E-06	7.13E-05
ppa021176m|PACid:17650413	-1.848455706	2.58E-07	3.39E-06
ppa013450m|PACid:17659501	-1.848455706	2.58E-07	3.39E-06
ppa002905m|PACid:17647247	-1.843274496	6.53E-07	8.13E-06
ppa007935m|PACid:17661463	-1.834457066	4.48E-05	0.000401899
ppa005474m|PACid:17655860	-1.826111785	4.03E-07	5.16E-06
ppa006408m|PACid:17654570	-1.826111785	4.03E-07	5.16E-06
ppa006678m|PACid:17660248	-1.824648846	0.00011508	0.000941752
ppa002973m|PACid:17658619	-1.824648846	0.00011508	0.000941114
ppa015633m|PACid:17647175	-1.82247448	1.51E-12	3.23E-11
ppa013471m|PACid:17661141	-1.818756737	1.17E-84	1.96E-82
ppa024429m|PACid:17659031	-1.79970135	6.55E-06	6.84E-05
ppa013004m|PACid:17646770	-1.797210036	9.17E-15	2.28E-13
ppa024173m|PACid:17663790	-1.793184022	2.26E-13	5.16E-12
ppa008712m|PACid:17656764	-1.790586709	6.67E-25	2.70E-23
ppa004060m|PACid:17648282	-1.781956979	2.23E-16	6.15E-15
ppa010706m|PACid:17668387	-1.780928063	6.23E-24	2.40E-22
ppa013173m|PACid:17644641	-1.77770269	2.17E-14	5.32E-13
ppa008318m|PACid:17664157	-1.768587573	6.16E-27	2.68E-25
ppa005278m|PACid:17662214	-1.766656316	8.64E-10	1.50E-08
ppa016382m|PACid:17659484	-1.766656316	8.64E-10	1.49E-08
ppa013222m|PACid:17652268	-1.764765351	2.95E-98	6.37E-96
ppa003941m|PACid:17656295	-1.756728849	3.28E-11	6.39E-10
ppa013426m|PACid:17643234	-1.749978131	3.29E-10	5.92E-09
ppa025989m|PACid:17655273	-1.741966275	1.57E-05	0.000154385
ppa010800m|PACid:17644802	-1.733840461	1.37E-07	1.86E-06
ppa018866m|PACid:17642245	-1.733840461	1.37E-07	1.85E-06
ppb010244m|PACid:17667542	-1.73306114	7.42E-14	1.75E-12
ppa013305m|PACid:17655745	-1.731448589	2.31E-06	2.61E-05
ppa006431m|PACid:17660508	-1.724067367	1.91E-10	3.53E-09
ppa000104m|PACid:17664785	-1.713879285	1.91E-09	3.21E-08
ppa007832m|PACid:17645388	-1.710283552	2.91E-10	5.29E-09
ppa002045m|PACid:17653156	-1.709877767	8.59E-07	1.05E-05
ppb023648m|PACid:17651305	-1.702818728	6.06E-19	1.92E-17
ppa000687m|PACid:17666037	-1.697776329	7.84E-08	1.10E-06
ppa004537m|PACid:17646997	-1.693734694	3.20E-07	4.13E-06
ppa004042m|PACid:17652434	-1.690205661	4.80E-08	6.91E-07
ppa001941m|PACid:17666152	-1.68993206	2.46E-13	5.58E-12
ppa005617m|PACid:17664214	-1.68968165	5.03E-37	2.95E-35
ppa008401m|PACid:17665391	-1.676152843	3.99E-74	5.30E-72

ppa026720m|PACid:17641519	-1.654771967	3.40E-12	7.09E-11
ppa011177m|PACid:17647931	-1.650283549	1.24E-174	5.76E-172
ppa016719m|PACid:17642109	-1.650253961	5.67E-05	0.000497273
ppa012415m|PACid:17661286	-1.646747193	4.51E-07	5.73E-06
ppa005393m|PACid:17645759	-1.646747193	4.51E-07	5.72E-06
ppa007393m|PACid:17645810	-1.645104736	3.42E-05	0.000315126
ppb013010m|PACid:17669289	-1.640801102	2.71E-15	7.00E-14
ppa005302m|PACid:17641883	-1.640771116	2.34E-09	3.89E-08
ppa005542m|PACid:17643009	-1.636301198	1.26E-11	2.54E-10
ppa011819m|PACid:17646328	-1.630619892	3.71E-29	1.74E-27
ppa014016m|PACid:17649797	-1.628489876	5.76E-09	9.13E-08
ppa000094m|PACid:17663888	-1.626215133	6.82E-07	8.46E-06
ppa008528m|PACid:17645732	-1.623436649	7.58E-06	7.88E-05
ppa000120m|PACid:17646271	-1.623436649	7.58E-06	7.87E-05
ppa011167m|PACid:17648743	-1.623269557	6.69E-23	2.49E-21
ppa013661m|PACid:17647188	-1.618024521	4.71E-58	4.45E-56
ppa018565m|PACid:17663039	-1.617701117	1.04E-21	3.76E-20
ppa011297m|PACid:17668585	-1.614837296	5.19E-05	0.00046057
ppa004188m|PACid:17642110	-1.614837296	5.19E-05	0.000460232
ppa011808m|PACid:17642082	-1.611901573	5.30E-09	8.47E-08
ppa025434m|PACid:17653233	-1.603201619	1.89E-05	0.000183082
ppa017535m|PACid:17644582	-1.603201619	1.89E-05	0.000182936
ppa021082m|PACid:17658469	-1.598760048	1.14E-05	0.000115539
ppa003719m|PACid:17646583	-1.593952798	1.06E-16	2.94E-15
ppa018041m|PACid:17648846	-1.593083461	2.20E-55	1.96E-53
ppb019987m|PACid:17662744	-1.592038018	2.97E-09	4.86E-08
ppa006306m|PACid:17660572	-1.589520783	3.90E-11	7.59E-10
ppa002999m|PACid:17642517	-1.584962501	7.84E-05	0.000669248
ppa011176m|PACid:17667021	-1.584962501	7.84E-05	0.000668775
ppa012001m|PACid:17655514	-1.555302832	2.27E-35	1.24E-33
ppa012769m|PACid:17668256	-1.555122281	1.17E-07	1.60E-06
ppa004540m|PACid:17663706	-1.555122281	1.17E-07	1.60E-06
ppa012153m|PACid:17651488	-1.547214143	2.58E-05	0.000242415
ppa002969m|PACid:17660420	-1.541585438	2.10E-06	2.39E-05
ppa009639m|PACid:17650220	-1.540594789	6.87E-217	3.95E-214
ppa023832m|PACid:17652091	-1.533969709	1.24E-09	2.12E-08
ppa006244m|PACid:17654150	-1.527573828	1.07E-17	3.18E-16
ppa011280m|PACid:17658150	-1.524439129	8.01E-118	2.30E-115
ppa012480m|PACid:17649977	-1.521652198	3.55E-08	5.19E-07
ppa013223m|PACid:17661976	-1.520927233	1.74E-59	1.70E-57
ppa004542m|PACid:17646649	-1.520761248	2.26E-18	6.95E-17
ppa017910m|PACid:17657395	-1.515164761	1.11E-19	3.62E-18
ppb001660m|PACid:17644700	-1.504208009	2.33E-07	3.07E-06
ppa005890m|PACid:17653764	-1.498428453	1.26E-05	0.000126061

ppa013737m|PACid:17652924	-1.495599044	4.00E-09	6.51E-08
ppa012231m|PACid:17669102	-1.494666575	3.34E-57	3.10E-55
ppa025855m|PACid:17651626	-1.492097808	9.50E-05	0.000791255
ppa003877m|PACid:17667033	-1.490274416	1.27E-07	1.73E-06
ppa010072m|PACid:17666074	-1.477753257	3.40E-50	2.72E-48
ppa007105m|PACid:17648564	-1.475569019	1.14E-07	1.57E-06
ppa027208m|PACid:17648540	-1.471433555	3.07E-07	3.99E-06
ppa000698m|PACid:17661207	-1.46880125	5.11E-05	0.00045364
ppa015407m|PACid:17662915	-1.466978428	1.37E-06	1.61E-05
ppa004027m|PACid:17653607	-1.46579802	8.47E-05	0.000718487
ppa011393m|PACid:17641491	-1.463383818	2.20E-229	1.47E-226
ppa008063m|PACid:17660580	-1.454393323	1.66E-05	0.0001629
ppa012954m|PACid:17669077	-1.452564681	2.73E-43	1.83E-41
ppb023427m|PACid:17642344	-1.448498607	1.71E-15	4.48E-14
ppa000114m|PACid:17657476	-1.441867766	7.53E-05	0.000647083
ppa022581m|PACid:17657666	-1.441223608	1.55E-17	4.53E-16
ppa013500m|PACid:17651996	-1.438005061	8.95E-06	9.22E-05
ppa003417m|PACid:17647472	-1.435999862	6.16E-09	9.71E-08
ppa011792m|PACid:17668531	-1.434430034	3.65E-17	1.05E-15
ppa011284m|PACid:17667864	-1.431991246	6.16E-82	9.80E-80
ppa009332m|PACid:17665631	-1.427794632	6.88E-77	9.79E-75
ppa013939m|PACid:17653268	-1.426324136	3.05E-21	1.06E-19
ppa019192m|PACid:17657896	-1.420640206	7.95E-06	8.23E-05
ppa008201m|PACid:17653320	-1.41223689	2.16E-05	0.000206352
ppa026106m|PACid:17644929	-1.41223689	2.16E-05	0.000206189
ppa007604m|PACid:17655140	-1.412184096	3.71E-15	9.51E-14
ppa005590m|PACid:17651297	-1.411583282	2.73E-45	1.93E-43
ppa010223m|PACid:17641541	-1.407160687	1.23E-82	2.01E-80
ppa013469m|PACid:17668624	-1.405329946	1.82E-10	3.37E-09
ppa010167m|PACid:17663484	-1.381846974	6.06E-13	1.34E-11
ppa007255m|PACid:17644606	-1.371934461	7.76E-42	5.04E-40
ppa002536m|PACid:17661766	-1.366408538	4.57E-05	0.000409455
ppa003262m|PACid:17650664	-1.351448938	1.30E-05	0.000129866
ppa026813m|PACid:17659943	-1.351028458	2.04E-10	3.77E-09
ppa013050m|PACid:17663148	-1.347239183	2.70E-08	4.02E-07
ppa003802m|PACid:17664861	-1.340167213	2.63E-09	4.32E-08
ppa008491m|PACid:17668236	-1.33909216	2.38E-08	3.57E-07
ppa011898m|PACid:17666678	-1.337210012	2.31E-25	9.42E-24
ppa012754m|PACid:17640801	-1.334692189	2.17E-50	1.76E-48
ppa024854m|PACid:17643685	-1.326704891	3.40E-08	5.00E-07
ppa023263m|PACid:17650445	-1.326704891	3.40E-08	4.99E-07
ppa014463m|PACid:17643699	-1.320367581	9.41E-07	1.14E-05
ppa010755m|PACid:17647934	-1.312400254	7.26E-32	3.62E-30
ppa012790m|PACid:17662237	-1.310454224	5.01E-14	1.19E-12

ppa007608m|PACid:17660560	-1.306352289	1.43E-05	0.000141893
ppa013445m|PACid:17651358	-1.306338911	2.23E-66	2.50E-64
ppb015588m|PACid:17669011	-1.29712046	1.08E-08	1.68E-07
ppa009369m|PACid:17653310	-1.295715875	1.64E-18	5.10E-17
ppa001230m|PACid:17642722	-1.295167027	1.25E-05	0.000125201
ppa009169m|PACid:17646352	-1.294815146	6.04E-08	8.59E-07
ppa001169m|PACid:17646296	-1.290677161	2.11E-22	7.77E-21
ppa012984m|PACid:17660219	-1.288438766	4.38E-82	7.06E-80
ppa020389m|PACid:17662447	-1.286152068	5.51E-07	6.93E-06
ppa011733m|PACid:17642487	-1.284997876	1.09E-14	2.69E-13
ppa014312m|PACid:17668747	-1.284387361	5.93E-15	1.49E-13
ppa003828m|PACid:17648549	-1.284274189	5.82E-06	6.15E-05
ppa006898m|PACid:17667208	-1.281598011	0.000118605	0.000967979
ppa005333m|PACid:17654348	-1.276617378	1.78E-05	0.000173687
ppa006523m|PACid:17651815	-1.276038131	1.05E-18	3.29E-17
ppa012018m|PACid:17651778	-1.274916029	5.08E-06	5.40E-05
ppa012321m|PACid:17644906	-1.268947736	0.000103054	0.000850831
ppa009209m|PACid:17645568	-1.268947736	0.000103054	0.00085025
ppa012943m|PACid:17640751	-1.268946803	5.47E-05	0.000482059
ppa008800m|PACid:17666840	-1.267531748	1.17E-57	1.10E-55
ppa010552m|PACid:17664768	-1.266194747	2.37E-06	2.67E-05
ppa026365m|PACid:17664884	-1.266194747	2.37E-06	2.67E-05
ppa000447m|PACid:17669265	-1.262505559	2.61E-09	4.29E-08
ppa000464m|PACid:17654385	-1.258976189	8.95E-05	0.00074825
ppa001285m|PACid:17643754	-1.258515867	1.35E-05	0.000134227
ppa003623m|PACid:17655180	-1.258010506	4.75E-05	0.000424584
ppa001064m|PACid:17645439	-1.257563725	1.10E-06	1.32E-05
ppa011974m|PACid:17659537	-1.254520191	1.27E-12	2.72E-11
ppa004887m|PACid:17666330	-1.249119659	2.19E-05	0.000209327
ppa012068m|PACid:17668884	-1.249119659	2.19E-05	0.000209162
ppa012040m|PACid:17645392	-1.247624967	3.07E-16	8.39E-15
ppa007818m|PACid:17651655	-1.240955152	1.02E-05	0.000104167
ppa004422m|PACid:17663012	-1.240955152	1.02E-05	0.000104079
ppa005083m|PACid:17668625	-1.239629374	1.31E-09	2.23E-08
ppa009033m|PACid:17656433	-1.23786383	7.27E-07	8.98E-06
ppa005536m|PACid:17669156	-1.236887215	6.72E-05	0.000583315
ppa012294m|PACid:17659439	-1.234213286	8.65E-28	3.84E-26
ppa023464m|PACid:17640779	-1.231790141	2.81E-29	1.32E-27
ppa006618m|PACid:17654435	-1.222820283	6.83E-17	1.92E-15
ppa022557m|PACid:17666541	-1.221740061	1.90E-06	2.18E-05
ppa001464m|PACid:17648541	-1.213832948	4.13E-07	5.28E-06
ppa000095m|PACid:17647200	-1.211126387	4.36E-05	0.000395368
ppa006390m|PACid:17667419	-1.210912963	5.66E-21	1.95E-19
ppa012075m|PACid:17661699	-1.210697464	6.64E-09	1.04E-07

ppa010234m|PACid:17655357	-1.206175528	1.67E-07	2.24E-06
ppa010610m|PACid:17668774	-1.202691993	1.16E-36	6.67E-35
ppa009300m|PACid:17656541	-1.199226437	6.77E-08	9.58E-07
ppa013537m|PACid:17662155	-1.190135897	1.53E-09	2.60E-08
ppa003836m|PACid:17656446	-1.188017069	3.00E-101	7.25E-99
ppa010645m|PACid:17645748	-1.181391884	1.45E-11	2.90E-10
ppa013861m|PACid:17646890	-1.180328666	3.36E-16	9.12E-15
ppa005220m|PACid:17662951	-1.174186242	3.53E-10	6.33E-09
ppa026099m|PACid:17656664	-1.173907138	3.40E-66	3.77E-64
ppa000012m|PACid:17653487	-1.171888402	2.08E-06	2.37E-05
ppa011911m|PACid:17640898	-1.169925001	1.81E-05	0.000175667
ppa003878m|PACid:17649430	-1.167162235	8.35E-07	1.02E-05
ppa005719m|PACid:17645371	-1.167162235	8.35E-07	1.02E-05
ppb008326m|PACid:17655951	-1.16411938	3.38E-05	0.000311736
ppa013621m|PACid:17640980	-1.160926468	3.65E-10	6.53E-09
ppa008412m|PACid:17651171	-1.160397735	1.80E-11	3.57E-10
ppa015669m|PACid:17644565	-1.159151606	2.43E-62	2.51E-60
ppa012736m|PACid:17650435	-1.158718068	1.34E-06	1.58E-05
ppa002952m|PACid:17660414	-1.158372618	6.33E-05	0.000553079
ppa007663m|PACid:17648450	-1.158117395	6.22E-06	6.53E-05
ppa004563m|PACid:17659708	-1.153782473	2.47E-11	4.87E-10
ppa004818m|PACid:17650538	-1.152071923	5.45E-05	0.00048102
ppa013510m|PACid:17655432	-1.143702177	3.18E-301	2.56E-298
ppa012587m|PACid:17661828	-1.143680445	3.46E-07	4.45E-06
ppa012093m|PACid:17659725	-1.140084782	2.99E-07	3.88E-06
ppa024131m|PACid:17646265	-1.138386176	6.40E-07	7.98E-06
ppa011378m|PACid:17649715	-1.13580949	2.97E-40	1.84E-38
ppa013051m|PACid:17652655	-1.135191707	2.79E-80	4.33E-78
ppa001786m|PACid:17643860	-1.134986575	6.38E-06	6.68E-05
ppa004908m|PACid:17658484	-1.133995144	7.55E-05	0.000647863
ppa001686m|PACid:17644861	-1.133995144	7.55E-05	0.000647403
ppa000932m|PACid:17650256	-1.128359651	1.19E-05	0.000119434
ppa005275m|PACid:17650471	-1.127705439	4.13E-11	7.99E-10
ppa012757m|PACid:17660422	-1.126330824	2.57E-05	0.000241595
ppa004431m|PACid:17667682	-1.123254568	5.57E-05	0.00049107
ppa013311m|PACid:17647503	-1.120411244	2.96E-45	2.08E-43
ppa006479m|PACid:17650905	-1.114403055	4.86E-07	6.14E-06
ppa001730m|PACid:17651686	-1.10524316	7.67E-05	0.000657415
ppa012119m|PACid:17661304	-1.103490728	5.21E-14	1.23E-12
ppa004334m|PACid:17660758	-1.102980232	6.64E-07	8.25E-06
ppa002793m|PACid:17652246	-1.102780474	4.14E-06	4.50E-05
ppa013635m|PACid:17658458	-1.102260728	1.10E-13	2.56E-12
ppa012965m|PACid:17660893	-1.101541978	1.61E-12	3.44E-11
ppb021781m|PACid:17649828	-1.101209385	1.84E-18	5.70E-17

ppa006016m|PACid:17655899	-1.097554341	2.62E-10	4.78E-09
ppa006662m|PACid:17641716	-1.096813775	8.83E-15	2.20E-13
ppa013455m|PACid:17647298	-1.0932219	2.63E-06	2.93E-05
ppa023976m|PACid:17650999	-1.091712314	1.65E-05	0.000162208
ppa016754m|PACid:17655837	-1.086796562	9.05E-19	2.85E-17
ppa003707m|PACid:17649749	-1.085654863	2.29E-10	4.19E-09
ppa015170m|PACid:17658294	-1.084606873	3.96E-09	6.45E-08
ppa010397m|PACid:17657053	-1.070486221	1.66E-05	0.000162884
ppa010980m|PACid:17644257	-1.065661759	5.39E-09	8.59E-08
ppa010028m|PACid:17647481	-1.064934898	3.58E-05	0.000328689
ppa009471m|PACid:17641095	-1.064934898	3.58E-05	0.00032844
ppb018874m|PACid:17664579	-1.062267044	5.86E-15	1.47E-13
ppa020061m|PACid:17664797	-1.061784692	3.16E-07	4.09E-06
ppa012933m|PACid:17660914	-1.05697471	4.05E-101	9.60E-99
ppa000667m|PACid:17664698	-1.055015474	1.96E-06	2.23E-05
ppa008582m|PACid:17663444	-1.054698729	5.16E-08	7.39E-07
ppa012063m|PACid:17648528	-1.047683151	3.59E-06	3.93E-05
ppa020372m|PACid:17644030	-1.04368674	1.40E-61	1.42E-59
ppa011201m|PACid:17656874	-1.042840618	9.81E-16	2.61E-14
ppa022781m|PACid:17643273	-1.039340892	6.59E-06	6.88E-05
ppa005938m|PACid:17653821	-1.037874652	1.09E-07	1.50E-06
ppa008306m|PACid:17644234	-1.035501584	4.85E-06	5.18E-05
ppa012553m|PACid:17653229	-1.03360719	1.06E-06	1.27E-05
ppa009288m|PACid:17661086	-1.031823318	5.10E-08	7.33E-07
ppa012003m|PACid:17650941	-1.029945401	3.77E-08	5.46E-07
ppa011243m|PACid:17668345	-1.02976275	1.04E-16	2.91E-15
ppa011828m|PACid:17667327	-1.029343962	4.83E-05	0.000431171
ppa009193m|PACid:17658815	-1.028372498	1.85E-09	3.11E-08
ppa022680m|PACid:17666197	-1.027055351	8.89E-06	9.16E-05
ppa001534m|PACid:17664605	-1.026956477	1.78E-11	3.55E-10
ppa012251m|PACid:17651383	-1.023482565	3.26E-36	1.83E-34
ppa013088m|PACid:17666531	-1.013512697	9.32E-16	2.48E-14
ppa012891m|PACid:17656691	-1.008377349	1.67E-07	2.24E-06
ppa004884m|PACid:17647851	-1.006687575	6.29E-17	1.78E-15
ppa013414m|PACid:17643791	-1.00414622	4.87E-11	9.35E-10
ppa011067m|PACid:17647317	-1.003545863	2.50E-26	1.07E-24

Supplementary Table S6 The correlation between the differentially expressed proteins and genes in the whole library. The X is the expression quantity of the differentially expressed proteins and the Y is the expression quantity of genes in the whole library.

[a]Spot No	Protein	Gene	X	Y
31	ppa005617	ppa005617m|PACid:17664214	1.831877241	-1.68968165
21	ppa011448	ppa011448m|PACid:17646081	-6.64385619	5.292282633
27	ppa007748	ppa007748m|PACid:17651918	2.939226578	-0.335708945
2	ppa003391	ppa003391m|PACid:17655389	1.545968369	0.06529146
30	ppa010413	ppa010413m|PACid:17642780	-2.13093087	0.623933732
18	ppa004554	ppa004554m|PACid:17664318	1.422233001	-0.359081093
32	ppa007242	ppa007242m|PACid:17668944	1.859969548	-0.762276683
1	gi|2460186	ppa012651m|PACid:17642469	-1.765534746	-6.882643049
11	ppa004430	ppa004430m|PACid:17665888	-1.350497247	0.388301771
23	gi|374671153	ppa010748m|PACid:17648023	1.378511623	2.231099117
35	ppa006226	ppa006226m|PACid:17663253	-1.411426246	-0.155119067
6	ppa010479	ppa010479m|PACid:17642602	2.807354922	0.705700372
9	ppa007696	ppa007696m|PACid:17669094	1.604071324	-0.421741167
19	ppa004726	ppa004726m|PACid:17655111	-1.510961919	0.124847848
20	ppa011112	ppa011112m|PACid:17657156	1.765534746	0.801900492
33	gi|33772153	ppa013109m|PACid:17643063	1.339137385	-1.090828978
28	gi|381283804	ppa009729m|PACid:17666637	1.438292852	0.638245233
8	ppa007154	ppa007186m|PACid:17659073	1.459431619	-0.116917752
16	gi|4650846	ppa007772m|PACid:17658645	1.280956314	-0.561145471

[a] Numbering corresponds to the 2–DE gel in Fig. 2.

Supplementary Fig. S1 Morphology changes before and after the dormancy break in Japanese apricot. A represents the endodormancy of the flower bud, and B represents green tips, showing that the flower buds of Japanese apricot have broken their dormancy.

Supplementary Fig. S2 Experimental design associated with dormancy release in Japanese apricot**.**

Supplementary Fig. S3 Experimental process of digital gene expression profile.

Supplementary Fig. S4 The images of the original gels and the matchsets appearing in Fig. 2.

Supplementary Fig. S5 Histogram showing pathway enrichment analysis for differentially expressed genes in Japanese apricot after GA_4 treatment (10 days).

Supplementary Fig. S6 Real-time qPCR validations of tag-mapped genes are shown in A, and B represents the scatter plots which indicated the cycle threshold (Ct) value of qRT-PCR analysis and the $\log_2$ transcripts per million tags value of differentially expressed genes.

Supplementary Fig. S7 Correlation between the differentially expressed proteins and genes in the whole library.

Supplementary Fig. S8 The principal component analysis of the whole library genes (A), differentially expressed genes (B) and differentially expressed proteins (C). Each spot represents an expressed gene; the black spot represents an up-regulated gene, and the red spot represents down-regulated gene. In A, B and C, the cumulative contribution rate (including the contribution rate of the first principal component and the second principal component) was more than 0.999999, which showed the significance between the up-/down-regulated genes/proteins and the expressed genes/proteins.

Supplementary Fig. S1

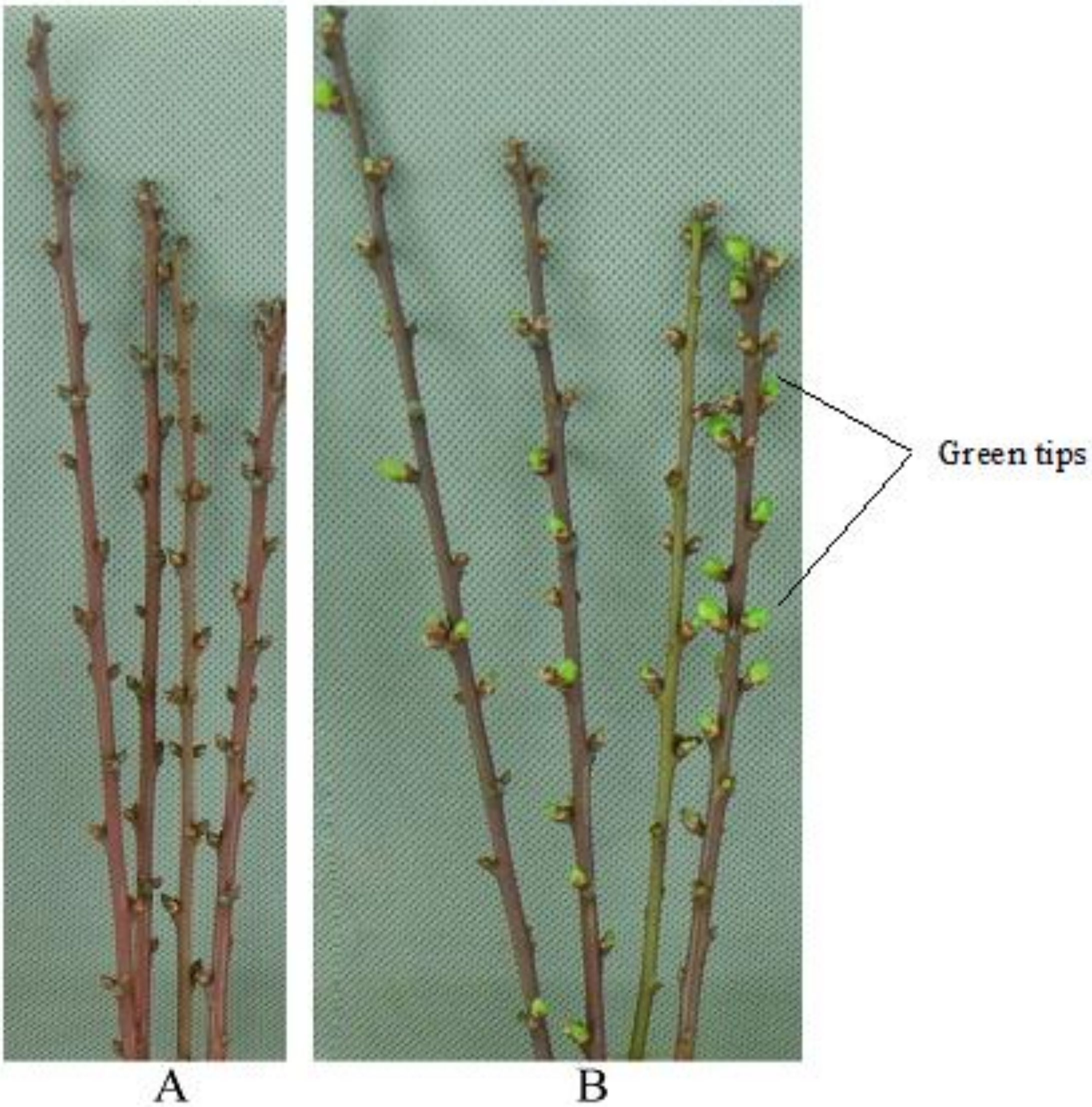

Supplementary Fig. S2

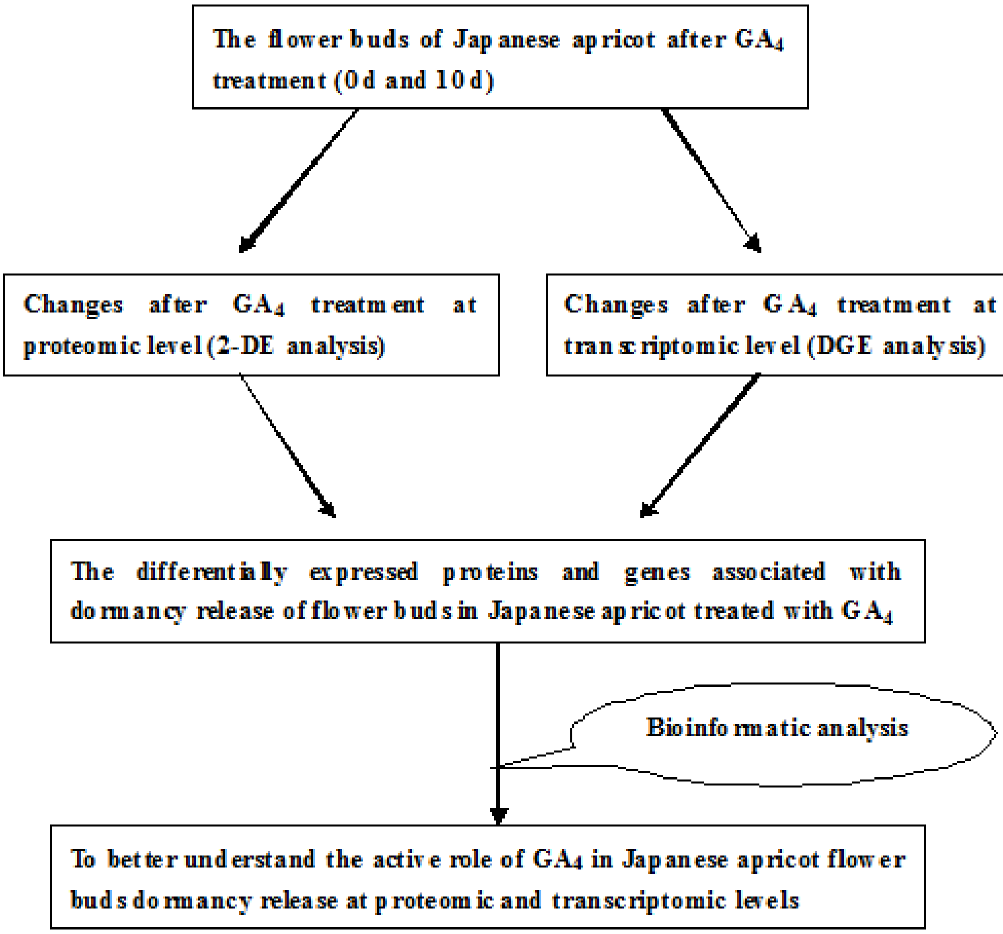

Supplementary Fig. S3

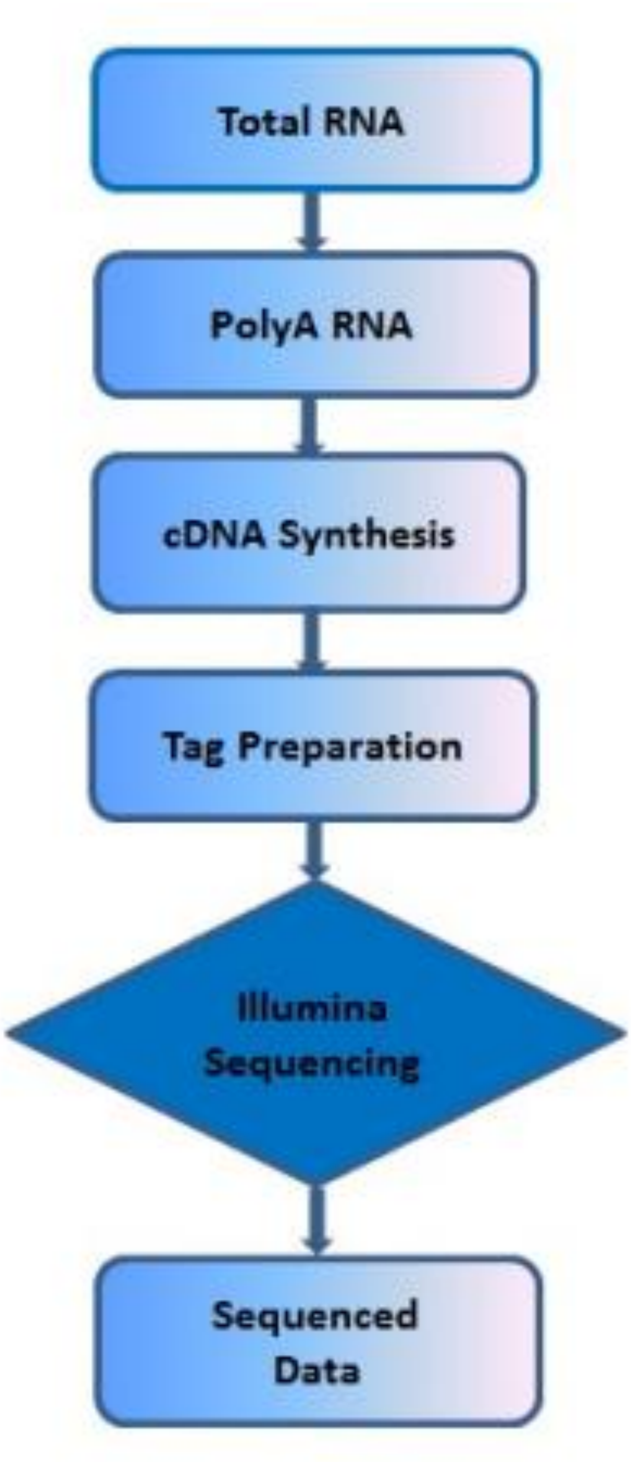

Supplementary Fig. S4

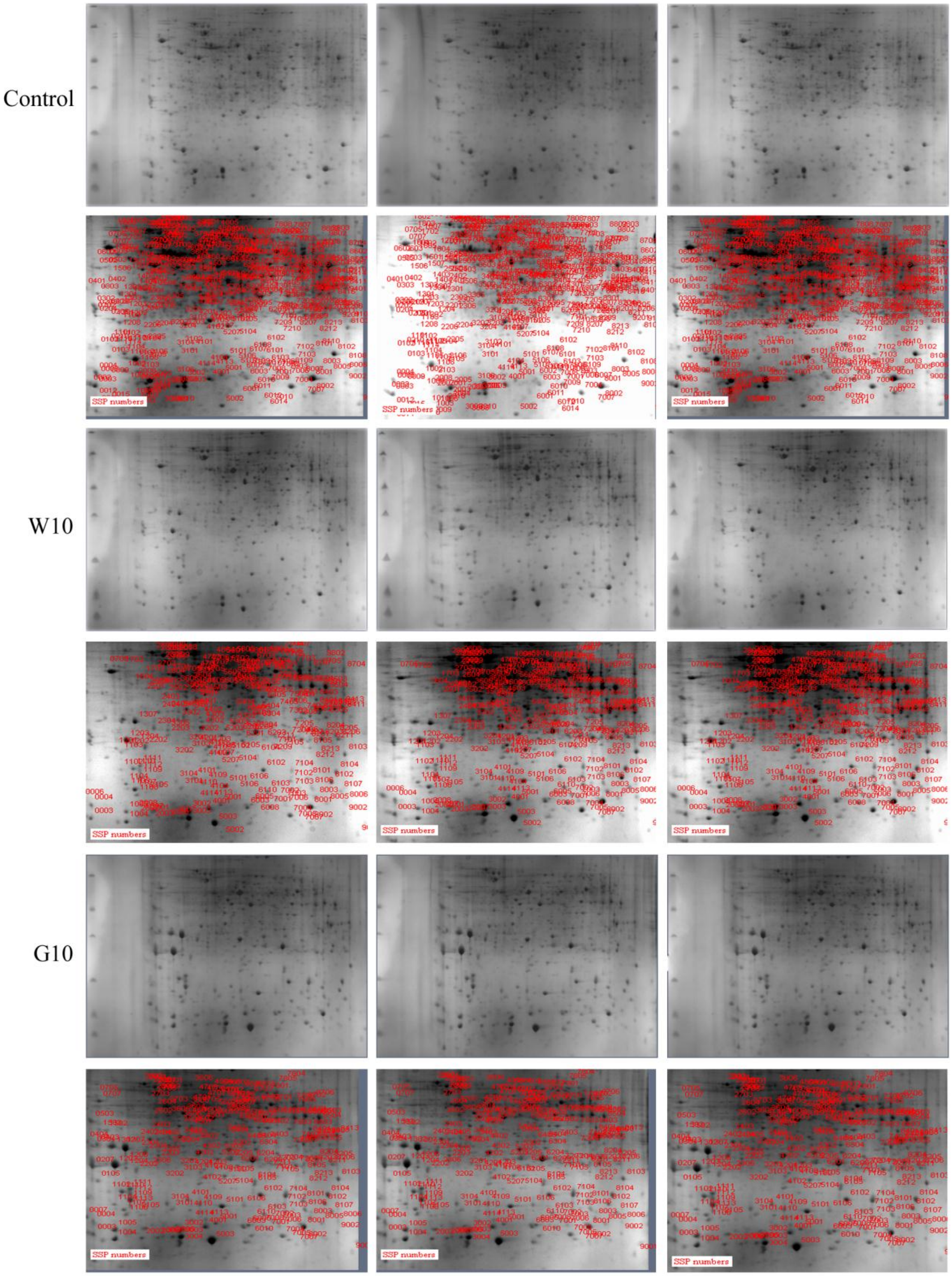

Supplementary Fig. S5

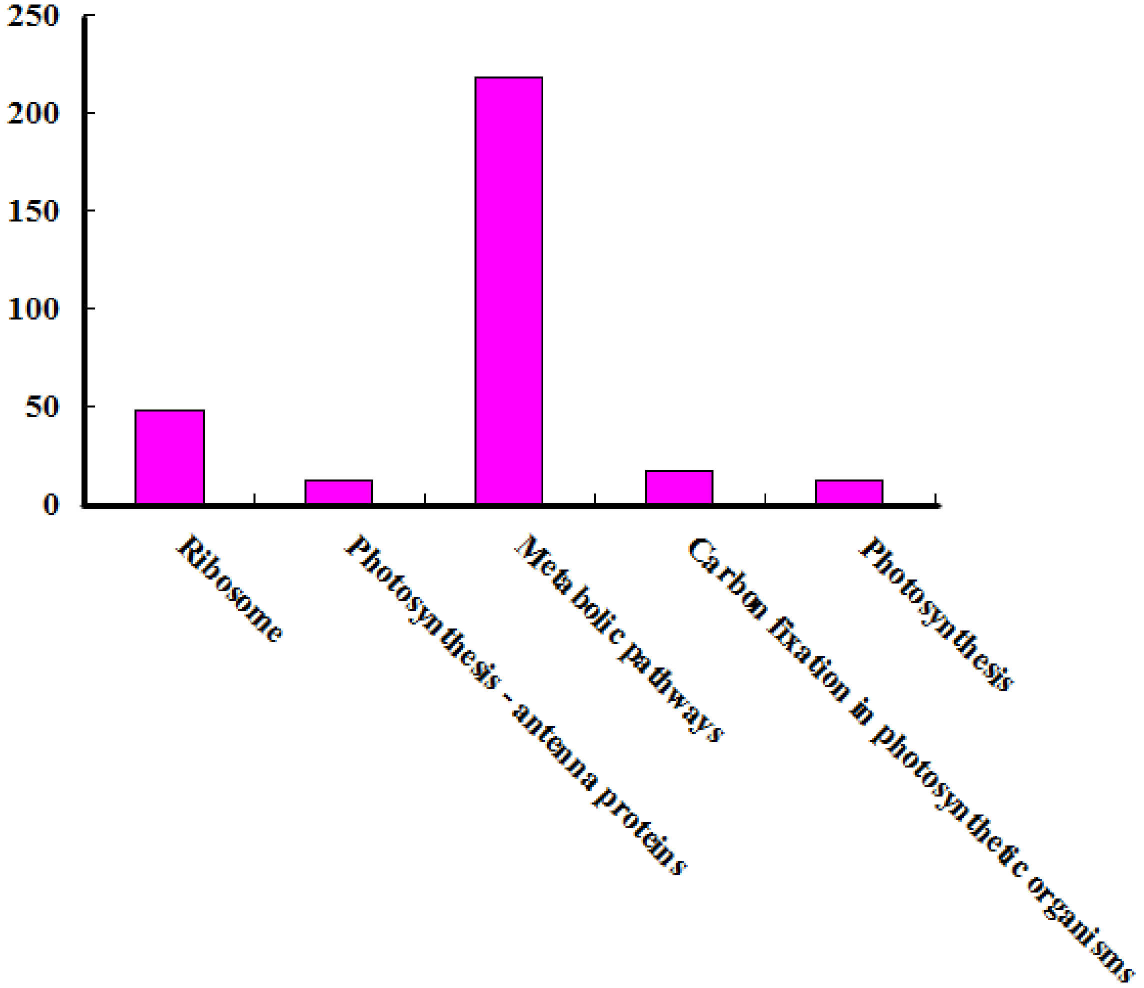

Supplementary Fig. S6

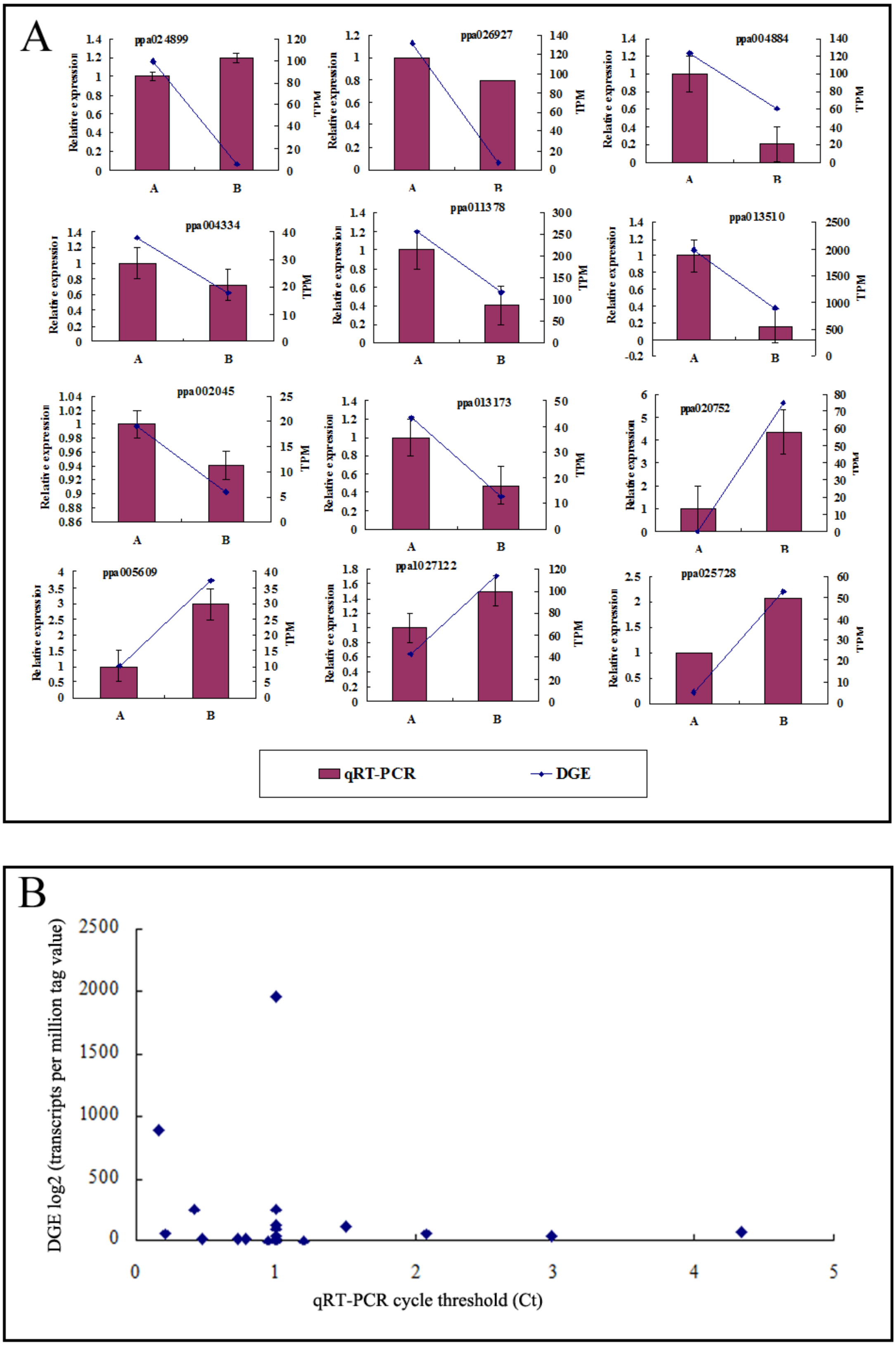

Supplementary Fig. S7

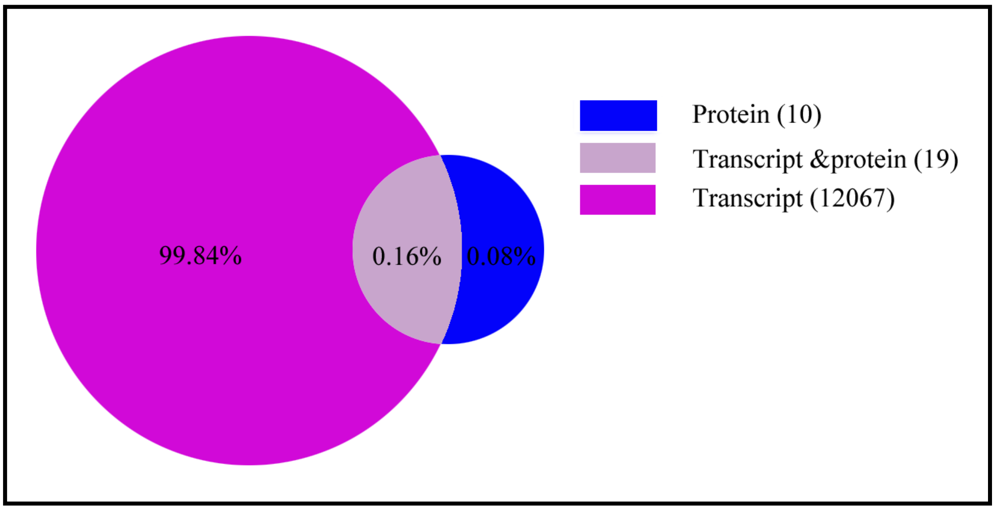

Supplementary Fig. S8

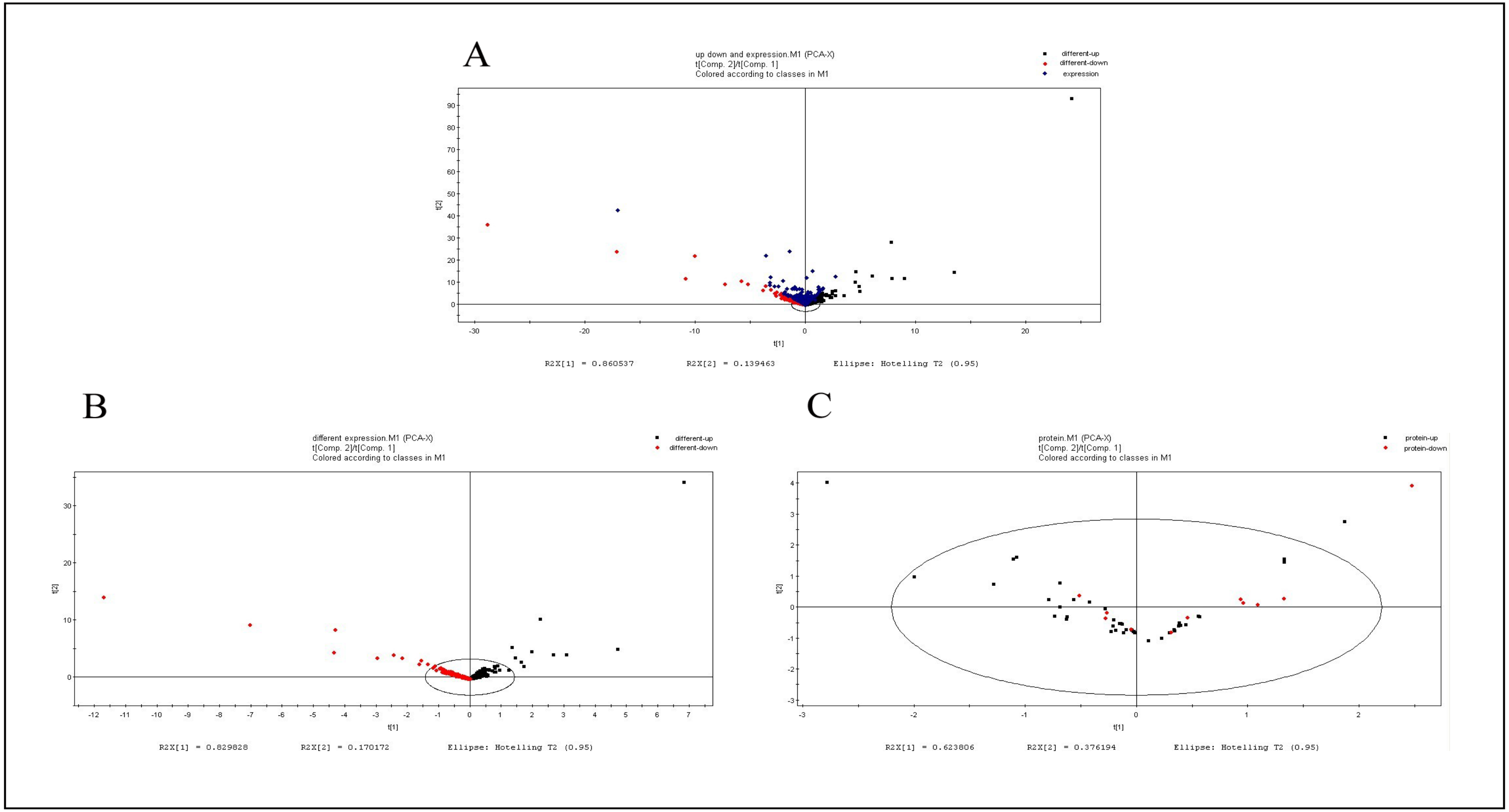

Supplement: Supplementary Data [file supp_ert284_jexbot092262_file001.pdf]
